# Access to highly substituted oxazoles by the reaction of α-azidochalcone with potassium thiocyanate

**DOI:** 10.3762/bjoc.16.178

**Published:** 2020-08-31

**Authors:** Mysore Bhyrappa Harisha, Pandi Dhanalakshmi, Rajendran Suresh, Raju Ranjith Kumar, Shanmugam Muthusubramanian

**Affiliations:** 1 Department of Organic Chemistry, School of Chemistry, Madurai Kamaraj University, Madurai-625 021, Tamil Nadu, India; 2 Eurofins-Advinus Limited, Phase II, Peenya Industrial Area, Bangalore-560 058, India; 3 Department of Inorganic and Physical Chemistry, Indian Institute of Science (IISc), Bangalore-560 012, India

**Keywords:** aminothiazole, oxazole, potassium persulfate, thiazole, vinyl azide

## Abstract

The reactivity of α-azidochalcones has been explored for the preparation of highly substituted oxazoles via a 2*H*-azirine intermediate. The azidochalcones, when treated with potassium thiocyanate in the presence of potassium persulfate, lead to 2,4,5-trisubstituted oxazoles in good yields. Incidentally, 2-aminothiazoles are the products when ferric nitrate is employed instead of persulfate in the above reaction.

## Introduction

Vinyl azide is one of the most versatile and potent building blocks explored in the synthesis of several heterocycles [1–5]. It can undergo photolysis or thermolysis to afford highly strained three-membered 2*H*-azirine, which can act as the precursor for nitrogen heterocycles. As a part of our synthetic design towards the construction of five-membered heterocycles, we have previously reported the synthesis of highly substituted imidazoles [6], indoles [7] and pyrroles [8] starting from different azidochalcones. In continuation, employing α-azidochalcones as the precursor [9], we herein report the preparation of highly substituted oxazoles and thiazoles.

**Figure 1:**
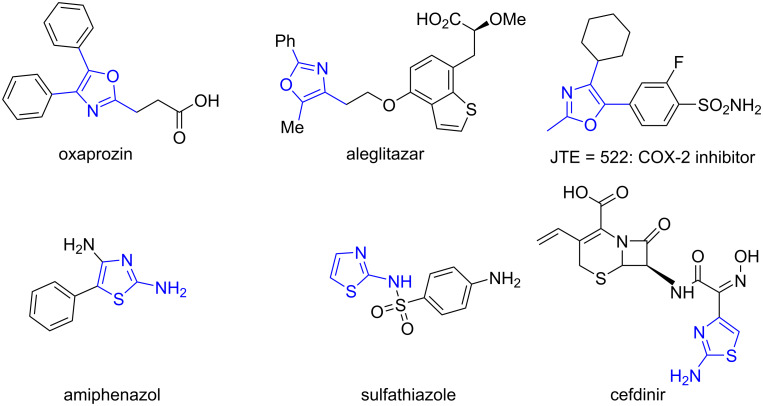
Examples of biologically active oxazole and aminothiazole scaffolds.

Oxazoles are ubiquitously found in various natural products [10–14], pharmaceuticals [15–18], functional materials [19–20] as well as in several organic building blocks [21–26]. Some oxazoles play a significant role in biological properties such as TRPV1 antagonistical activity, antifungal, analgesic, anti-inflammatory, antiproliferative, antileukemia, anticancer [27–32] and enzyme inhibitory activities [33–42]. 2,4,5-Trisubstituted oxazoles are embedded in some natural products and pharmaceuticals with a broad range of biological activities prompting the development of efficient synthetic strategies for this useful heterocycle [43–44] (Figure 1).

**Scheme 1:**
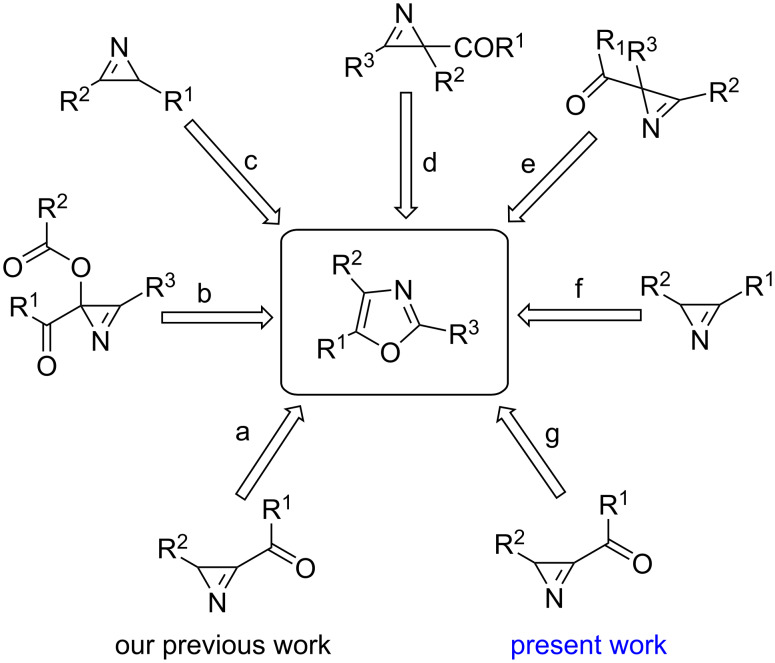
Strategies for the synthesis of 2,4,5-trisubstituted oxazole from azirine. a) I_2_, PPh_3_; b) NaH, 1*H*-pyrazole; c) 2-bromoacetyl bromide, NaN_3_; d) heating; e) *t*-BuOK; f) Ph-CHO, visible light; g) KSCN, K_2_S_2_O_8_.

In the recent past, the readily accessible 2*H*-azirine, an efficient source of nitrogen, was employed as starting material for the synthesis of oxazole with various coupling partners such as aldehyde, trifluoroacetic acid, etc. [8,45–50] (Scheme 1).

Thiazole is a common structural motif that is found in a wide variety of naturally existing alkaloids and a number of pharmaceutically active compounds [51–53]. 2-Aminothiazole has a thiourea-like character with a tendency to modulate promiscuously multiple biological targets. Thiazole derivatives also exhibit a broad spectrum of biological activities including antiviral, antiprion, anti-inflammatory, antimicrobial, anitubercular, psychotropic and anticancer [54–60]. The marketed cancer drug dasatinib [61] continues to prove its worth.

In this work, it is shown that highly substituted oxazoles and aminothiazoles could be accessed directly from the reaction of substituted *α*-azidochalcones with potassium thiocyanate. Thiocyanate is a known ambident reagent with two potential sites of attack, enabling the selective and efficient construction of C–C and C–N bonds towards biologically important heterocyclic skeletons [62–64].

## Results and Discussion

Previously, we have reported the TMSOTf-catalyzed synthesis of highly substituted imidazoles from α-azidochalcones under mild conditions [65]. As a sequel, the synthesis of oxazoles with an arylimino substituent has been accomplished in this work. The biologically important arylimino group [66–69] integrated with a highly substituted oxazole skeleton with a thiol group is expected to have potential synergetic bioactivity [70].

During the exploration of the reactivity of azidochalcones with thiocyanate in the presence of the oxidizing agent, **1i** was chosen as the model α-azidochalcone to react with potassium thiocyanate **2** in the presence of several oxidants and metal salts (Table 1). The initial attempts employing iodine, CAN and ZnCl_2_ upon refluxing in acetonitrile for 6 hours did not yield any product (Table 1, entries 1, 2 and 4).

**Table 1:** Optimisation studies.^a^

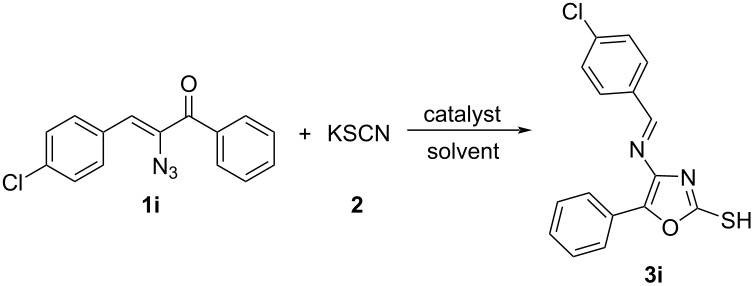				

Entry	Catalyst	Solvent	Conditions	Yield^b^

1	I_2_	CH_3_CN	KSCN, reflux, 6 h	nr^c^
2	CAN	CH_3_CN	KSCN, reflux, 6 h	nr
3	FeCl_3_	CH_3_CN	KSCN, reflux, 6 h	–^d^
4	ZnCl_2_	CH_3_CN	KSCN, reflux, 6 h	nr
**5**	**K_2_S_2_O_8 _(0.5 equiv)**	**CH_3_CN**	KSCN, reflux, 6 h	**97**^e^
6	K_2_S_2_O_8_ (1 equiv)	CH_3_CN	KSCN, reflux, 6h	96
7	K_2_S_2_O_8_ (0.1 equiv)	CH_3_CN	KSCN, reflux, 6 h	85
8	K_2_S_2_O_8_	CH_3_CN	KSCN, rt, 24 h	nr
9	–	CH_3_CN	KSCN, reflux, 6 h	nr
10	K_2_S_2_O_8_	ethanol	KSCN, reflux, 6 h	nr
11	K_2_S_2_O_8_	MeOH	KSCN, reflux, 6 h	nr
12	K_2_S_2_O_8_	water	KSCN, reflux, 6 h	nr
13	K_2_S_2_O_8_	1,4-dioxan	KSCN, reflux, 6 h	nr
14	K_2_S_2_O_8_	THF	KSCN, reflux, 6 h	nr
15	K_2_S_2_O_8_	toluene	KSCN, reflux, 6 h	nr
16	K_2_S_2_O_8_	DMF	KSCN, 120 °C, 6 h	nr
17	K_2_S_2_O_8_	DCE	KSCN, reflux, 6 h	nr
18^e^	K_2_S_2_O_8_	CH_3_CN	NH_4_SCN, reflux, 6 h	65
19^e^	K_2_S_2_O_8_	CH_3_CN	NH_4_SCN, rt, 18 h	nr
20^e^	K_2_S_2_O_8_	DCE	NH_4_SCN, 90 °C, 6 h	nr

^a^Reaction conditions: α-azidochalcone **1** (1 equiv), potassium thiocyanate **2** (3 equiv), oxidant/metal salt (0.5 equiv) in various solvents (2 mL); ^b^isolated yield after recrystallization; ^c^no reaction; ^d^isolated product was identified as 2-aminothiazole; ^e^reaction conditions: α-azidochalcone **1i** (1 equiv), ammonium thiocyanate **2a** (2 equiv), potassium persulfate (0.5 equiv) solvent (2 mL).

**Scheme 2:**
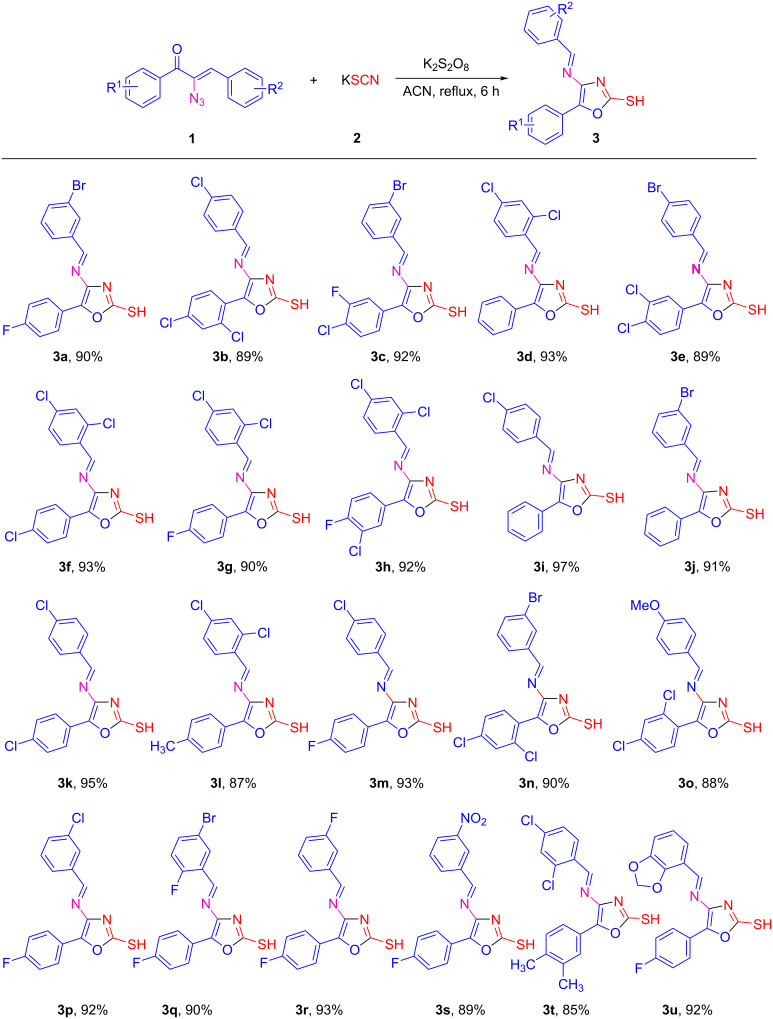
Scope of the α-azidochalcones. The reactions were carried out at reflux temperature, using **1** (1 mmol), **2** (3 mmol), potassium persulfate (0.5 mmol) in acetonitrile (2 mL) for 6 h. Yields refer to pure products after recrystallization.

When potassium persulfate (K_2_S_2_O_8_) is employed, highly substituted oxazole **3i** has been obtained. With the observation that potassium persulfate can efficiently catalyze the reaction to furnish highly substituted oxazole **3i**, we carried out the reaction of **1i** and **2** in acetonitrile in the presence of various equivalents of potassium persulfate_,_ in an attempt to evaluate the catalytic efficiency of persulfate. The reaction was found occurring most efficiently with 1 equiv of **1i**, 3 equiv of potassium thiocyanate **2,** and 0.5 equiv of potassium persulfate with a yield of 97% of **3i**. The yellow solid obtained after filtration of the reaction mixture afforded pure product **3i** without the requirement of any further work-up or purification protocol. The analytically pure sample was obtained by recrystallization from cold diethyl ether. However, in the absence of potassium persulfate (Table 1, entry 9), no product was observed indicating that potassium persulfate is essential to facilitate the reaction. After determining the optimal amount of persulfate, we have examined various solvents (Table 1, entries 10–17) to study the outcome of the reaction. These solvent screening studies indicated that acetonitrile is a suitable solvent for this reaction. Having established conditions for the high-yielding synthesis of oxazole **3,** the scope of this transformation with various α-azidochalcones was explored (Scheme 2).

**Scheme 3:**
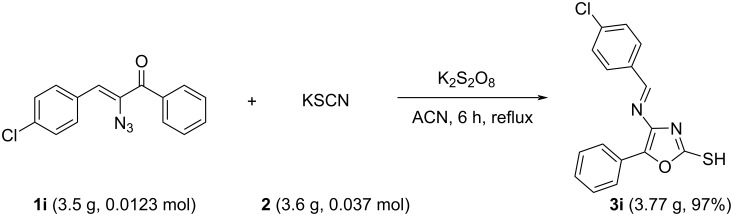
Large-scale synthesis of **3i**.

As shown in Scheme 2, an efficient conversion of α-azidochalcones **1a–u** to highly substituted functionalized oxazoles **3a–u** has been achieved with both electron-poor and electron-rich aryl substituents. Both nitro- and bromo-substituted systems can be further functionalized to get a new set of products. The tolerance of the reaction for a variety of aryl substituents illustrates the generality of this method for the preparation of a range of highly substituted oxazoles. Further, the scalability of the reaction using an optimized protocol was investigated by conducting the reaction on a multigram scale (Scheme 3). It was found that the reaction between **1i** and **2** on a multigram scale proceeded to afford **3i** in 97% yield.

**Figure 2:**
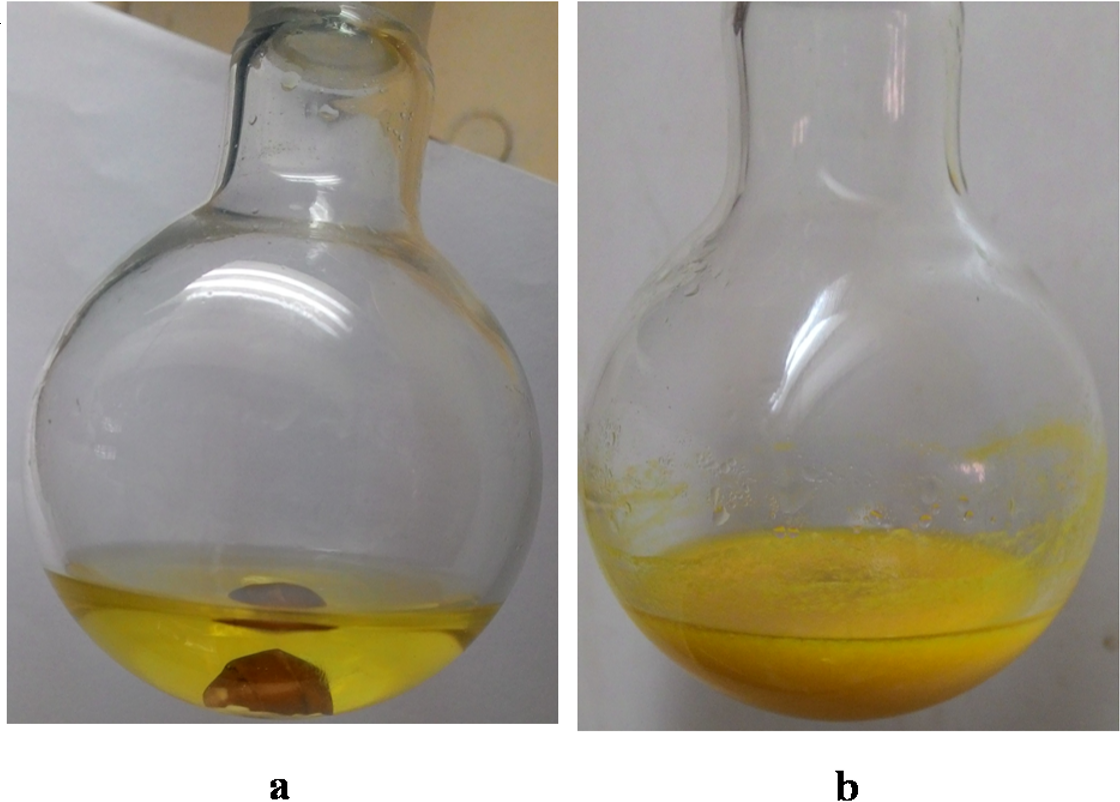
Large-scale synthesis of **3i**. a) At the start of the reaction, b) after the reaction.

In Figure 2, photograph a shows the reaction mixture just at the start of the reaction and b is the photograph after the completion of the reaction.

**Scheme 4:**
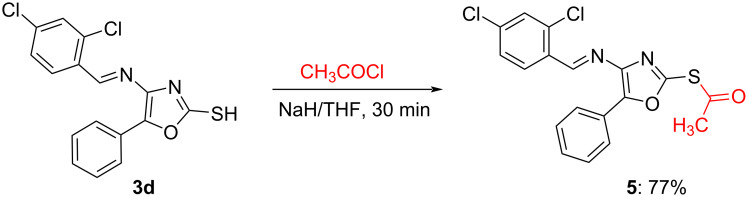
Acetyl derivative of **3d**.

**Scheme 5:**
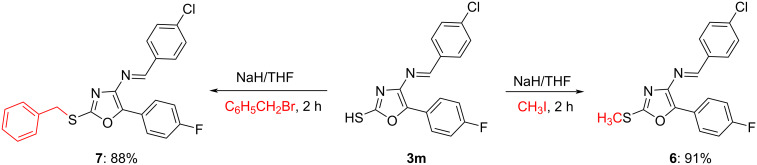
Synthesis of *S*-methyl/benzylated products **6** and **7**.

Further, the utility of the thiol group in **3** for the generation of a library of compounds was demonstrated by the simple acetylation and alkylation (Scheme 4 and Scheme 5). The acetylation of the thiol group in **3d** proceeded smoothly with acetyl chloride in the presence of sodium hydride to afford **5** in good yield.

**Figure 3:**
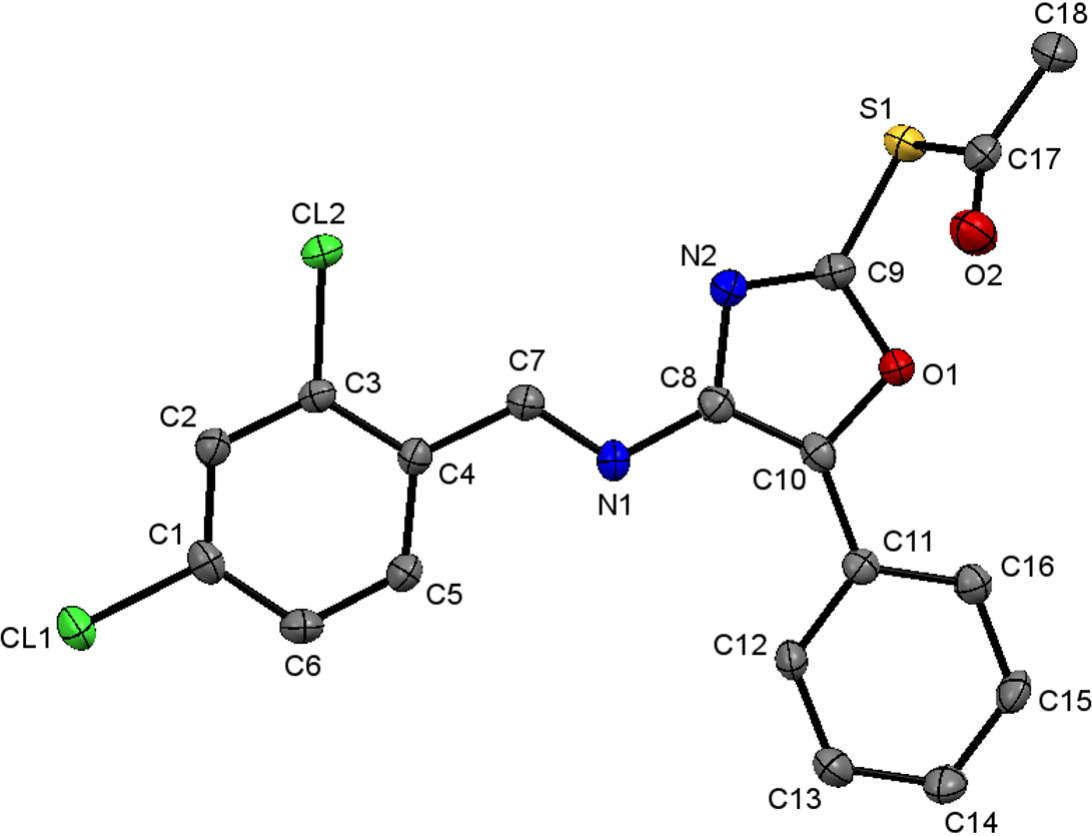
ORTEP diagram of compound **5**.

The structure of the product and the site of acetylation was confirmed by X-ray crystallography of a single crystal of **5** [71] (Figure 3).

The methylated and benzylated derivatives **6** and **7** were also obtained from **3m**. S-Methylation of **3m** was achieved in 91% yield with methyl iodide in the presence of NaH/THF and the S-benzylation has been carried out by a similar procedure (Scheme 5).

**Scheme 6:**
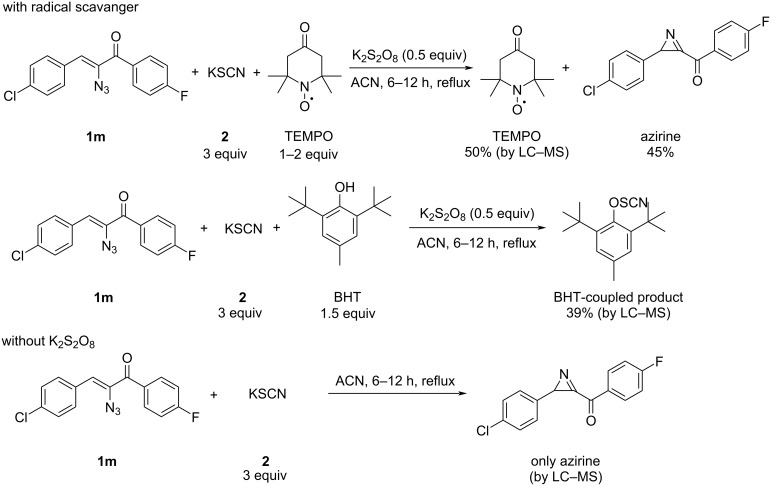
Control experiments.

To derive the mechanism of the reaction, a few control experiments have been executed (Scheme 6). Initially, the reaction of **1m** with potassium thiocyanate **2** under the optimal conditions in the presence of TEMPO furnished azirine and TEMPO, while the same reaction in the presence of BHT afforded the BHT-coupled thiocyanate product (*SO*-(2,6-di-*tert*-butyl-4-methylphenyl) (thioperoxocyanate)). These observations unambiguously indicate that the reaction proceeds through a radical pathway. Potassium persulfate helps to generate a thiocyanate radical and in the absence of potassium persulfate the reaction did not proceed. This experiment supports the role of potassium persulfate as an oxidant.

**Scheme 7:**
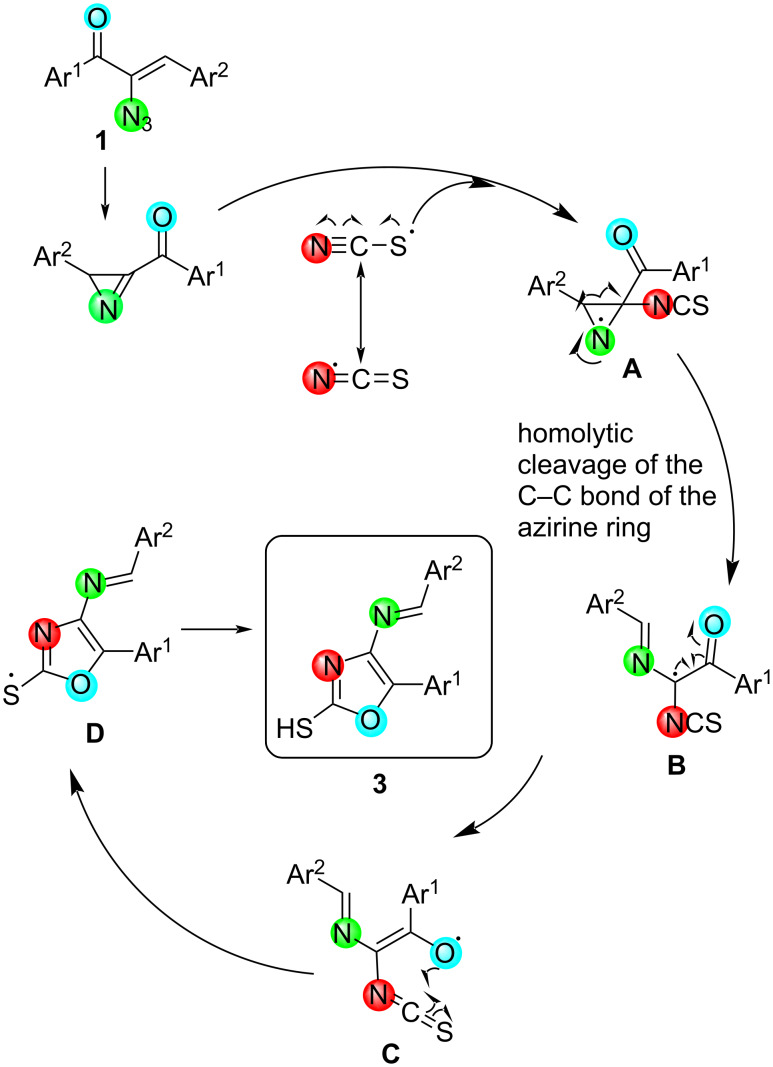
Plausible mechanism proposed for the formation of 2,4,5-trisubstituted oxazoles **3**.

Based on these experiments, the following plausible mechanism for the formation of 2,4,5-trisubstituted oxazoles can be proposed (Scheme 7). It is known that the thiocyanate radical is generated from potassium thiocyanate by the reaction with potassium persulfate [72]. The N-end of thiocyanate radical reacts with the C=N bond to give the intermediate **A** which undergoes homolytic cleavage yielding **B**. Subsequent cyclisation results in the oxazole ring.

During these optimization trials, it was interesting to note the formation of 2-aminothiazole, when ferric chloride was employed along with thiocyanate (Table 1, entry 3). There is a report pertaining to this transformation with Fe(II) salts [73]. We further wanted to capitalize on this result and optimize the methodology to access a series of 2-aminothiazoles as the reported methods [74–79] to access 2,4,5-trisubstituted aminothiazoles, especially 4-aroyl-2-amiothiazoles, suffered from low yields, harsh reaction conditions, expensive and detrimental metal precursors as well as the pollution concerning α-halocarbonyl compounds.

Initially, we started the reaction with (*Z*)-2-azido-1,3-bis(4-chlorophenyl)prop-2-en-1-one (**1d**) and commercially available potassium thiocyanate (**2**) as a representative model system. To optimize the best reaction condition, we began this study by performing the reaction with ferric chloride in acetonitrile at 80 °C for 6 h (Table 2, entry 1).

**Table 2:** Screening of iron salts and solvents^a^.

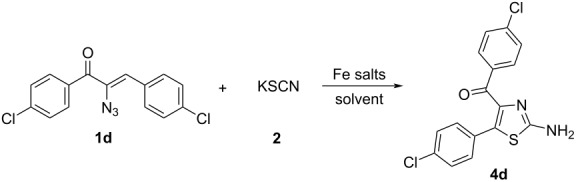					

Entry	Solvent	Catalyst	Temp	Time [h]	Yield^b^ [%]

1	CH_3_CN	FeCl_3_	reflux	6	85
2	DCE	FeCl_3_	reflux	5	nr
3	toluene	FeCl_3_	reflux	5	nr
4	CH_3_CN	FeCl_3_	rt	18	nr
5	CH_3_CN	Fe_2_O_3_	reflux	6	10
6	CH_3_CN	Fe(NO_3_)_3_	reflux	3	60
**7**	**CH_3_CN**	**Fe(NO_3_)_3_**	reflux	**6**	**93**
8	DCE	Fe(NO_3_)_3_	reflux	6	nr
9	THF	Fe(NO_3_)_3_	reflux	6	nr
10	CH_3_CN	K_3_(Fe)(CN)_6_	reflux	6	nr

^a^Reaction conditions: azidochalcone **1** (1 equiv), potassium thiocyanate **2** (3 equiv), Fe(III) (0.5 equiv), solvent (2 mL). ^b^Isolated yield after column chromatography.

This reaction has led to the exclusive formation of 4,5-disubstituted 2-aminothiazole **4d**. The catalytic activities of different Fe(III) salts and the solvents were screened in the reaction. When ferric chloride was employed in DCE or toluene, the expected product was not obtained (Table 2, entries 2 and 3). The reaction failed to proceed in acetonitrile at room temperature also (Table 2, entry 4). As a further variation, we examined Fe_2_O_3_ as catalyst in acetonitrile which resulted only in 10% conversion (Table 2, entry 5). Further screening was performed with ferric nitrate and the 2-aminothiazole was obtained in 60% yield (Table 2, entry 6). The product was obtained in excellent yield when the reaction mixture was heated for 6 h (Table 2, entry 7). Potassium ferricyanide has also been proved ineffective (Table 2, entry 10).

**Scheme 8:**
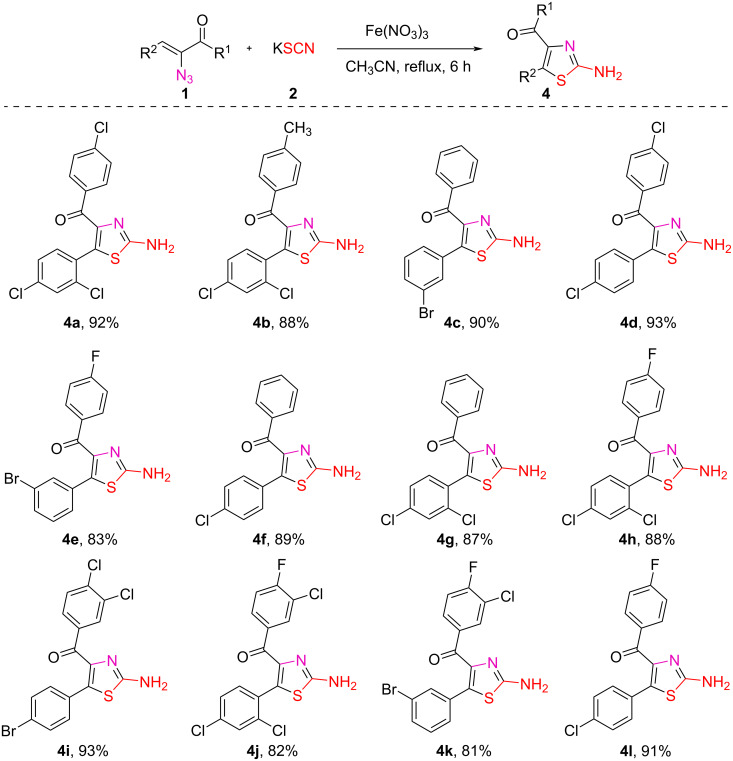
Reaction of vinyl azide **1** and **3** with ferric nitrate. Reactions were carried out at reflux temperature, using **1** (1 mmol), **2** (3 mmol), ferric nitrate (0.5 mmol) in acetonitrile (2 mL) for 6 h. Yields refer to the pure products after column chromatography.

Using the optimized experimental conditions, the Fe(III)-mediated formation of 4,5-disubstituted 2-aminothiazoles **4** was examined for the substrate scope (Scheme 8).

**Figure 4:**
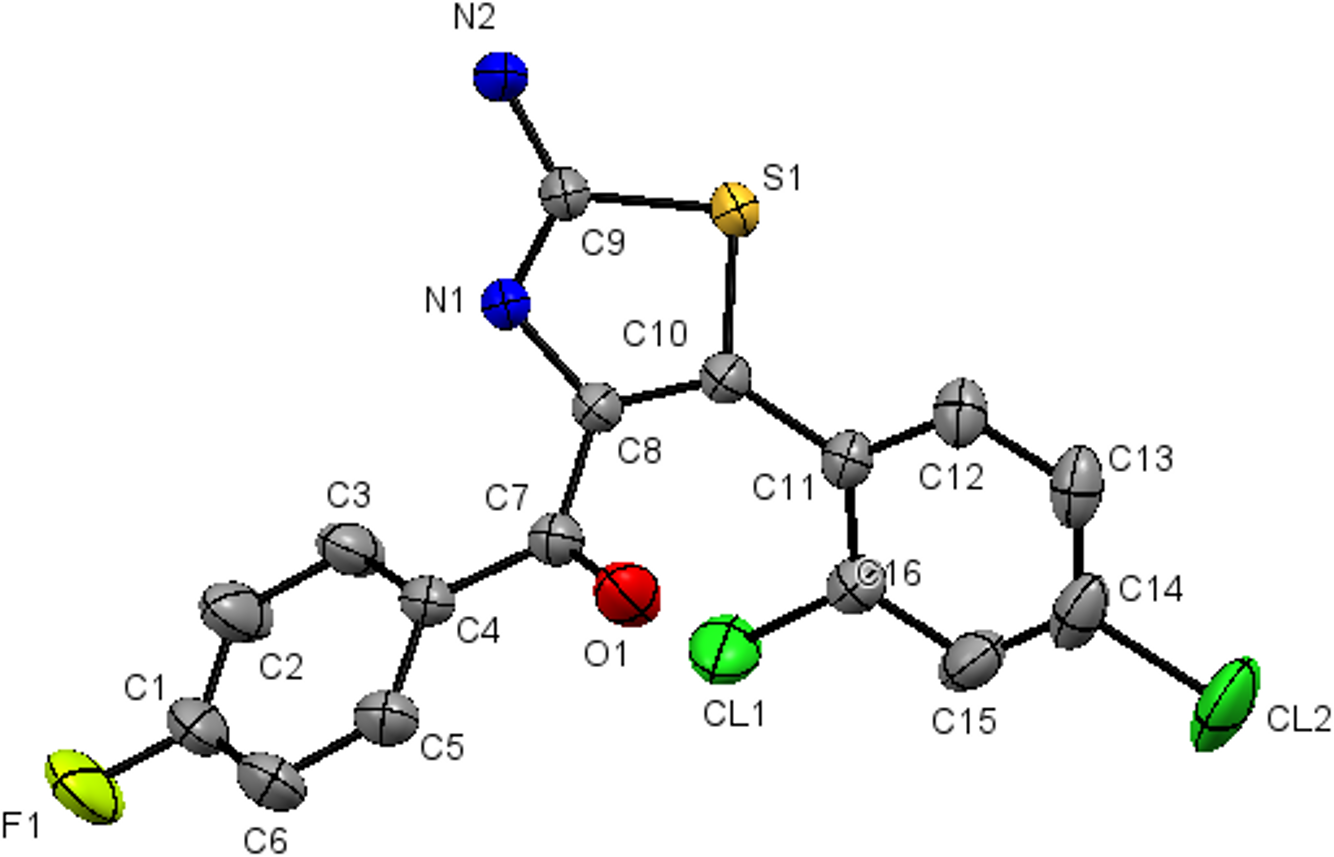
X-ray crystal structure of **4h**.

All the synthesized compounds **4a–l** were confirmed by 1D and 2D NMR, IR spectroscopy and HRMS techniques. Additional evidence of the structures of these compounds was obtained based on the single-crystal X-ray analysis of **4h** [71] (Figure 4).

The nitrogen end of the thiocyanate attacks the azirine to form the oxazole ring and the sulfur end of the thiocyanate involves in the reaction resulting in the 2-aminothiazole ring. The mechanism for the formation of **4** may be similar to that suggested earlier [73].

## Conclusion

In conclusion, we have demonstrated selective routes for the synthesis of highly substituted oxazoles and 2-aminothiazoles from α-azidochalcones and potassium thiocyanate employing potassium persulfate and ferric nitrate, respectively. This new route gains a streamlined workup and the elimination of air-sensitive techniques to afford the product in good yield in a greener medium over a short time frame. The current method involves a broad substrate scope, excellent functional group tolerance and leaves the active site for further synthetic transformation. The overall strategy allows the generation of new C–N and C–O bonds in one-pot.

## Experimental

**General considerations**: The melting points reported in the work are uncorrected. Unless stated otherwise, solvents and chemicals were obtained from commercial sources and used without further purification. Infrared spectra were recorded on a Perkin Elmer instrument with neat sample and only major peaks are reported in cm^−1^. The ^1^H and ^13^C NMR spectra of the new compounds were measured at 400 MHz and 100 MHz, respectively, using Bruker and JEOL NMR instruments in DMSO-*d*_6_. Chemical shifts are reported in parts per million (δ), coupling constants (*J* values) are reported in Hertz (Hz) relative to tetramethylsilane. Spin multiplicities are indicated by the following symbols: s (singlet), d (doublet), t (triplet), m (multiplet), dd (doublet of doublets), td (triplet of doublets), ddd (doublet of doublets of doublets), bs (broad singlet). Mass spectra were measured with Micromass Q-Time of flight (HRESIMS).

**General procedure for the preparation of 3:** To a solution of azidochalcone **1** (1 mmol) in dry acetonitrile (2 mL) were added potassium thiocyanate **2** (3 mmol) and potassium persulfate (0.5 mmol). The mixture was stirred magnetically at reflux temperature for 6 hours under nitrogen atmosphere. After completion of the reaction (monitored by TLC), the solid that separated was filtered, washed with water and acetonitrile and recrystallized with cold diethyl ether to obtain pure yellow product **3**.

**General procedure for the preparation of 4:** To a solution of azidochalcone **1** (1 mmol) in dry acetonitrile (2 mL) were added potassium thiocyanate **2** (3 mmol) and ferric nitrate (0.5 mmol). The reaction mixture was stirred magnetically at reflux for 6 h. After completion of the reaction (monitored by TLC), the product was diluted with water, extracted with ethyl acetate (15 mL) and purified by column chromatography (100–200 mesh silica gel) using ethyl acetate/petroleum ether mixture to afford product **4**.

## Supporting Information

File 1 Full experimental details, compound characterisation, and copies of NMR spectra.
