# Supplementary material for: Access to highly substituted oxazoles by the reaction of α-azidochalcone with potassium thiocyanate
Source: Beilstein J Org Chem. 2020 Aug 31;16:2108–18. doi: 10.3762/bjoc.16.178 (PMC7476590; doi:10.3762/bjoc.16.178)
Supplement: File 1 — Full experimental details, compound characterisation, and copies of NMR spectra. [file Beilstein_J_Org_Chem-16-2108-s001.pdf]

BEILSTEIN JOURNAL OF ORGANIC CHEMISTRY

Supporting Information

for

Access to highly substituted oxazoles by the reaction of α-azidochalcone with potassium thiocyanate

Mysore Bhyrappa Harisha, Pandi Dhanalakshmi, Rajendran Suresh, Raju Ranjith Kumar and Shanmugam Muthusubramanian

Beilstein J. Org. Chem. **2020,** *16,* 2108–2118. doi:10.3762/bjoc.16.178

Full experimental details, compound characterisation, and copies of NMR spectra

License and Terms: This is a supporting information file under the terms of the Creative Commons Attribution License (https://creativecommons.org/licenses/by/4.0). Please note that the reuse, redistribution and reproduction in particular requires that the authors and source are credited.
The license is subject to the *Beilstein Journal of Organic Chemistry* terms and conditions: (https://www.beilstein-journals.org/bjoc)

Contents

I General consideration S2

II Characterization data of **3** S2

III Derivatives of **3** S9

IV Characterization data of **4** S11

V Copies of NMR spectra of **3a–u** S15

VI Copies of NMR spectra of **5, 6** and **7** S38

VII Copies of NMR spectra of **4a–l** S41

VIII Reference S55

I General considerations

The melting points reported in the work are uncorrected. Unless stated otherwise, solvents and chemicals were obtained from commercial sources and used without further purification. Infrared spectra were recorded on a Perkin Elmer instrument with neat sample and only major peaks are reported in cm^{-1}. The ^{1}H and ^{13}C NMR spectra of the new compounds were measured at 400 MHz and 100 MHz, respectively, using Bruker and JEOL NMR instruments in DMSO-d_6. Chemical shifts are reported in parts per million (δ), coupling constants (*J* values) are reported in Hertz (Hz) relative to tetramethylsilane. Spin multiplicities are indicated by the following symbols: s (singlet), d (doublet), t (triplet), m (multiplet), dd (doublet of doublets), td (triplet of doublets), ddd (doublet of doublets of doublets), bs (broad singlet). Mass spectra were measured with Micromass Q-Time of flight (ESI–HRMS)

General procedure for the preparation of 3

To a solution of azidochalcone **1** (1 mmol) in dry acetonitrile (2 mL) were added potassium thiocyanate **2** (3 mmol) and potassium persulfate (0.5 mmol). The mixture was stirred magnetically at reflux temperature for 6 hours under nitrogen atmosphere. After completion of the reaction (monitored by TLC), the solid that separated was filtered, washed with water and acetonitrile and recrystallized with cold diethyl ether to obtain pure yellow product **3**.

II Characterization data of 3

(*E*)-4-(3-Bromobenzylideneamino)-5-(4-fluorophenyl)oxazole-2-thiol (3a): Isolated yield 0.196 g (90%); yellow solid; M. pt. 251-253^{0}C; IR (neat, cm^{-1}) 3450, 2914, 2335, 1643, 653; ^{1}H NMR (400 MHz, DMSO-d_6): δ_H: 14.03 (s, 1H), 8.64 (s, 1H), 8.00 - 7.95 (m, 3H), 7.87 (d, *J* = 7.6 Hz, 1H), 7.77 (d, *J* = 8.0 Hz, 1H), 7.53 (t, *J* = 8.0 Hz, 1H), 7.39 (t, *J* = 8.8 Hz, 2H); ^{13}C NMR (100 MHz, DMSO-d_6) δ_C: 176.6, 162.5, 158.7, 139.9, 138.1, 135.2, 132.4, 131.8, 131.2, 128.2, 127.9, 127.8, 122.9, 116.7; LC-MS calcd. m/z 377, found 378 [(M+1)]$^{+}$. HRMS (ESI-TOF): (*m/z*) calcd for $C_{16}H_{10}BrFN_2OSH$ [M + H]$^{+}$: 376.9759; Found: 376.9756.

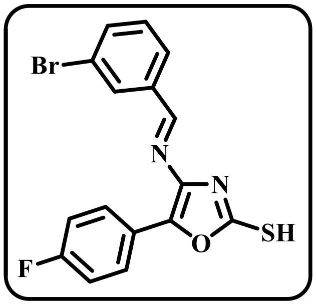

(*E*)-4-(4-Chlorobenzylideneamino)-5-(2,4-dichlorophenyl)oxazole-2-thiol (3b): Isolated yield 0.195 g (89%); yellow solid; M. pt. 228-230^{0}C; IR (neat, cm^{-1}) 3070, 2351, 1483, 1204, 663; ^{1}H NMR (400 MHz, DMSO-d_6): δ_H: 14.14 (s, 1H), 8.01 (s, 1H), 7.99 - 7.92 (m, 4H), 7.73 - 7.65 (m, 3H); ^{13}C NMR (100 MHz, DMSO-d_6) δ_C: 177.3, 160.1, 137.9, 137.5, 135.7, 135.1, 134.5, 133.8, 133.4, 130.8, 130.5, 129.8, 128.2, 124.4; LC-MS calcd. m/z 383, found 384 [(M+1)]$^+$. HRMS (ESI-TOF): (*m/z*) calcd for $C_{16}H_9Cl_3N_2OSH$ [M+H]$^+$: 382.9579; Found: 382.9578.

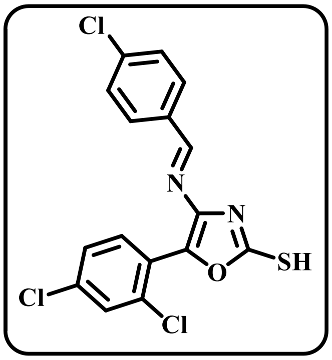

(*E*)-4-(3-bromobenzylideneamino)-5-(4-chloro-3-fluorophenyl)oxazole-2-thiol (3c): Isolated yield 0.199 g (92%); yellow solid; M. pt. 259-261^{0}C; IR (neat, cm^{-1}) 3066, 2341, 1483, 1186, 1101; ^{1}H NMR (400 MHz, DMSO-d_6): δ_H: 14.14 (s, 1H), 8.69 (s, 1H), 8.08 (d, *J* = 6.4 Hz, 1H), 8.00 (s, 1H), 7.92 (bs, 1H), 7.85 (d, *J* = 7.6 Hz, 1H), 7.79 (d, *J* = 7.6 Hz, 1H), 7.61 - 7.52 (m, 2H); ^{13}C NMR (100 MHz, DMSO-d_6) δ_C: 176.2, 158.9, 156.9, 137.8, 134.8, 132.9, 131.3, 130.5, 127.9, 126.7, 125.6, 124.3, 122.4, 120.4, 120.3, 117.9; LC-MS calcd. m/z 411, found 412. HRMS (ESI-TOF): m/z calcd for $C_{16}H_9BrClFN2OSH$ [M+H]$^+$: 410.9370, Found: 410.9366.

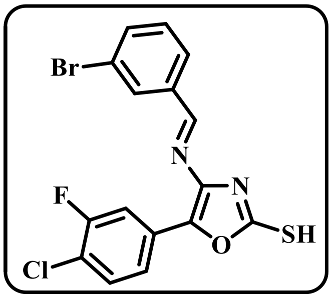

(*E*)-4-(2,4-Dichlorobenzylideneamino)-5-phenyloxazole-2-thiol (3d): Isolated yield 0.20 g (93%); orange solid; M. pt. 251-253^{0}C; IR (neat, cm^{-1}) 3076, 2335, 1516, 1188, 1062; ^{1}H NMR (400 MHz, DMSO-d_6): δ_H: 14.30 (s, 1H), 8.95 (s, 1H), 8.18 (d, *J* = 8.4 Hz, 1H), 7.98 (d, *J* = 7.2 Hz, 2H), 7.77 (s, 1H), 7.59 (d, *J* = 8.4 Hz, 1H), 7.54 (t, *J* = 7.2 Hz, 2H), 7.43 (t, *J* = 7.2 Hz, 1H); ^{13}C NMR (100 MHz, DMSO-d_6) δ_C: 176.8, 154.4, 141.3, 137.6, 136.6, 133.1, 132.2, 130.3, 129.8, 129.5, 129.4, 128.7, 126.9, 125.7; LC-MS calcd. m/z 349, found 350 [(M+1)]$^+$. HRMS (ESI-TOF): m/z calcd for $C_{16}H_{10}Cl_2N_2OSH$ [M+H]$^+$: 348.9969, Found: 348.9966.

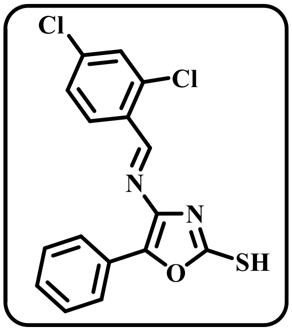

(*E*)-4-(4-Bromobenzylideneamino)-5-(3,4-dichlorophenyl)oxazole-2-thiol (3e): Isolated yield 0.191 g (89%); yellow solid; M. pt. 263-265^{0}C; IR (neat, cm^{-1}) 3089, 2324, 1483, 1188, 1091; ^{1}H NMR (400 MHz, DMSO-d_6): δ_H: 14.13 (s, 1H), 8.73 (s, 1H), 8.41 (d, *J* = 1.8 Hz,

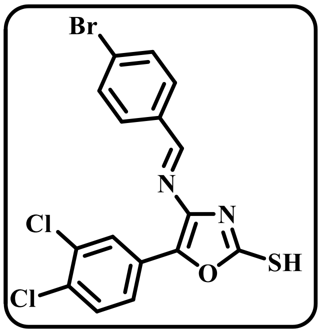

1H), 8.14 (dd, J = 1.9 Hz, 8.4 Hz, 1H), 7.85 (d, J = 8.4 Hz, 1H), 7.82 - 7.80 (m, 4H); ^{13}C NMR (100 MHz, DMSO-d_6) δ_C: 176.3, 160.0, 137.2, 134.3, 134.0, 132.5,* 132.3, 130.4, 130.0, 129.3,* 126.4, 123.5; LC-MS calcd. m/z 428, found 429 [(M+1)]$^+$. [*Two signals merged here]. HRMS (ESI-TOF): m/z calcd for $C_{16}H_9BrCl_2N_2OSH$ [M+H]$^+$: 426.9074, found: 426.9073.

(*E*)-5-(4-Chlorophenyl)-4-(2,4-dichlorobenzylideneamino)oxazole-2-thiol (3f): Isolated yield 0.20 g (93%); yellow solid; M. pt. 246-248^{0}C; IR (neat, cm^{-1}) 3072, 2358, 1498, 1180, 1089; ^{1}H NMR (400 MHz, DMSO-d_6): δ_H: 14.36 (s, 1H), 8.96 (s, 1H), 8.20 (d, J = 8.5 Hz, 1H), 7.98 - 7.96 (m, 2H), 7.79 (d, J = 2.0 Hz, 1H),7.58 - 7.56 (m, 3H); ^{13}C NMR (100 MHz, DMSO-d_6) δ_C: 176.9, 155.0, 140.3, 137.8, 136.7, 133.9, 133.5, 132.1, 130.4, 130.0, 129.7, 128.7, 127.3, 125.7; LC-MS calcd.m/z 383, found 384 [(M+1)]$^+$. HRMS (ESI-TOF): m/z calcd for $C_{16}H_9Cl_3N_2OSH$ [M + H]$^+$: 382.9579, Found: 382.9576.

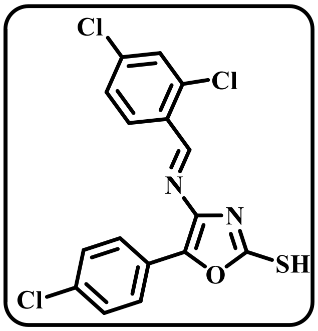

(*E*)-4-(2,4-Dichlorobenzylideneamino)-5-(4-fluorophenyl)oxazole-2-thiol (3g): Isolated yield 0.197 g (90%); orange solid; M. pt. 243-245^{0}C; IR (neat, cm^{-1}) 3099, 2341, 1483, 1228, 1157; ^{1}H NMR (400 MHz, DMSO-d_6): δ_H: 14.29 (s, 1H), 8.95 (s, 1H), 8.18 (d, J = 8.5 Hz, 1H), 8.02 - 7.99 (m, 2H), 7.77 (d, J = 1.9 Hz, 1H), 7.58 (dd, J = 1.6 Hz, 8.5 Hz, 1H), 7.38 (t, J = 8.8 Hz, 2H); ^{13}C NMR (100 MHz, DMSO-d_6) δ_C: 176.7, 162.6, 154.5, 140.6, 137.6, 136.6, 132.8, 132.2, 130.1, 128.7, 128.1, 128.0, 123.6, 116.7; LC-MS calcd. m/z 366, found 367 [(M+1)]$^+$. HRMS (ESI-TOF): m/z calcd for $C_{16}H_9Cl_2FN_2OSH$ [M+H]$^+$: 366.9875, found: 366.9872.

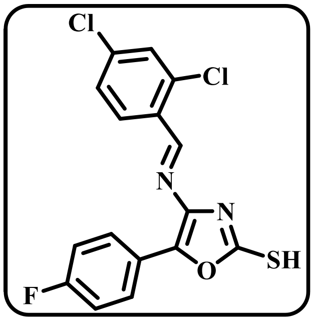

(*E*)-5-(3-Chloro-4-fluorophenyl)-4-(2,4-dichlorobenzylideneamino)oxazole-2-thiol (3h): Isolated yield 0.199 g (92%); yellow solid; M. pt. 255-257^{0}C; IR (neat, cm^{-1}) 3064, 2956, 2351, 1192, 1184; ^{1}H NMR (400 MHz, DMSO-d_6): δ_H: 14.46 (s, 1H), 8.98 (s, 1H), 8.14 (d, J = 8.5 Hz, 1H), 8.05 (dd, J = 2.2 Hz, 7.2 Hz, 1H), 7.99 - 7.95 (m, 1H), 7.81 (d, J = 2.0 Hz, 1H), 7.62 (dd, J = 1.7 Hz, 8.5 Hz, 1H), 7.59 (t, J = 9 Hz, 1H); ^{13}C NMR (100 MHz, DMSO-d_6) δ_C: 176.3, 156.6, 152.6, 138.5, 137.4, 136.1, 133.1, 131.5, 129.5, 128.2, 126.8, 125.9, 124.2, 120.5, 120.3, 117.9; LC-MS

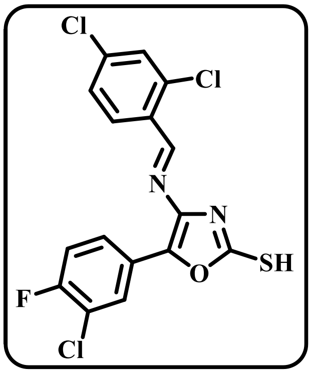

calcd. m/z 401, found 402 [(M+1)]$^+$. HRMS (ESI-TOF): m/z calcd for $C_{16}H_8Cl_3FN_2OSH$ [M+H]$^+$: 400.9485, Found: 400.9488.

(*E*)-4-(4-Chlorobenzylideneamino)-5-phenyloxazole-2-thiol (3i): Isolated yield 0.22 g (97%); yellow solid; M. pt. 254-256^{0}C; IR (neat, cm^{-1}) 3082, 2941, 2345, 1510, 1182; ^{1}H NMR (400 MHz, DMSO-d_6): δ_H: 13.98 (s, 1H), 8.73 (s, 1H), 7.99 (d, J = 8.5 Hz, 2H), 7.91 (d, J = 8.5 Hz, 2H), 7.64 (d, J = 8.5 Hz, 2H), 7.54 (t, J = 7.6 Hz, 2H), 7.41 (t, J = 7.4 Hz, 1H); ^{13}C NMR (100 MHz, DMSO-d_6) δ_C: 176.7, 158.8, 140.2, 137.2, 134.8, 131.6, 130.8, 129.9, 129.5, 129.1, 127.2, 125.4; LC-MS calcd. m/z 314, found 315 [(M+1)]$^+$. HRMS (ESI-TOF): m/z calcd for $C_{16}H_{11}ClN_2OSH$ [M+H]$^+$: 315.0359, found: 315.0362.

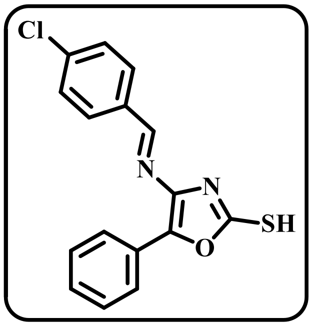

(*E*)-4-(3-Bromobenzylideneamino)-5-phenyloxazole-2-thiol (3j): Isolated yield 0.199 g (91%); yellow solid; M. pt. 256-258^{0}C; IR (neat, cm^{-1}) 3097, 2926, 2345, 1506, 1188, 1062; ^{1}H NMR (400 MHz, DMSO-d_6): δ_H: 14.08 (s, 1H), 8.66 (s, 1H), 7.96 - 7.94 (m, 3H), 7.88 (d, J = 7.6 Hz, 1H), 7.78 - 7.75 (m, 1H), 7.54 - 7.50 (m, 3H), 7.42 (d, J = 7.2 Hz, 1H); ^{13}C NMR (100 MHz, DMSO-d_6) δ_C: 176.7, 158.6, 140.6, 138.2, 135.1, 132.8, 131.8, 131.1, 129.5, 129.3, 128.2, 127.0, 125.5, 122.9; LC-MS calcd.m/z 359, found 360 [(M+1)]$^+$. HRMS (ESI-TOF): m/z calcd for $C_{16}H_{11}BrN_2OSH$ [M+H]$^+$: 358.9854, found: 358.9865.

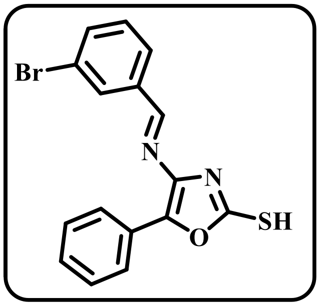

(*E*)-4-(4-Chlorobenzylideneamino)-5-(4-chlorophenyl)oxazole-2-thiol (3k): Isolated yield 0.21 g (95%); yellow solid; M. pt. 228-230^{0}C; IR (neat, cm^{-1}) 3062, 2925, 1504, 1191, 1086; ^{1}H NMR (400 MHz, DMSO-d_6): δ_H: 14.08 (s, 1H), 8.71 (s, 1H), 7.98 (d, J = 8.7 Hz, 2H), 7.91 (d, J = 8.5 Hz, 2H), 7.64 (d, J = 8.4 Hz, 2H), 7.59 (d, J = 8.6 Hz, 2H); ^{13}C NMR (100 MHz, DMSO-d_6) δ_C: 176.7, 159.5, 139.2, 137.4, 134.7, 133.6, 133.4, 130.9, 129.9, 129.7, 127.0, 125.9; LC-MS calcd.m/z 349, found 350 [(M+1)]$^+$. HRMS (ESI-TOF): m/z calcd for $C_{16}H_{10}Cl_2N_2OSH$ [M+H]+: 348.9969, found: 348.9969.

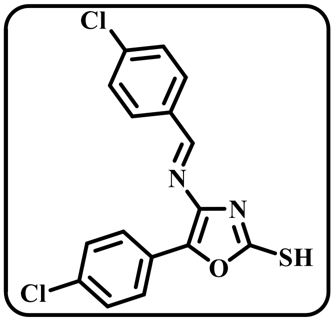

(*E*)-4-(2,4-Dichlorobenzylideneamino)-5-*p*-tolyloxazole-2-thiol (3l): Isolated yield 0.19 g (87%); orange solid; M. pt. 252-254^{0}C; IR (neat, cm^{-1}) 2131, 1651, 1388, 1282, 1130; ^{1}H NMR (400 MHz, DMSO-d_6): δ_H: 14.28 (s, 1H), 8.96 (s, 1H), 8.19 - 7.88 (m, 3H), 7.80 - 7.35 (m, 4H), 2.50 (s, 3H); ^{13}C NMR (100 MHz, DMSO-d_6)* δ_C: 153.8, 146.8, 139.3, 136.4, 132.3, 130.3, 130.2, 129.8, 128.7, 125.7, 124.2, 21.5; LC-MS calcd. m/z 363, found 364 [(M+1)]$^+$. HRMS (ESI-TOF): m/z calcd for $C_{17}H_{12}Cl_2N_2OSH$ [M+H]$^+$: 363.0125, found: 363.0129. [*Not all the carbon signals got picked up]

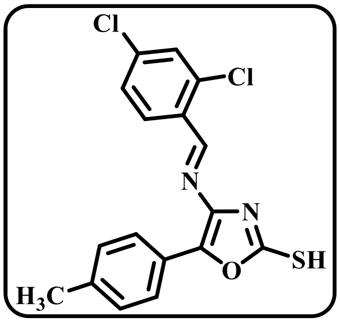

(*E*)-4-(4-Chlorobenzylideneamino)-5-(4-fluorophenyl)oxazole-2-thiol (3m): Isolated yield 0.21 g (93%); yellow solid; M. pt. 271-273^{0}C; IR (neat, cm^{-1}) 3061, 2934, 1607, 1515, 1082; ^{1}H NMR (400 MHz, DMSO-d_6): δ_H: 14.02 (s, 1H), 8.69 (s, 1H), 8.01 (t, J = 8.5 Hz, 2H), 7.89 (d, J = 8.4 Hz, 2H), 7.62 (d, J = 8.3 Hz, 2H), 7.38 (t, J = 8.8 Hz, 2H); ^{13}C NMR (100 MHz, DMSO-d_6) δ_C: 176.6, 162.5, 159.0, 139.6, 137.3, 134.7, 132.6, 130.8, 129.8, 127.8, 123.7, 116.7; LC-MS calcd. m/z 332, found 333 [(M+1)]$^+$. HRMS (ESI-TOF): m/z calcd for $C_{16}H_{10}ClFN_2OSH$ [M+H]$^+$: 333.0264, found: 333.0268.

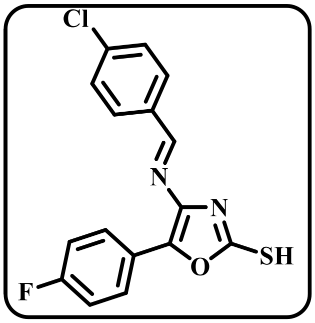

(*E*)-4-(3-Bromobenzylideneamino)-5-(2,4-dichlorophenyl)oxazole-2-thiol (3n): Isolated yield 0.19 g (90%); yellow solid; M. pt. 240-242^{0}C; IR (neat, cm^{-1}) 2920, 1737, 1577, 1450, 1263; ^{1}H NMR (400 MHz, DMSO-d_6): δ_H: 14.20 (s, 1H), 8.68 (s, 1H), 7.90 (s, 1H), 7.83 (d, J = 1.8 Hz, 1H), 7.78 - 7.74 (m, 3H), 7.62 (dd, J = 1.9 Hz, 8.4 Hz, 1H), 7.49 (t, J = 7.8 Hz, 1H); ^{13}C NMR (100 MHz, DMSO-d_6) δ_C: 177.3, 159.9, 138.2, 137.9, 135.8, 135.4, 135.0, 133.9, 133.5, 131.8, 130.9, 130.5, 128.5, 128.2, 124.3, 122.8; LC-MS calcd. m/z 426, found 427 [(M+1)]$^+$. HRMS (ESI-TOF): m/z calcd for $C_{16}H_9BrCl_2N_2OSH$ [M+H]$^+$: 426.9074, found: 426.9072.

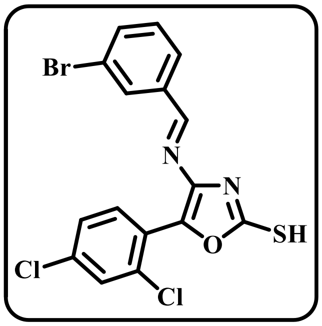

(*E*)-5-(2,4-Dichlorophenyl)-4-(4-methoxybenzylideneamino)oxazole-2-thiol (3o): Isolated yield 0.19 g (88%); pale yellow solid; M. pt. 222-224^{0}C; IR (neat, cm^{-1}) 2916, 1585, 1496, 1182, 1083; ^{1}H NMR (400 MHz, DMSO-*d*$_6$): δ_H: 14.06 (s, 1H), 8.66 (s, 1H), 7.81 - 7.75 (m, 4H), 7.61 (dd, *J* = 2 Hz, 8.4 Hz, 1H), 7.08 (d, *J* = 8.8 Hz, 2H), 3.81 (s, 3H); ^{13}C NMR (100 MHz, DMSO-*d*$_6$) δ_C: 177.1, 163.3, 161.0, 136.5, 135.6, 135.3, 133.6, 133.2, 131.3, 130.5, 128.5, 128.1, 124.7, 115.1, 56.0; LC-MS calcd. m/z 379, found 380 [(M+1)]$^+$. HRMS (ESI-TOF): m/z calcd for $C_{17}H_{12}Cl_2N_2OSH$ [M+H]$^+$: 379.0075, found: 379.0075.

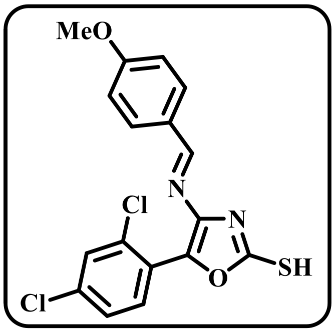

(*E*)-4-(3-Chlorobenzylideneamino)-5-(4-fluorophenyl)oxazole-2-thiol (3p): Isolated yield 0.20 g (92%); pale yellow solid; M. pt. 264^{0}C; IR (neat, cm^{-1}) 2926, 1736, 1596, 1500, 1164; ^{1}H NMR (400 MHz, DMSO-*d*$_6$): δ_H: 14.03 (s, 1H), 8.73 (s, 1H), 8.06 - 8.02 (m, 2H), 7.90 (dd, *J* = 1.5 Hz, 7.4 Hz, 2H), 7.70 - 7.64 (m, 2H), 7.44 (t, *J* = 8.9 Hz, 2H); ^{13}C NMR (100 MHz, DMSO-*d*$_6$) δ_C: 176.6, 162.5, 158.9, 139.9, 137.9, 134.5, 131.9, 128.2, 128.0, 127.9, 127.8, 123.6, 123.6, 116.8; LC-MS calcd. m/z 332, found 333 [(M+1)]$^+$. HRMS (ESI-TOF): m/z calcd for $C_{16}H_{10}ClFN_2OSK$ [M+K]$^+$: 370.9823, found: 370.9823.

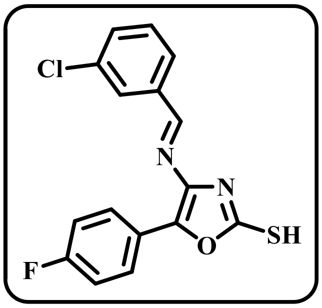

(*E*)-4-(5-Bromo-2-fluorobenzylideneamino)-5-(4-fluorophenyl)oxazole-2-thiol (3q): Isolated yield 0.19 g (90%); pale yellow solid; M. pt. 262^{0}C; IR (neat, cm^{-1}) 2930, 2069, 1738, 1450, 1192; ^{1}H NMR (400 MHz, DMSO-*d*$_6$): δ_H: 14.11 (s, 1H), 8.84 (s, 1H), 8.08 - 8.07 (m, 1H), 7.99 - 7.95 (m, 2H), 7.78 - 7.76 (m, 1H), 7.38 - 7.34 (m, 3H); ^{13}C NMR (100 MHz, DMSO-*d*$_6$) δ_C: 161.6, 160.7, 150.5, 136.0, 135.9, 128.9, 127.1, 124.7, 124.6, 122.7, 122.4, 118.3, 116.5, 115.7; LC-MS calcd. m/z 395, found 396 [(M+1)]$^+$. HRMS (ESI-TOF): m/z calcd for $C_{16}H_9BrF_2N_2OSH$ [M+H]$^+$: 394.9665, found: 394.9660.

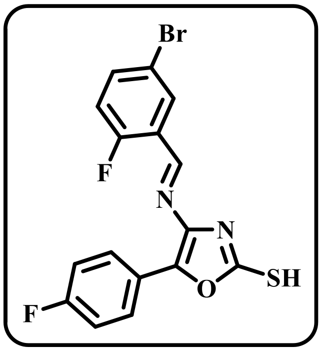

(*E*)-4-(3-Fluorobenzylideneamino)-5-(4-fluorophenyl)oxazole-2-thiol (3r): Isolated yield 0.21 g (93%); pale yellow solid; M. pt. 251-253^{0}C; IR (neat, cm^{-1}) 2924, 1736, 1502, 1081; ^{1}H NMR (400 MHz, DMSO-d_6): δ_H: 14.04 (s, 1H), 8.71 (s, 1H), 8.03 (t, J = 5.8 Hz, 2H), 7.70 - 7.59 (m, 3H), 7.46 - 7.37 (m, 3H); ^{13}C NMR (100 MHz, DMSO-d_6) δ_C: 176.6, 163.0, 162.5, 159.0, 139.8, 138.3, 132.4, 131.8, 127.9, 126.1, 123.6, 119.6, 116.7, 114.4; LC-MS calcd. m/z 316, found 317 [(M+1)]$^+$. HRMS (ESI-TOF): m/z calcd for $C_{16}H_{10}F_2N_2OSH$ [M+H]$^+$: 317.0560, found: 317.0557.

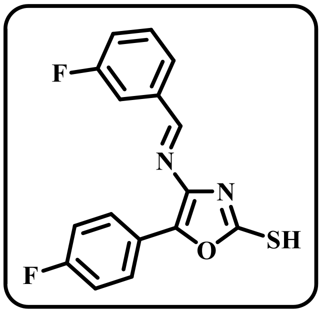

(*E*)-5-(4-Fluorophenyl)-4-(3-nitrobenzylideneamino)oxazole-2-thiol (3s): Isolated yield 0.19 g (89%); pale yellow solid; M. pt. 257-258^{0}C; IR (neat, cm^{-1}) 3433, 3039, 2065, 1513, 1391; ^{1}H NMR (400 MHz, DMSO-d_6): δ_H: 14.10 (s, 1H), 8.81 (s, 1H), 8.53 (s, 1H), 8.40 (d, J = 7.9 Hz, 1H), 8.34 (d, J = 7.6 Hz, 1H), 8.03 (t, J = 5.6 Hz, 2H), 7.87 (t, J = 7.9 Hz, 1H), 7.41 (t, J = 8.7 Hz, 2H); ^{13}C NMR (100 MHz, DMSO-d_6) δ_C: 176.2, 162.1, 157.6, 148.3, 139.8, 136.8, 134.1, 131.8, 130.8, 127.5, 126.2, 123.0, 122.8, 116.2; LC-MS calcd. m/z 343, found 344 [(M+1)]$^+$. HRMS (ESI-TOF): m/z calcd for $C_{16}H_9FN_3O_3SH$ [M+H]$^+$: 344.0505, found: 344.0503.

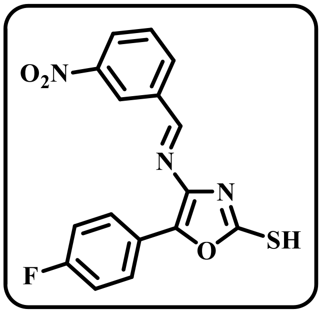

(*E*)-4-(2,4-Dichlorobenzylideneamino)-5-(3,4-dimethylphenyl)oxazole-2-thiol (3t): Isolated yield 0.18 g (85%); pale yellow solid; M. pt. 242^{0}C; IR (neat, cm^{-1}) 3058, 2940, 2344, 1738, 1514, 1192; ^{1}H NMR (400 MHz, DMSO-d_6): δ_H: 14.17 (s, 1H), 8.89 (s, 1H), 8.12 (d, J = 8.4 Hz, 1H), 7.74 - 7.69 (m, 3H), 7.59 (d, J = 8.4 Hz, 1H), 7.26 (d, J = 8.0 Hz, 1H), 2.40 (s, 3H), 2.06 (s, 3H); ^{13}C NMR* (100 MHz, DMSO-d_6) δ_C:137.5, 130.7, 130.3, 129.7, 128.8, 126.5, 123.5, 120.1, 20.1, 19. 9; LC-MS calcd. m/z 376, found 377 [(M+1)]$^+$. HRMS (ESI-TOF): m/z calcd for $C_{18}H_{14}Cl_2N_2OSH$ [M+H]$^+$: 377.0282, found: 377.0279. [*Not all carbon signals got picked up].

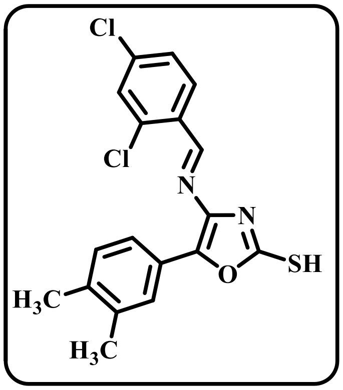

(*E*)-4-(Benzo[*d*][1,3]dioxol-4-ylmethyleneamino)-5-(4-fluorophenyl)oxazole-2-thiol (3u): Isolated yield 0.20 g (92%); pale yellow solid; M.pt. 245-246^{0}C; IR (neat, cm^{-1}) 2336, 1513, 1481, 1374, 1173, 819; ^{1}H NMR (400 MHz, DMSO-d_6): δ_H: 13.93 (s, 1H), 8.60 (s, 1H), 8.01 - 7.98 (m, 2H), 7.48 (d, *J* = 1.4 Hz, 1H), 7.39 (t, *J* = 8.9 Hz, 2H), 7.32 (dd, *J* = 1.5 Hz, 7.9 Hz, 1H), 7.10 (d, *J* = 7.9 Hz, 1H), 6.17 (s, 2H); ^{13}C NMR (100 MHz, DMSO-d_6) δ_C: 176.5, 162.2, 159.7, 151.6, 148.9, 138.5, 132.9, 130.7, 127.5, 127.3, 123.9, 116.7, 109.2, 106.1, 102.6; LC-MS calcd. m/z 342, found 343 [(M+1)]$^+$. HRMS (ESI-TOF): m/z calcd for $C_{17}H_{11}FN_2O_3SH$ [M+H]$^+$: 343.0553, found: 343.0551.

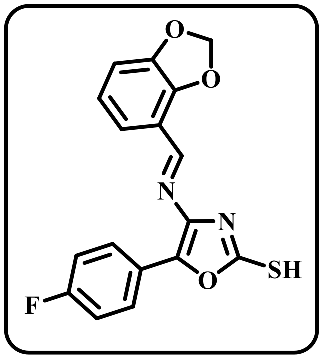

III Derivatives of 3

Acetylated derivative of 3d

(*E*)-S-4-(2,4-Dichlorobenzylideneamino)-5-phenyloxazol-2-yl ethanethioate (5): To a stirred suspension of sodium hydride (60% suspension in oil (0.028 g, 0.70 mmol) in dry THF (2 ml) at 0 - 5 ^{0}C, **3d** (0.2 g, 0.64 mmol) was added and the reaction mixture was allowed to stir at room temperature for 15 minutes, followed by the addition of acetyl chloride (0.12 g, 0.70 mmol). The mixture was further stirred for 30 minutes at room temperature. After completion of the reaction (monitored by TLC), the mixture was diluted with ice water and extracted with ethyl acetate. The combined organic phase was washed with water, brine, dried over anhydrous Na_2SO_4 filtered and concentrated in vacuum to give crude **5**. The crude was purified by column chromatography on 100-200 mesh silica gel using pet ether /ethyl acetate as eluent. Pale yellow solid; 0.17 g (77%), M.pt. 250^{0}C (Decomposes). ^{1}H NMR (400 MHz, DMSO-d_6): δ_H: 9.15 (s, 1H), 8.23 (d, *J* = 6.8 Hz, 1H), 7.95 (dd, *J* = 0.92, 6.8 Hz, 2H), 7.74 (d, *J* = 1.6 Hz, 1H), 7.57 - 7.59 (m, 1H), 7.41 (t, *J* = 6.1 Hz, 2H), 7.21 - 7.18 (m, 1H), 1.65 (s, 3H); ^{13}C NMR (100 MHz, DMSO-d_6) δ_C: 176.7, 149.3, 143.3, 139.6, 134.7, 134.6, 132.1, 129.0, 128.7, 128.3, 128.0,* 127.5, 125.5, 123.7, 24.2 [*Two carbons merge here]. HRMS (ESI-TOF): m/z calcd for $C_{18}H_{12}Cl_2N_2O_2SH$ [M+H]$^+$: 391.0075, found: 391.0079.

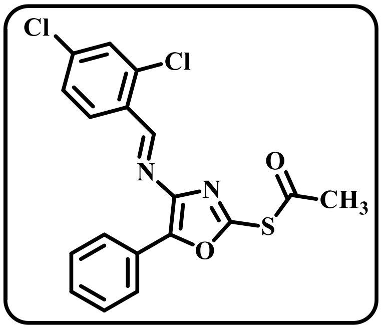

Procedure for the methylation of 3m

(*E*)-*N*-(4-Chlorobenzylidene)-5-(4-fluorophenyl)-2-(methylthio)oxazol-4-amine (6): To a stirred suspension of sodium hydride (60% suspension in oil (0.028 g, 0.70 mmol) in dry THF (2 ml) at 0–5 °C, **3m** (0.2 g, 0.64 mmol) was added and the reaction mixture was allowed to stir at room temperature for 15 minutes, followed by the addition of methyl iodide (0.1 g, 0.70 mmol). The mixture was further stirred for 2 hours at room temperature. After completion of the reaction (monitored by TLC), the mixture was diluted with ice water and extracted with ethyl acetate. The combined organic phase was washed with water, brine, dried over anhydrous Na_2SO_4, filtered and concentrated in vacuum to give crude **6** which was purified by column chromatography on 100–200 mesh silica gel using pet ether /ethyl acetate as eluent. Pale yellow solid. 0.19 g (91%). M. pt. 142^{0}C; IR (KBr cm^{-1}) 2926, 2343, 1738, 1600, 1216; ^{1}H NMR (400 MHz, DMSO-d_6): δ_H: 9.00 (s, 1H), 8.05 - 7.99 (m, 4H), 7.56 (d, J = 8.5 Hz, 2H), 7.34 (t, J = 8.8 Hz, 2H), 2.71 (s, 3H); ^{13}C NMR (100 MHz, DMSO-d_6) δ_C: 160.2, 159.6, 143.0, 142.6, 136.8, 135.1, 130.9, 129.6, 127.7,* 124.5, 116.6, 14.6; LC-MS calcd. m/z 346 found 347 [(M+1)]$^+$. [*Two carbons merged here]. HRMS (ESI-TOF): m/z calcd for $C_{17}H_{13}ClFN_2OSH$ [M+H]$^+$: 347.0421, found: 347.0421.

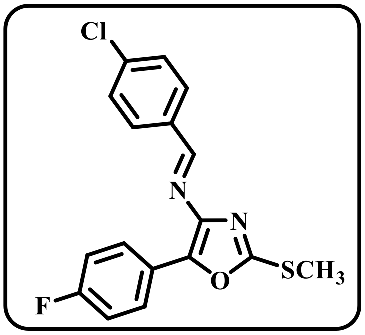

Procedure for the benzylation of 3m

(*E*)-2-(Benzylthio)-*N*-(4-chlorobenzylidene)-5-(4-fluorophenyl)oxazol-4-amine (7): To a stirred and cleaned suspension of sodium hydride (60% suspension in oil (0.028 g, 0. 70 mmol) in dry THF (2 ml) at 0 –5 °C, **3m** (0.2 g, 0.64 mmol) was added and the reaction mixture was allowed to stir at room temperature for 15 minutes, followed by addition of benzyl bromide (0.12 g, 0.70 mmol). It was further stirred for 2 hours at room temperature. After completion of the reaction (monitored by TLC), the mixture was diluted with ice water and extracted with ethyl acetate. The combined organic phase was washed with water, brine, dried over anhydrous Na_2SO_4 filtered and concentrated in vacuum to give crude **7** which was purified by column chromatography on 100–200 mesh silica gel using

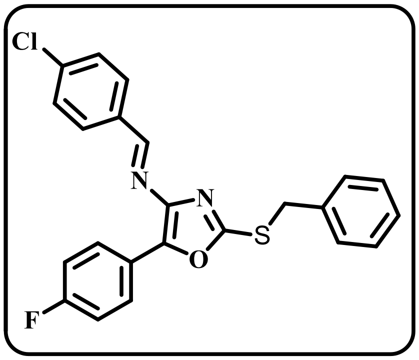

pet ether /ethyl acetate as eluent. Pale yellow solid; 0.23 g (88%). M. pt. 120^{0}C; IR (KBr cm^{-1}) 2333, 2118, 1740, 1464, 1372; ^{1}H NMR (400 MHz, DMSO-d_6): δ_H: 9.00 (s, 1H), 8.03 - 7.97 (m, 4H), 7.55 (d, J = 8.5 Hz, 2H), 7.47 (t, J = 8.4 Hz, 2H), 7.34 - 7.29 (m, 4H), 7.26 - 7.22 (m, 1H), 4.54 (s, 2H); ^{13}C NMR (100 MHz, DMSO-d_6) δ_C: 162.3, 158.5, 143.0, 142.8, 137.4, 136.8, 135.0, 131.0, 129.5,* 129.1,* 128.2, 127.7, 124.5, 116.6, 36.1; LC-MS calcd. m/z 422 found 423 [(M+1)]$^+$. HRMS (ESI-TOF): m/z calcd for $C_{23}H_{16}ClFN_2OSH$ [M+H]$^+$: 423.0734, found: 423.0732. [*Two carbons merged here].

General procedure for the preparation of 4

To a solution of azidochalcone **1** (1 mmol) in dry acetonitrile (2 mL) were added potassium thiocyanate **2** (3 mmol) and ferric nitrate (0.5 mmol). The reaction mixture was stirred magnetically at reflux for 6 h. After completion of the reaction (monitored by TLC), the product was diluted with water, extracted with ethyl acetate (15 mL) and purified by column chromatography (100–200 mesh silica gel) using ethyl acetate/petroleum ether mixture to afford product **4**.

IV Characterization data of 4

(2-Amino-5-(2,4-dichlorophenyl)thiazol-4-yl)(4-chlorophenyl)methanone (4a): Isolated yield 0.20 g (92%); off white solid; M. pt. 175-177^{0}C; IR (neat, cm^{-1}) 3586, 2330, 1662, 1527, 1329; ^{1}H NMR (400 MHz, DMSO-d_6): δ_H: 7.94 (d, J = 8.8 Hz, 2H), 7.67 (d, J = 2 Hz, 1H), 7.55 (d, J = 8.4 Hz, 2H), 7.49 - 7.42 (m, 4H); ^{13}C NMR (100 MHz, DMSO-d_6) δ_C: 187.4, 167.5, 145.9, 138.1, 136.3, 134.5, 134.2, 134.1, 132.2, 130.0, 129.5, 128.7, 127.8, 127.1; LC-MS calcd. m/z 383, found 384 [(M+1)]. HRMS (ESI-TOF): m/z calcd for $C_{16}H_{10}Cl_3N_2OSH$ [M+H]$^+$: 382.9579, found: 382.9576.

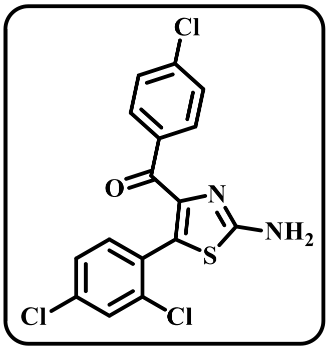

(2-Amino-5-(2,4-dichlorophenyl)thiazol-4-yl)(*p*-tolyl)methanone (4b): Isolated yield 0.19 g (88%); off white solid; M. pt. 187-189^{0}C; IR (neat, cm^{-1}) 3454, 3127, 2336, 1604, 1527, 1333, 1177, 965; ^{1}H NMR (400 MHz, DMSO-d_6): δ_H: 7.82 (d, J = 6.8 Hz, 2H), 7.65 (s, 1H), 7.43 - 7.27 (m, 6H), 2.36 (s, 3H); ^{13}C NMR (100 MHz, DMSO-d_6) δ_C: 188.5, 167.5, 146.7, 143.6, 135.0, 134.5, 134.0, 134.0, 130.5, 130.2, 129.5, 129.1, 127.8, 125.8,

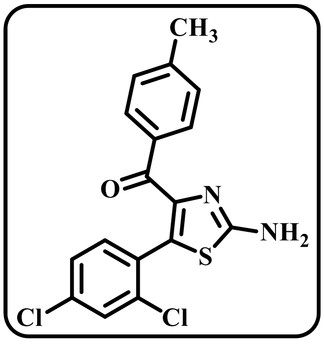

21.7; LC-MS calcd. m/z 363, found 364 [(M+1)]$^+$. HRMS (ESI-TOF): m/z calcd for $C_{17}H_{12}Cl_2N_2OSH$ [M+H]$^+$: 363.0126, found: 363.0122.

(2-Amino-5-(3-bromophenyl)thiazol-4-yl)(phenyl)methanone (4c)[1]: Isolated yield 0.20 g (90%); pale yellow solid; M. pt. 154-155^{0}C; IR (neat, cm^{-1}) 3583, 2364, 1654, 1526, 1329, 1076; ^{1}H NMR (400 MHz, DMSO-*d*$_6$): δ_H: 7.85 (t, *J* = 7.6 Hz, 2H), 7.62 (t, *J* = 7.6 Hz, 1H), 7.50 - 7.45 (m, 6H), 7.32 - 7.25 (m, 2H); ^{13}C NMR (100 MHz, DMSO-*d*$_6$) δ_C: 190.9, 167.0, 144.9, 137.5, 133.9, 133.7, 131.2, 131.1, 130.8, 130.2, 128.8, 128.1, 127.8, 122.1; LC-MS calcd. m/z 359, found 360 [(M+1)]$^+$. HRMS (ESI-TOF): m/z calcd for $C_{16}H_{11}BrN_2OSH$ [M+H]$^+$: 358.9854, found: 358.9853.

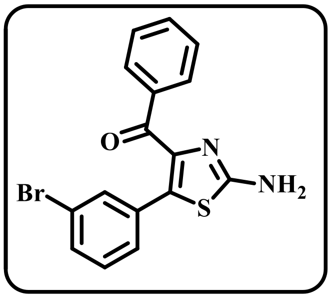

(2-Amino-5-(4-chlorophenyl)thiazol-4-yl)(4-chlorophenyl)methanone (4d): Isolated yield 0.196 g (93%); pale yellow solid M. pt. 237-239^{0}C; IR (neat, cm^{-1}) 3583, 3426, 2364, 1640, 1533, 1090, 856; ^{1}H NMR (400 MHz, DMSO-*d*$_6$): δ_H: 7.88 (d, *J* = 8.4 Hz, 2H), 7.56 (d, *J* = 8.4 Hz, 2H), 7.46 (s, 2H), 7.38 (d, *J* = 8.4 Hz, 2H), 7.33 (d, *J* = 8.4 Hz, 2H); ^{13}C NMR (100 MHz, DMSO-*d*$_6$) δ_C: 189.3, 166.7, 144.0, 138.5, 136.3, 132.8, 132.1, 130.8, 130.4, 129.4, 129.0, 128.9; LC-MS calcd. m/z 349, found 350 [(M+1)]$^+$. HRMS (ESI-TOF): m/z calcd for $C_{16}H_{10}Cl_2N_2OSH$ [M+H]$^+$: 348.9969, found: 348.9969.

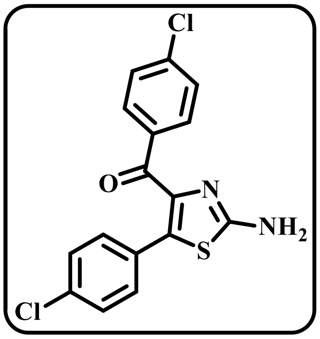

(2-Amino-5-(3-bromophenyl)thiazol-4-yl)(4-fluorophenyl)methanone (4e): Isolated yield 0.18 g (83%); pale yellow solid; M. pt. 179-181^{0}C; IR (neat, cm^{-1}) 3646, 2712, 1511, 1260, 1173; ^{1}H NMR (400 MHz, DMSO-*d*$_6$): δ_H: 7.95 (bs, 2H), 7.48 - 7.44 (m, 4H), 7.32 - 7.26 (m, 4H); ^{13}C NMR (100 MHz, DMSO-*d*$_6$) δ_C: 189.1, 167.0, 165.3, 144.5, 134.2, 133.9, 133.3, 133.2, 131.2, 130.9, 128.3, 128.2, 122.1, 115.9; LC-MS calcd.m/z 377, found 378 [(M+1)]$^+$. HRMS (ESI-TOF): m/z calcd for $C_{16}H_{10}BrFN_2OSH$ [M+H]$^+$: 376.9759, found: 376.9760.

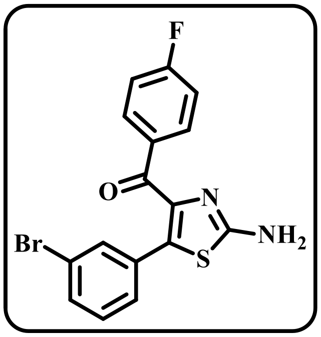

(2-Amino-5-(4-chlorophenyl)thiazol-4-yl)(phenyl)methanone (4f): Isolated yield 0.19 g (89%); pale yellow solid; M. pt. 208-210^{0}C; IR (neat, cm^{-1}) 3583, 2366, 1651, 1527, 1332, 1092, 859; ^{1}H NMR (400 MHz, DMSO-d_6): δ_H: 7.84 (t, J = 8 Hz, 2H), 7.61 (t, J = 7.6 Hz, 1H), 7.49 - 7.42 (m, 4H), 7.37 - 7.30 (m, 4H); ^{13}C NMR (100 MHz, DMSO-d_6) δ_C: 191.0, 166.8, 144.5, 137.5, 133.7, 132.7, 130.6, 130.5, 130.2, 129.0, 128.8, 128.3; LC-MS calcd. m/z 314, found 315[(M+1)]$^+$. HRMS (ESI-TOF): m/z calcd for $C_{16}H_{12}ClN_2OSH$ [M+H]$^+$: 315.0359, found: 315.0357.

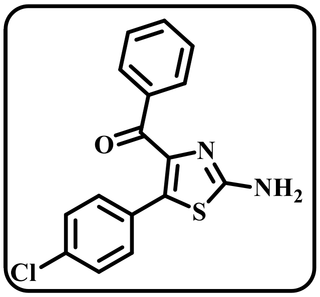

(2-Amino-5-(2,4-dichlorophenyl)thiazol-4-yl)(phenyl)methanone (4g): Isolated yield 0.19 g (87%); off white solid; M. pt. 164-166^{0}C; IR (neat, cm^{-1}) 3427, 2108, 1653, 1607, 1527, 1336; ^{1}H NMR (400 MHz, DMSO-d_6): δ_H: 7.87 (t, J = 7.6 Hz, 2H), 7.64 (d, J = 1.6 Hz, 1H), 7.56 (t, J = 7.2 Hz, 1H), 7.47 - 7.39 (m, 6H); ^{13}C NMR (100 MHz, DMSO-d_6) δ_C: 188.9, 167.5, 146.4, 141.0, 137.7, 134.5, 134.1, 133.1, 130.2, 130.1, 129.5, 128.5, 127.8, 126.5; LC-MS calcd. m/z 349, found 350 [(M+1)]$^+$. HRMS (ESI-TOF): m/z calcd for $C_{16}H_{10}Cl_2N_2OSH$ [M+H]$^+$: 348.9969, found: 348.9967.

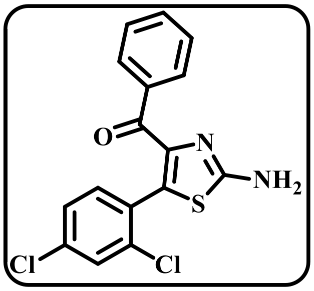

(2-Amino-5-(2,4-dichlorophenyl)thiazol-4-yl)(4-fluorophenyl)methanone (4h): Isolated yield 0.19 g (88%); pale yellow solid; M. pt. 207-209^{0}C; IR (neat, cm^{-1}) 3097, 2926, 2345, 1506, 1188, 1062; ^{1}H NMR (400 MHz, DMSO-d_6): δ_H: 8.02 - 7.98 (m, 2H), 7.65 (s, 1H), 7.48 - 7.42 (m, 4H), 7.28 (t, J = 8.8 Hz, 2H); ^{13}C NMR (100 MHz, DMSO-d_6) δ_C: 187.1, 167.5, 165.1, 146.1, 134.5, 134.1, 134.0, 133.3, 133.2, 130.1, 129.5, 127.8, 126.8, 115.6; LC-MS calcd. m/z 367, found 368 [(M+1)]$^+$. HRMS (ESI-TOF): m/z calcd for $C_{16}H_9Cl_2FN_2OSH$ [M+H]$^+$: 366.9875, found: 366.9873.

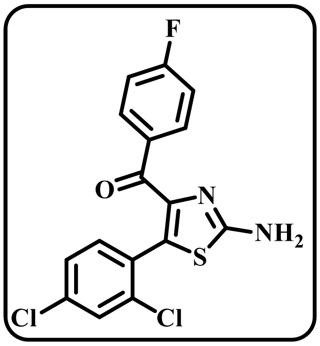

(2-Amino-5-(4-bromophenyl)thiazol-4-yl)(3,4-dichlorophenyl)methanone (4i): Isolated yield 0.20 g (93%); pale yellow solid; M. pt. 213-215^{0}C; IR (neat, cm^{-1}) 3583, 2366, 1602, 1530, 1140; ^{1}H NMR (400 MHz, DMSO-d_6): δ_H: 8.25 (s, 1H), 8.15 (d, J = 8.4 Hz, 1H), 7.85 (d, J = 8.4 Hz, 1H), 7.51 - 7.48 (m, 4H), 7.31 - 7.30 (m, 2H); ^{13}C NMR (100 MHz, DMSO-d_6)

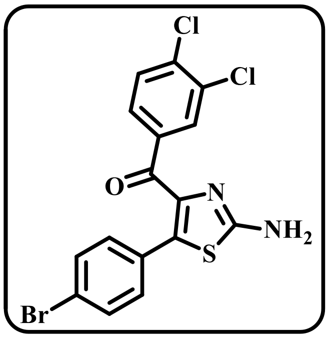

δ_C: 186.9, 166.6, 143.0, 137.0, 135.6, 132.2, 131.8, 130.7, 129.4, 127.0, 126.7, 126.4, 124.3, 121.6; LC-MS calcd. m/z 428, found 429 $[(M+1)]^+$. HRMS (ESI-TOF): m/z calcd for $C_{16}H_9BrCl_2N_2OSK$ $[M+K]^+$: 464.8633, found: 464.9291.

(2-Amino-5-(2,4-dichlorophenyl)thiazol-4-yl)(3-chloro-4-fluorophenyl)methanone (4j): Isolated yield 0.18 g (82%); off white solid; M. pt. 184-186^{0}C; IR (neat, cm^{-1}) 3625, 3084, 2366, 1690, 1529, 1328; ^1H NMR (400 MHz, DMSO-d_6): δ_H: 7.80 (s, 1H), 7.79 (d, J = 9.0 Hz, 1H), 7.38 - 7.22 (m, 4H), 7.05 - 7.00 (m, 2H); ^{13}C NMR (100 MHz, DMSO-d_6) δ_C: 186.6, 166.3, 159.8, 143.2, 135.0, 133.3, 132.3, 131.2, 131.1, 130.6, 130.5, 129.8, 128.0, 121.4, 119.6, 116.7; LC-MS calcd. m/z 401, found 402 $[(M+1)]^+$. HRMS (ESI-TOF): m/z calcd for $C_{16}H_8Cl_3FN_2OSH$ $[M+H]^+$: 400.9485, found: 400.9482.

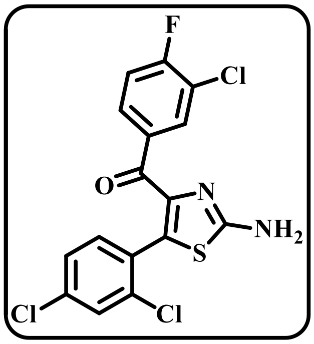

(2-Amino-5-(3-bromophenyl)thiazol-4-yl)(3-chloro-4-fluorophenyl)methanone (4k): Isolated yield 0.17 g (81%); pale yellow solid; M. pt. 157-159^{0}C; IR (neat, cm^{-1}) 3675, 2854, 1260, 1173, 1035; ^1H NMR (400 MHz, DMSO-d_6): δ_H: 8.07 (d, J = 7.6 Hz, 1H), 7.90 (s, 1H), 7.55 - 7.52 (m, 5H), 7.33 - 7.28 (m, 2H); ^{13}C NMR (100 MHz, DMSO-d_6) δ_C: 187.1, 166.8, 160.5, 143.7, 135.5, 135.4, 133.8, 132.9, 131.7, 131.1, 130.9, 130.4, 128.6, 122.0, 120.1, 117.4; LC-MS calcd. m/z 411, found 412 $[(M+1)]^+$. HRMS (ESI-TOF): m/z calcd for $C_{16}H_9BrClFN_2OSH$ $[M+H]^+$: 410.9370, found: 410.9373.

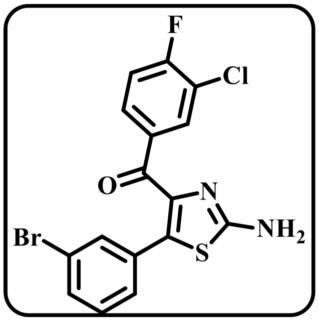

(2-Amino-5-(4-chlorophenyl)thiazol-4-yl)(4-fluorophenyl)methanone (4l): Isolated yield 0.20 g (91%); pale yellow solid; M. pt. 228-230^{0}C; IR (neat, cm^{-1}) 3428, 3114, 2362, 1643, 1537, 1337; ^1H NMR (400 MHz, DMSO-d_6): δ_H: 7.97 - 7.94 (m, 2H), 7.45 - 7.30 (m, 8H), ^{13}C NMR (100 MHz, DMSO-d_6) δ_C: 189.1, 166.8, 164.2, 144.2, 134.2, 133.2, 132.8, 130.7, 130.5, 129.0, 128.8, 115.9; LC-MS calcd. m/z 332, found 333 $[(M+1)]^+$. HRMS (ESI-TOF): m/z calcd for $C_{16}H_{10}ClFN_2OSH$ $[M+H]^+$: 333.0265, found: 333.0262.

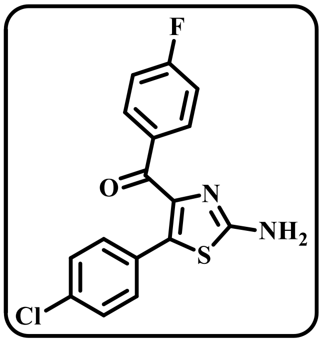

V Copies of NMR spectra of 3

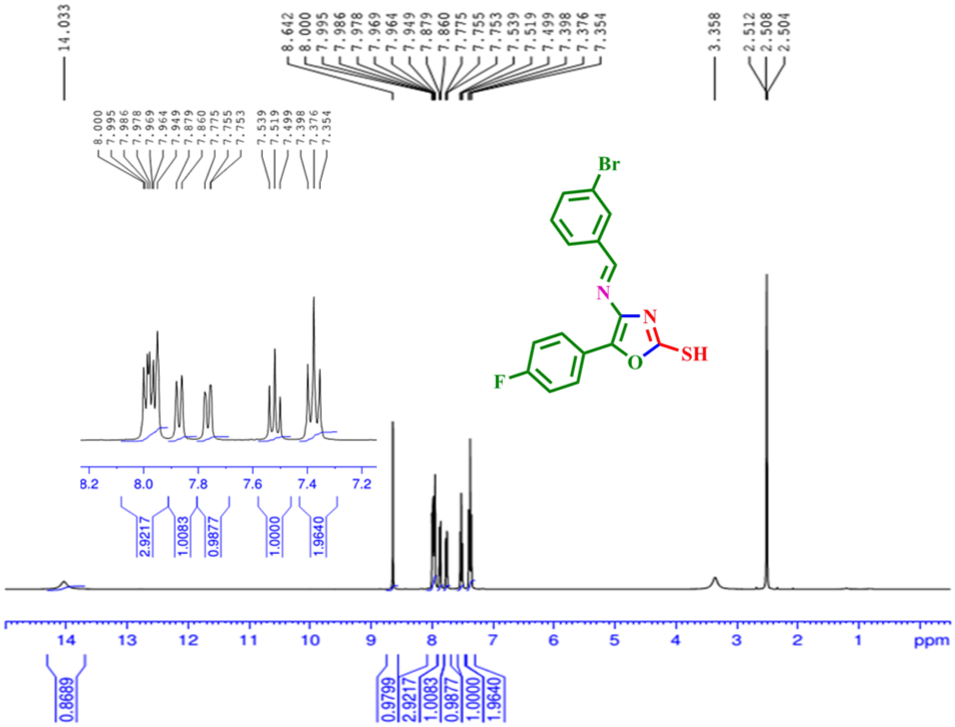

Figure S1. ^{1}H NMR **spectrum of 3a**.

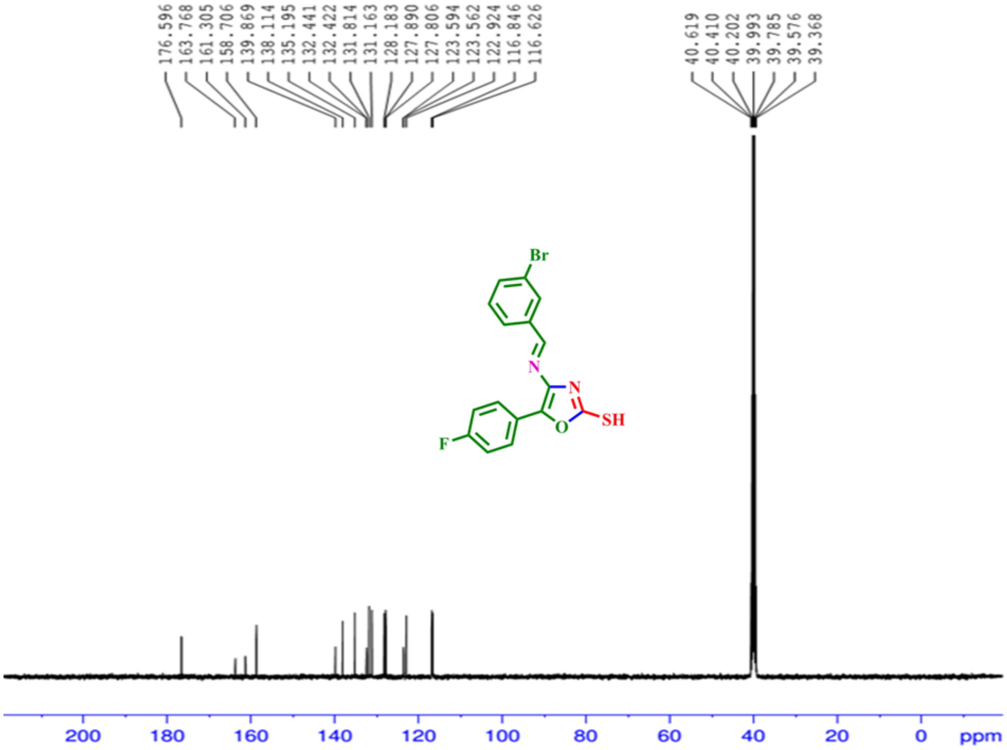

Figure S2. ^{13}C NMR spectrum of **3a**.

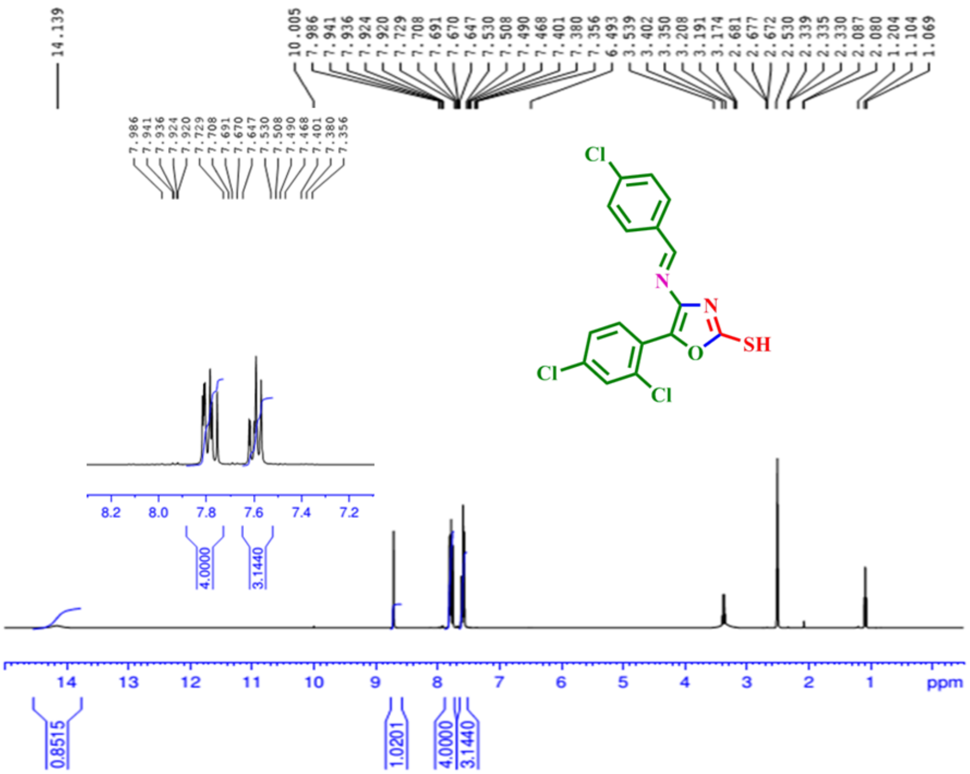

Figure S3. ^{1}H NMR spectrum of **3b**.

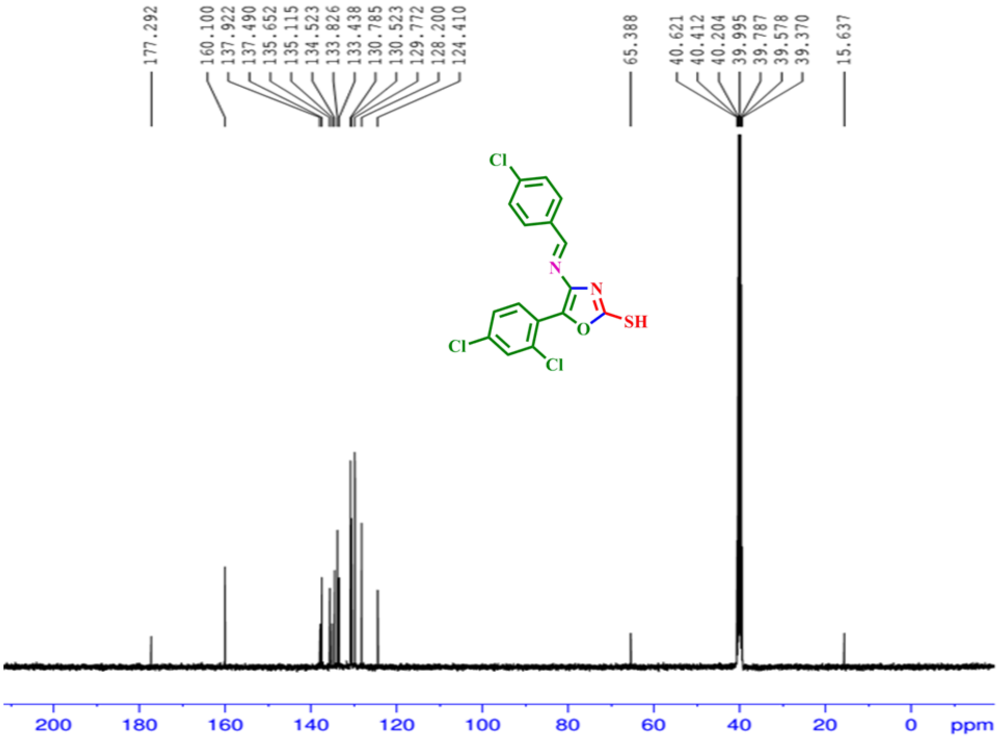

Figure S4. ^{13}C NMR spectrum of **3b**.

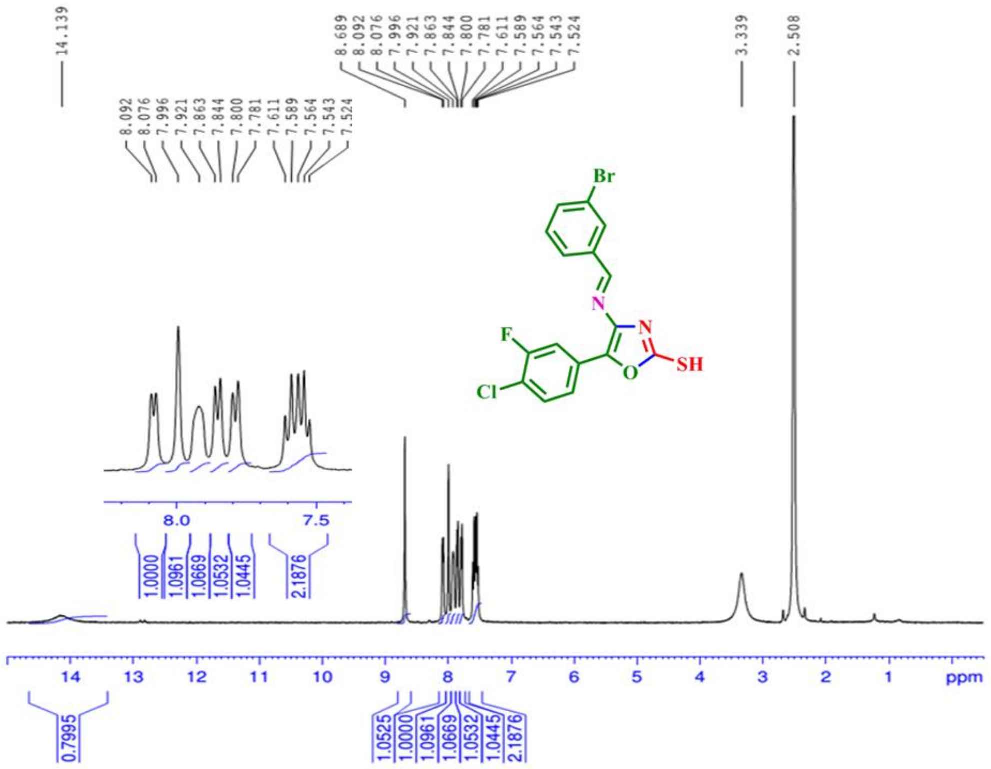

Figure S5. ^{1}H NMR spectrum of **3c**.

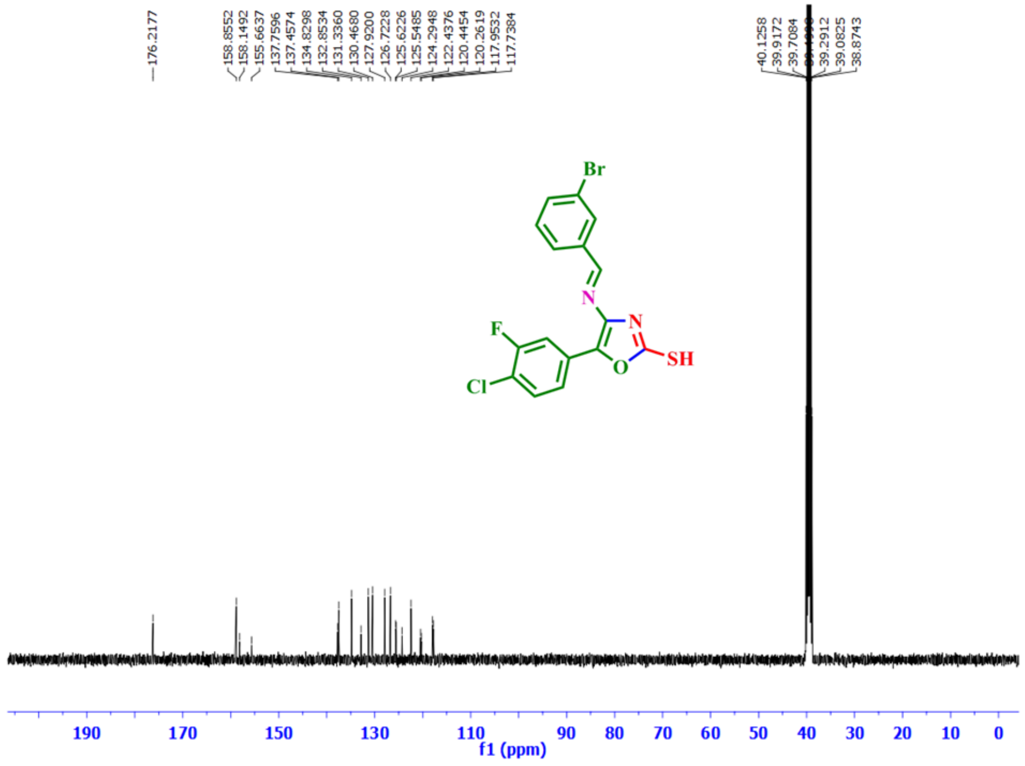

Figure S6. ^{13}C NMR spectrum of **3c**.

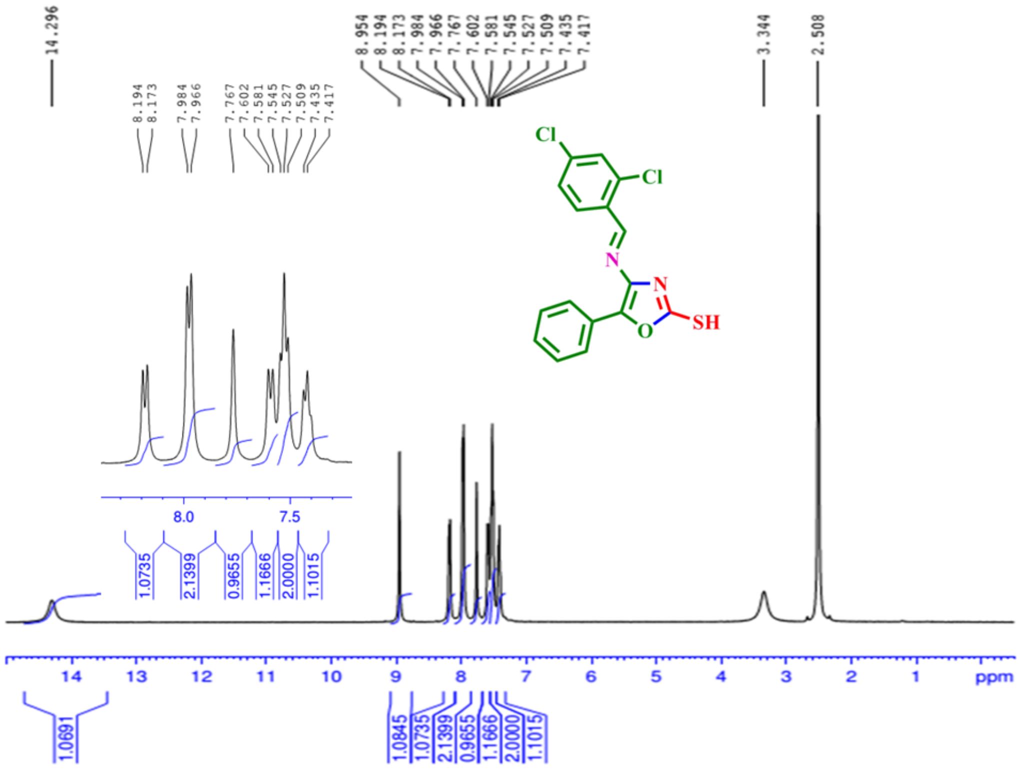

Figure S7. ^{1}H NMR spectrum of **3d**.

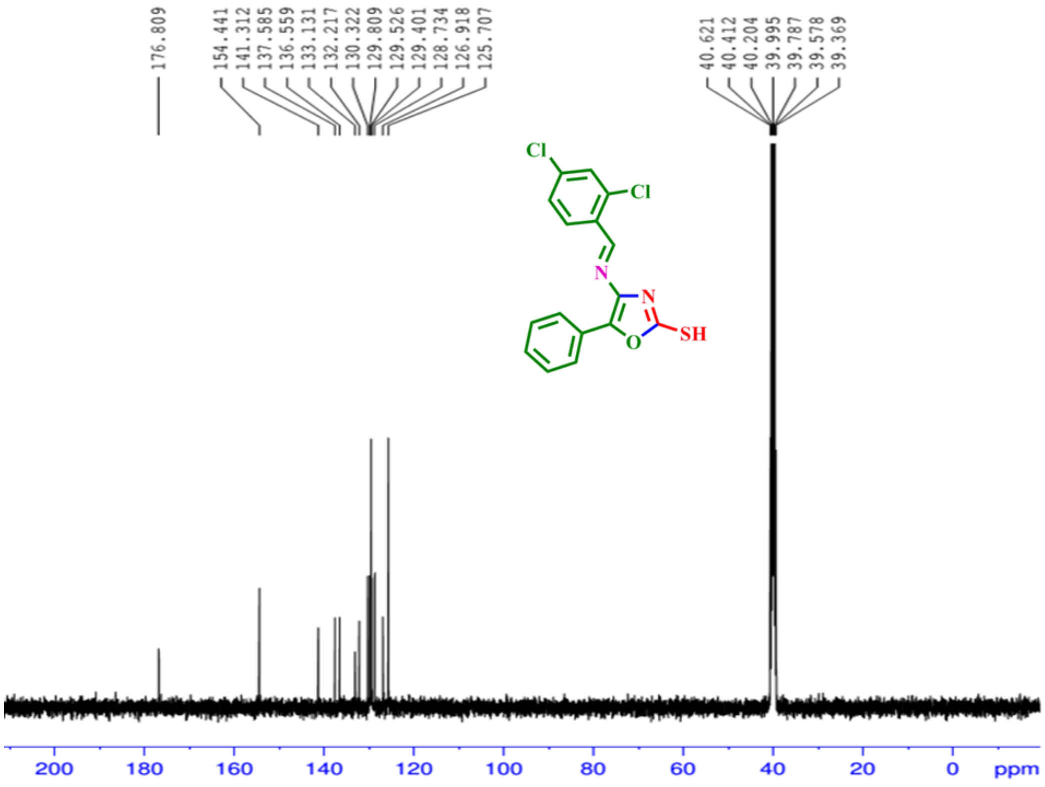

Figure S8. ^{13}C NMR spectrum of **3d**.

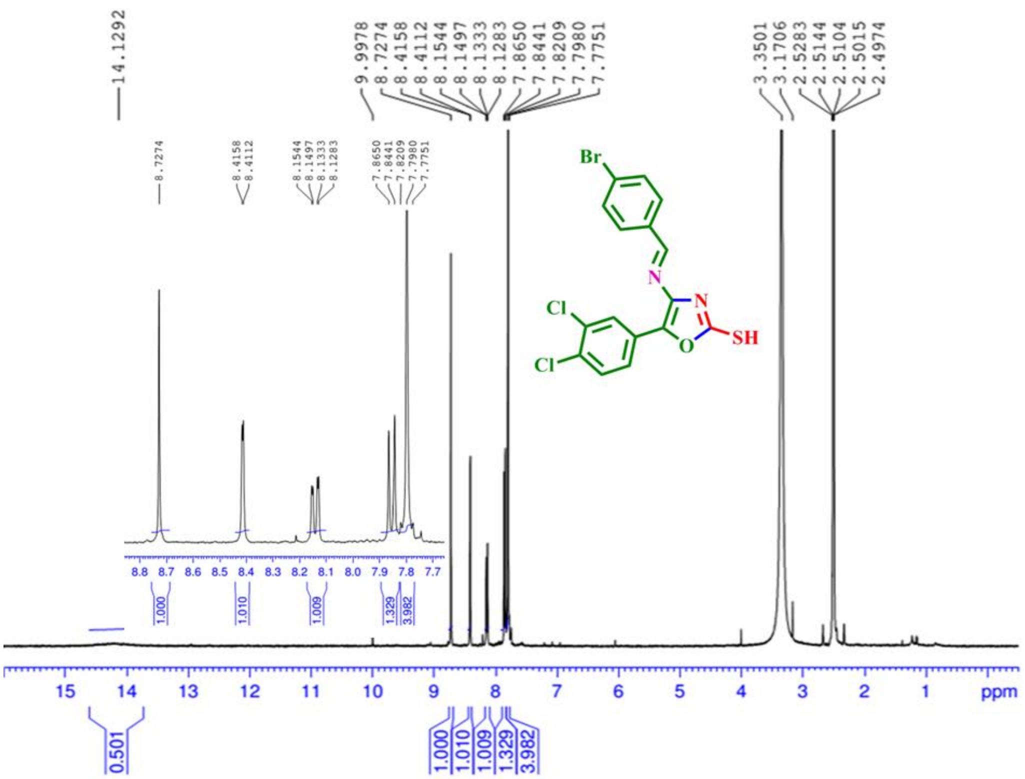

Figure S9. ^{1}H NMR spectrum of **3e**.

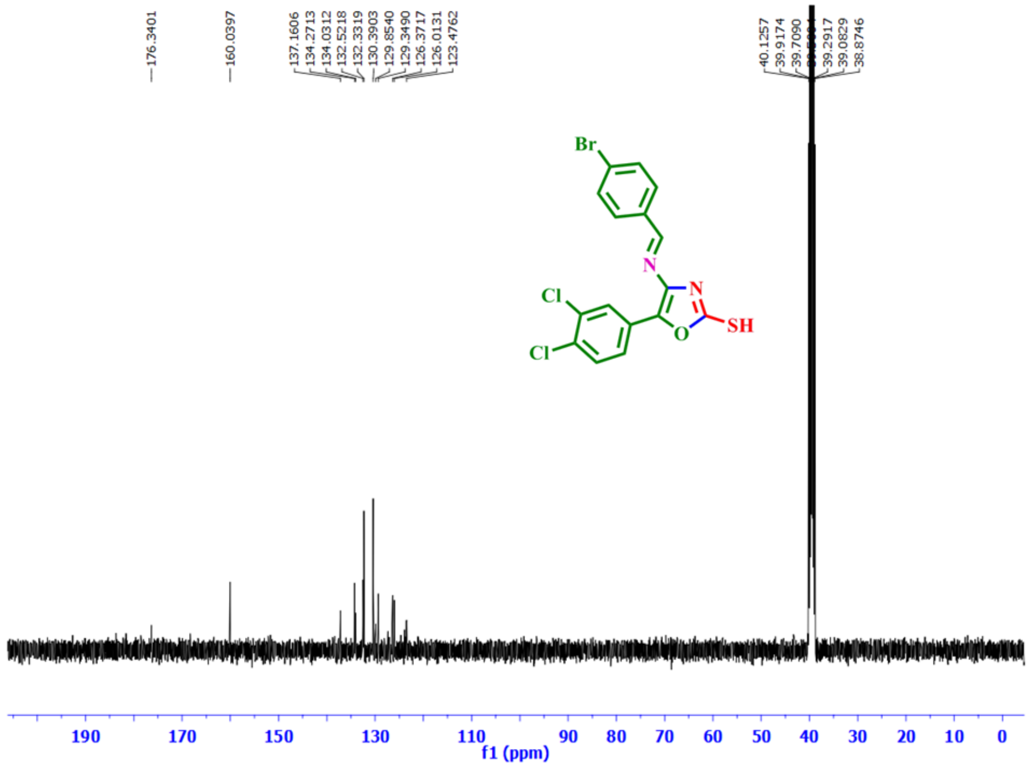

Figure S10. ^{13}C NMR spectrum of **3e**.

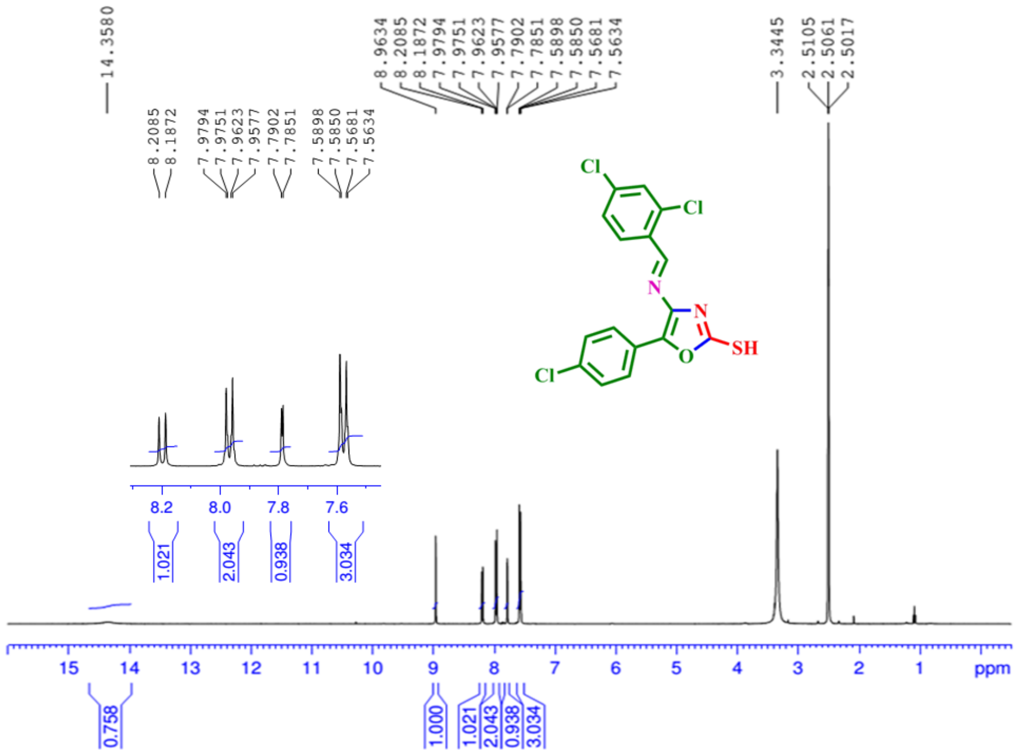

Figure S11. ^{1}H NMR spectrum of **3f**.

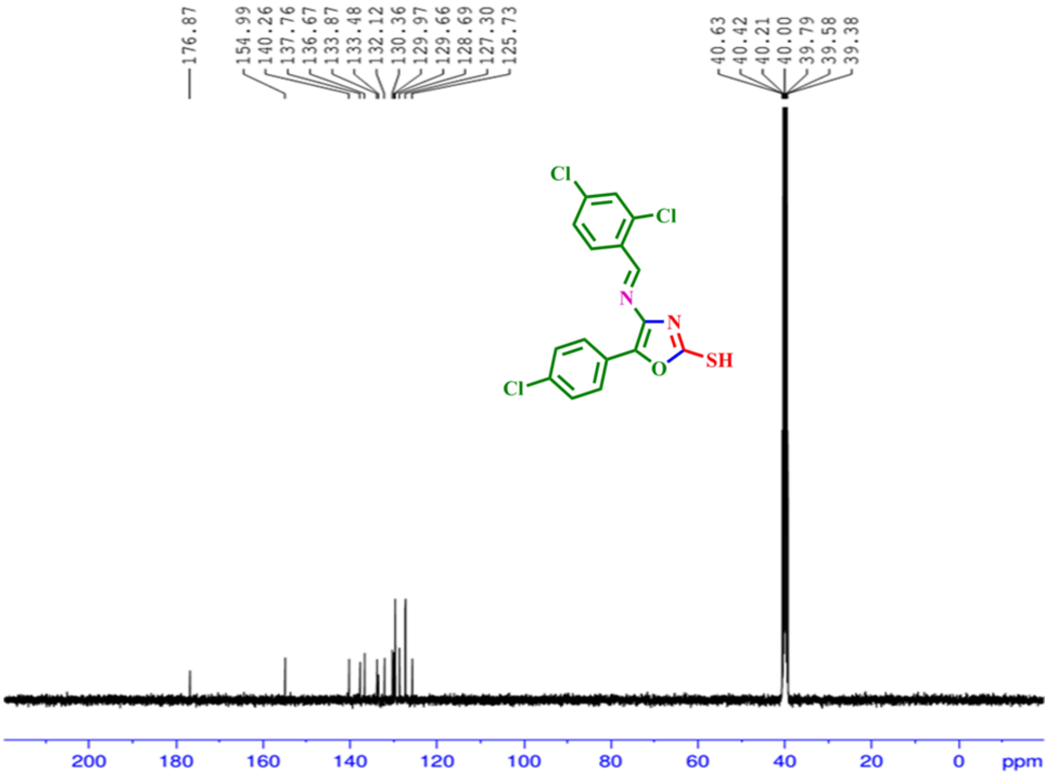

Figure S12. ^{13}C NMR spectrum of **3f**.

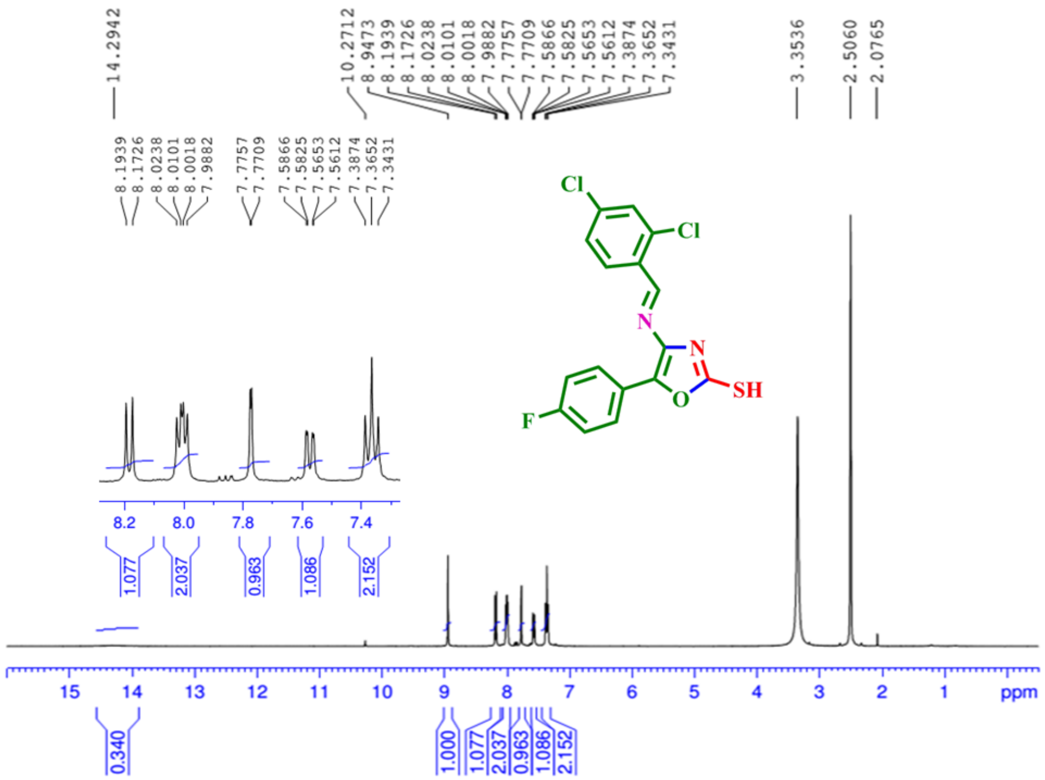

Figure S13. ^{1}H NMR spectrum of **3g**.

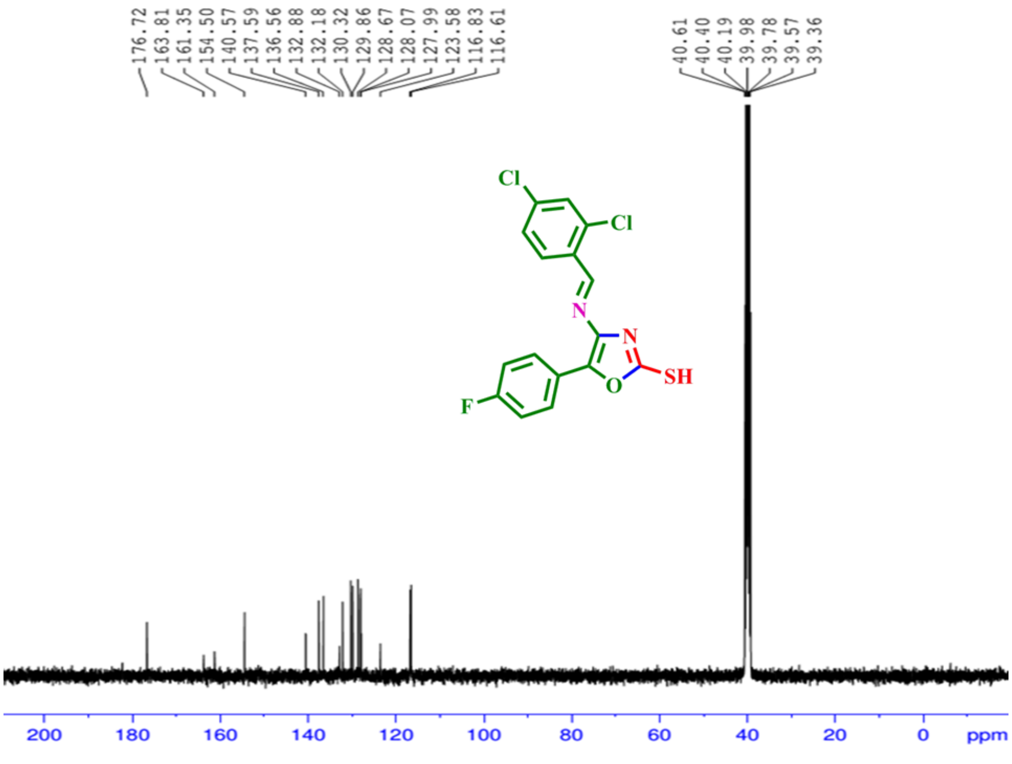

Figure S14. ^{13}C NMR spectrum of **3g**.

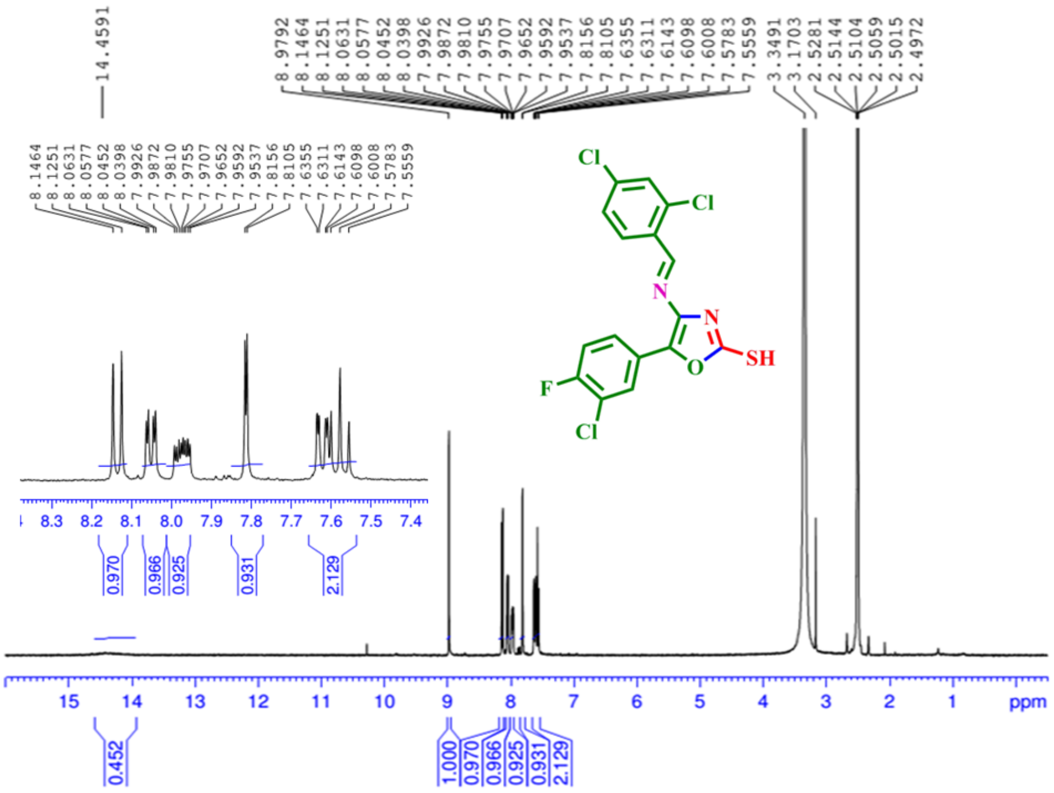

Figure S15. ^{1}H NMR spectrum of **3h**.

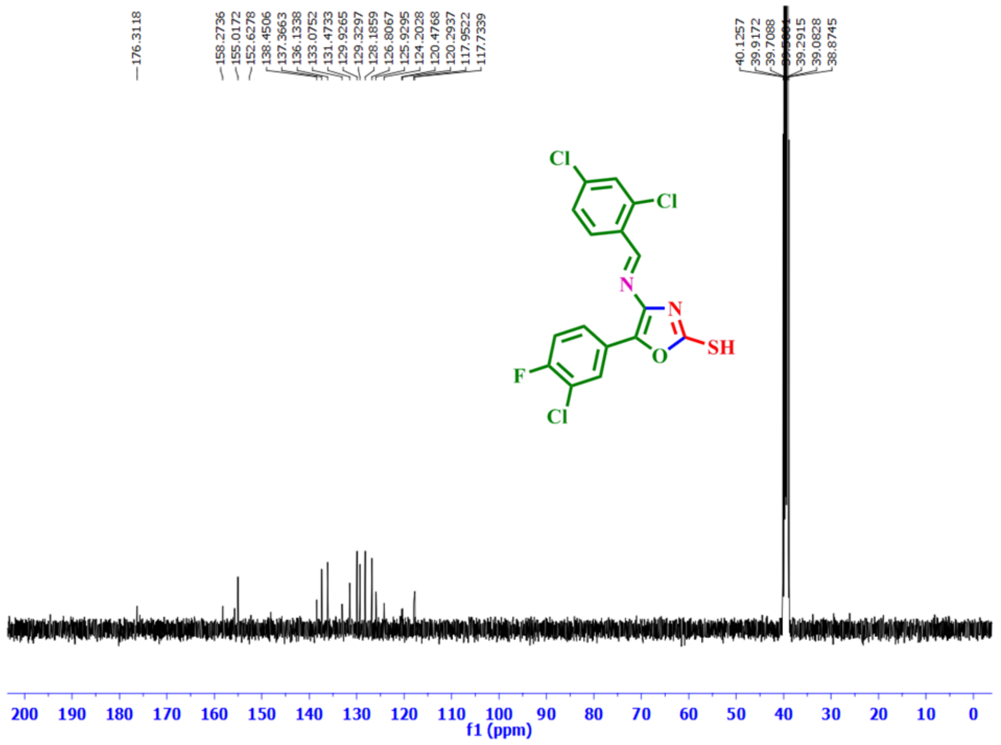

Figure S16. ^{13}C NMR spectrum of **3h**.

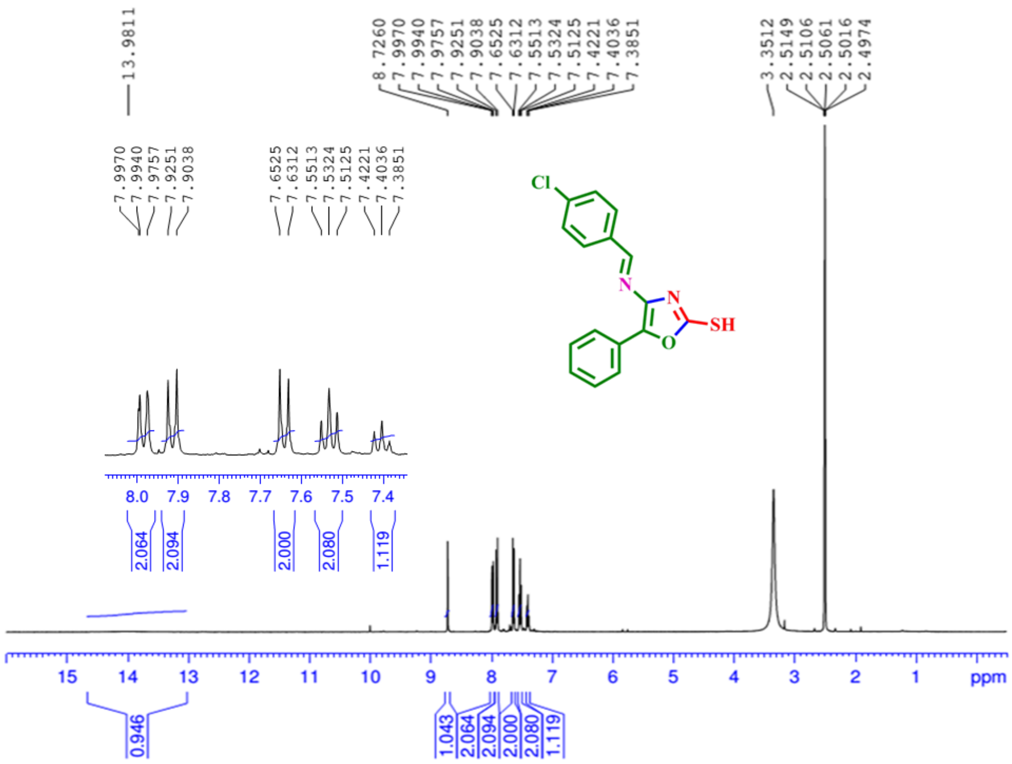

Figure S17. ^{1}H NMR spectrum of **3i**.

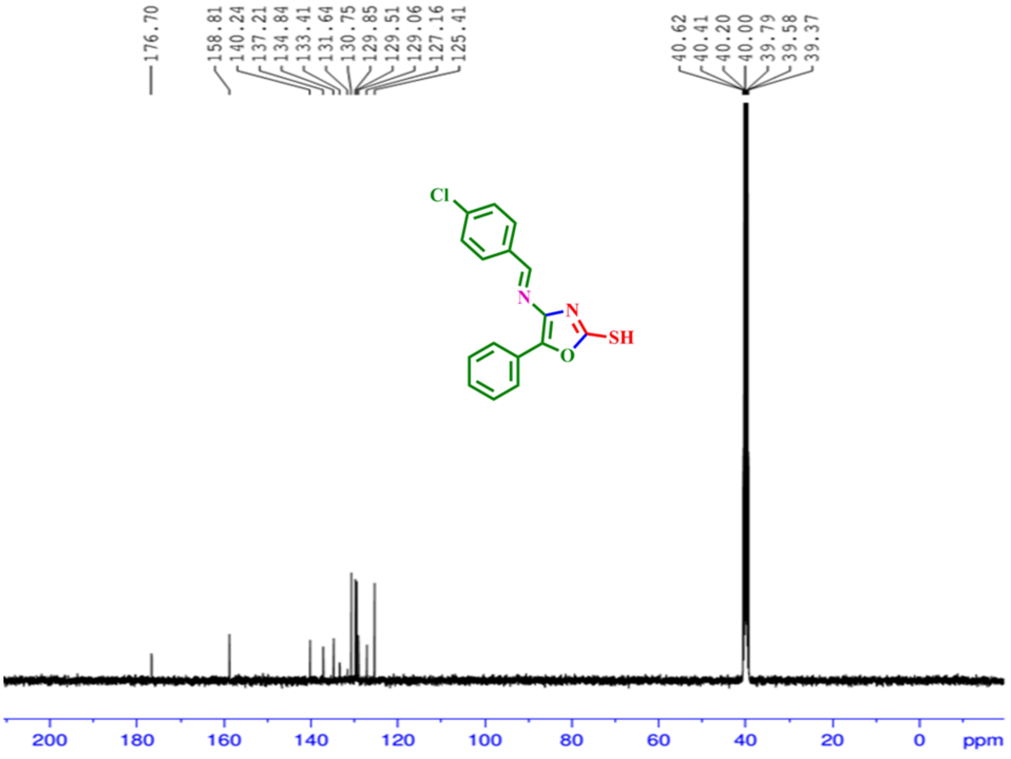

Figure S18. ^{13}C NMR spectrum of **3i**.

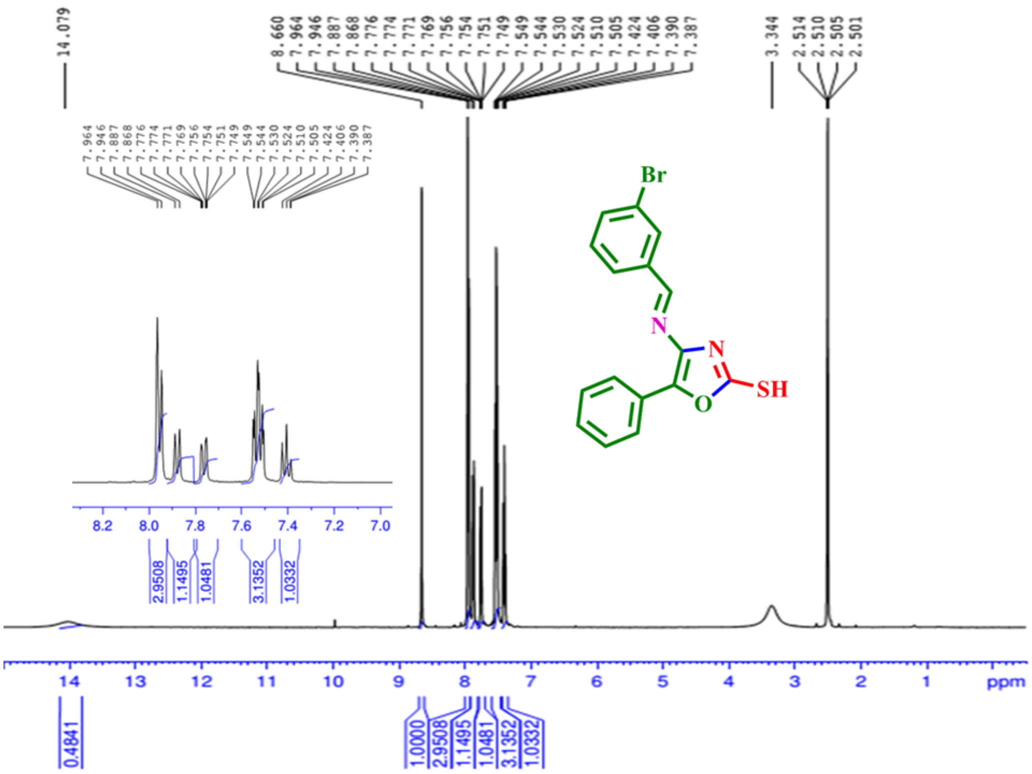

Figure S19. ^{1}H NMR spectrum of **3j**.

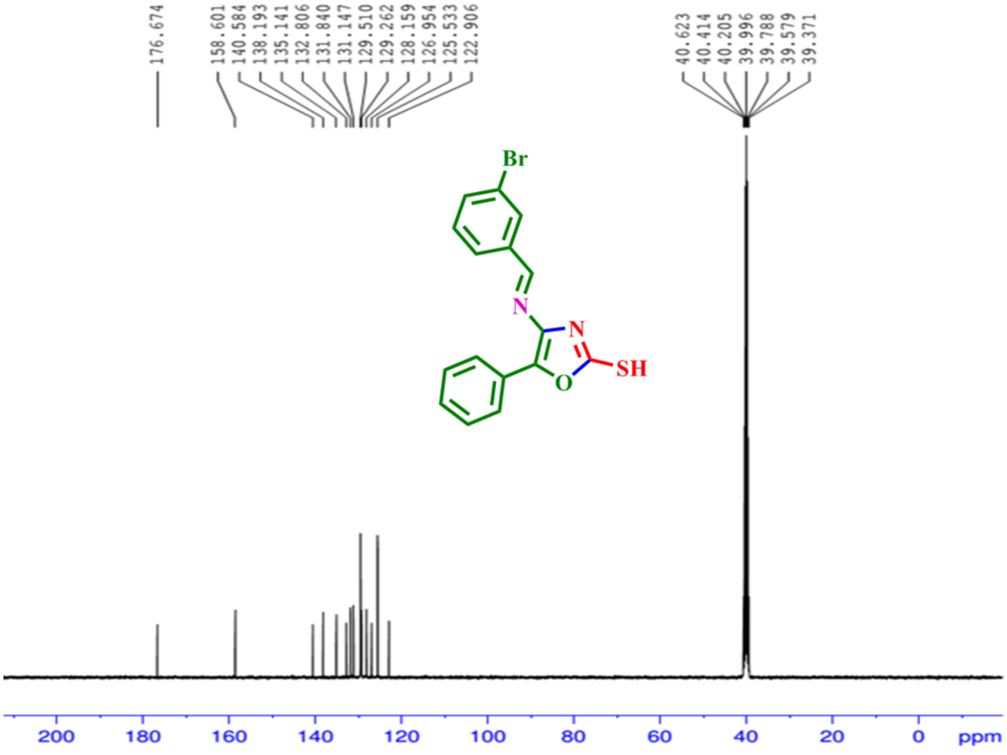

Figure S20. ^{13}C NMR spectrum of **3j**.

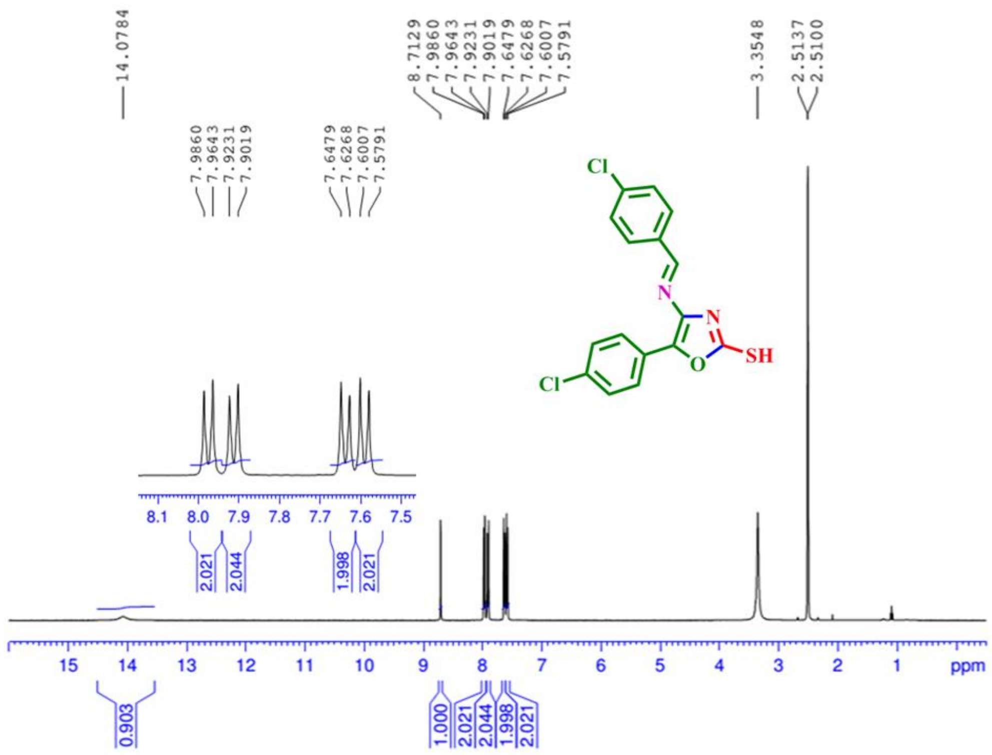

Figure S21. ^{1}H NMR spectrum of **3k**.

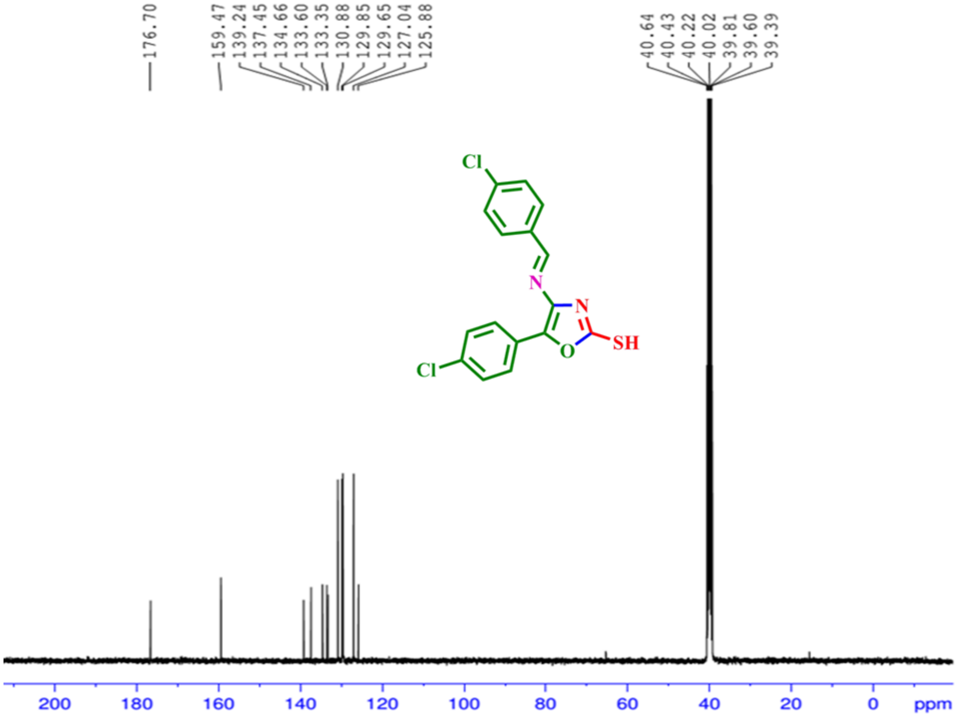

Figure S22. ^{13}C NMR spectrum of **3k**.

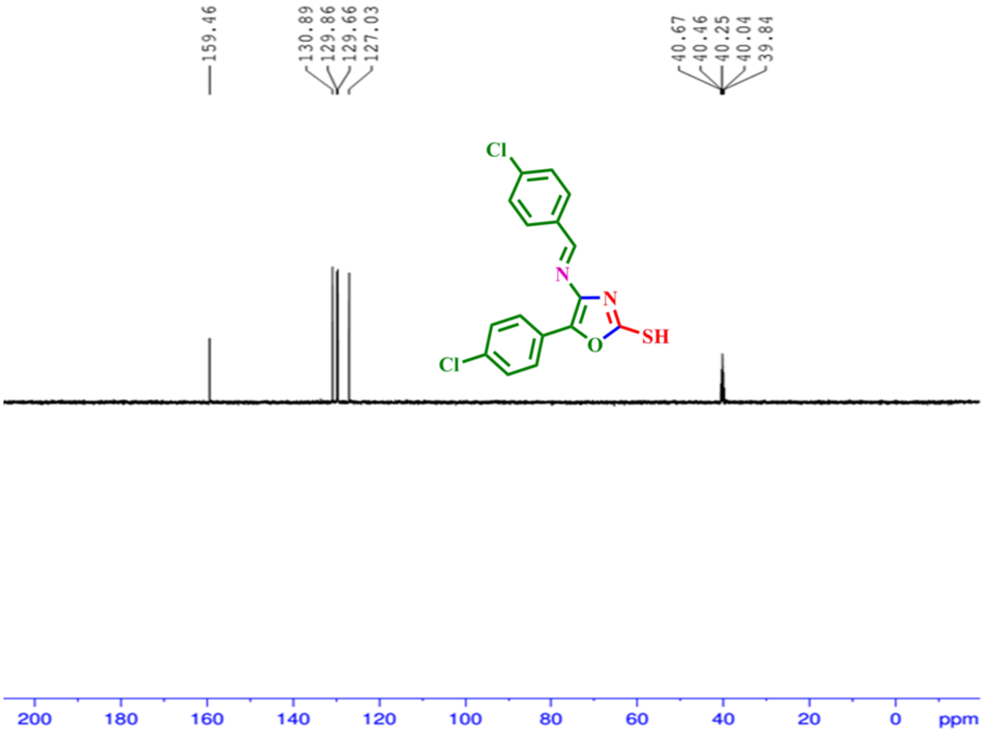

Figure S23. DEPT (135) spectrum of **3k**.

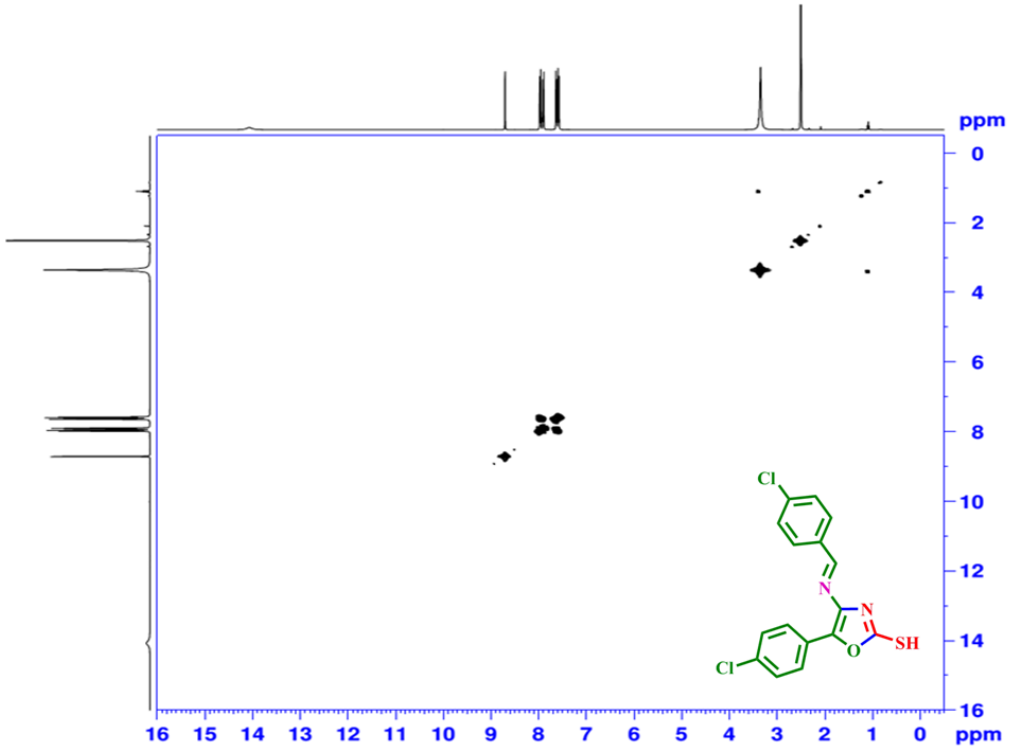

Figure S24. H-H COSY spectrum of **3k**.

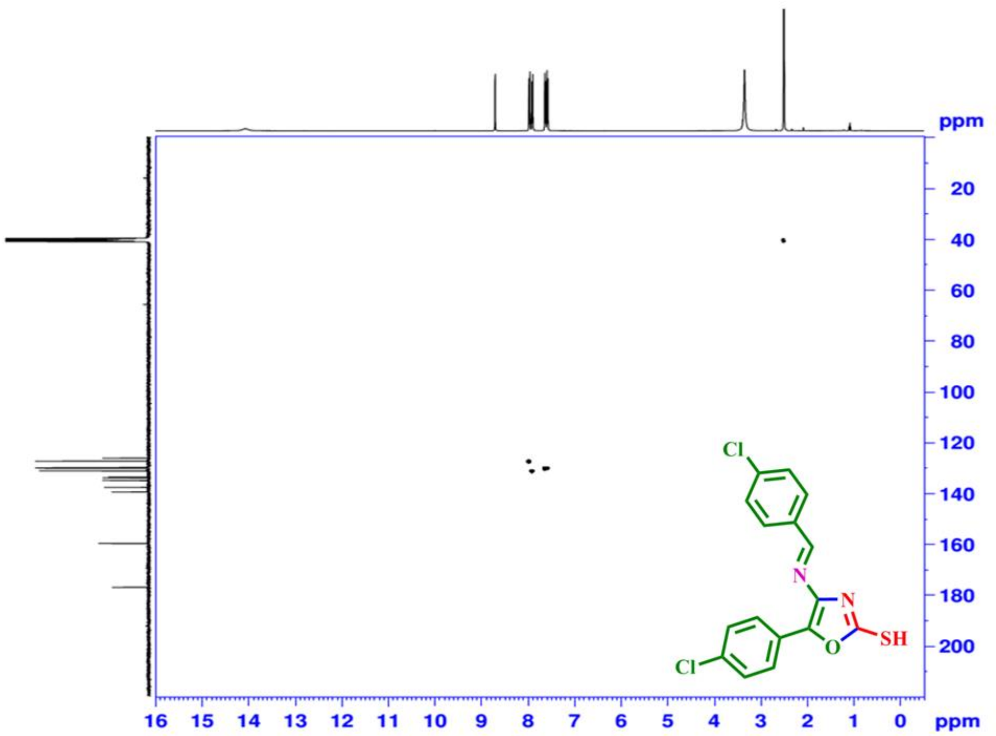

Figure S25. C-H COSY spectrum of **3k**.

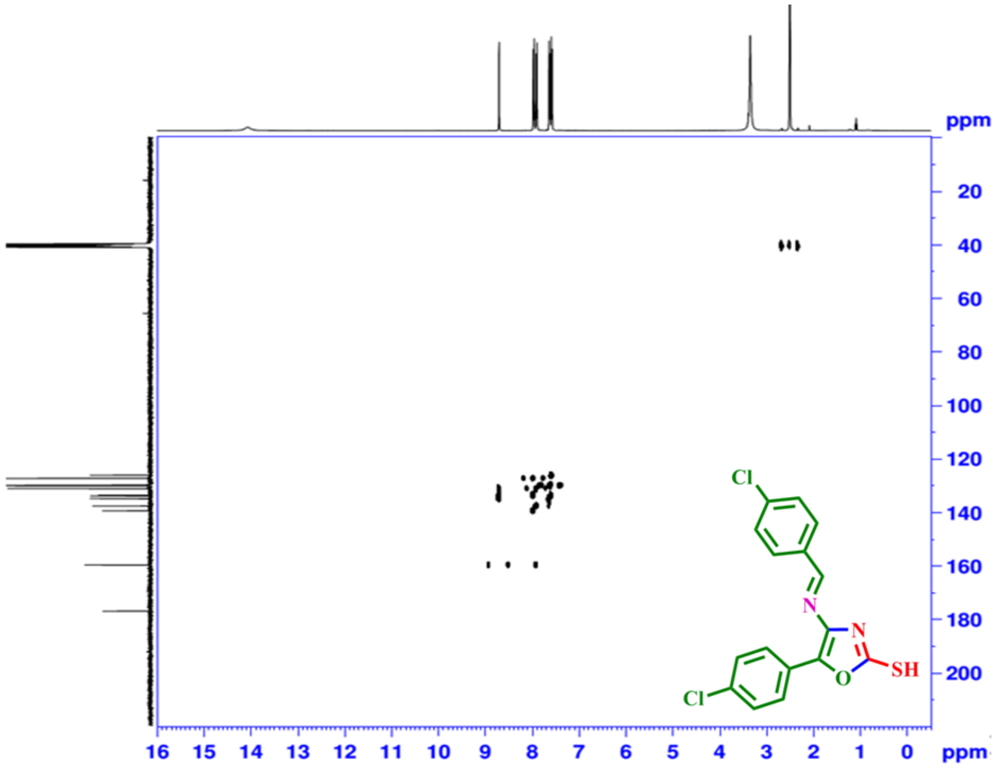

Figure S26. HMBC spectrum of **3k**.

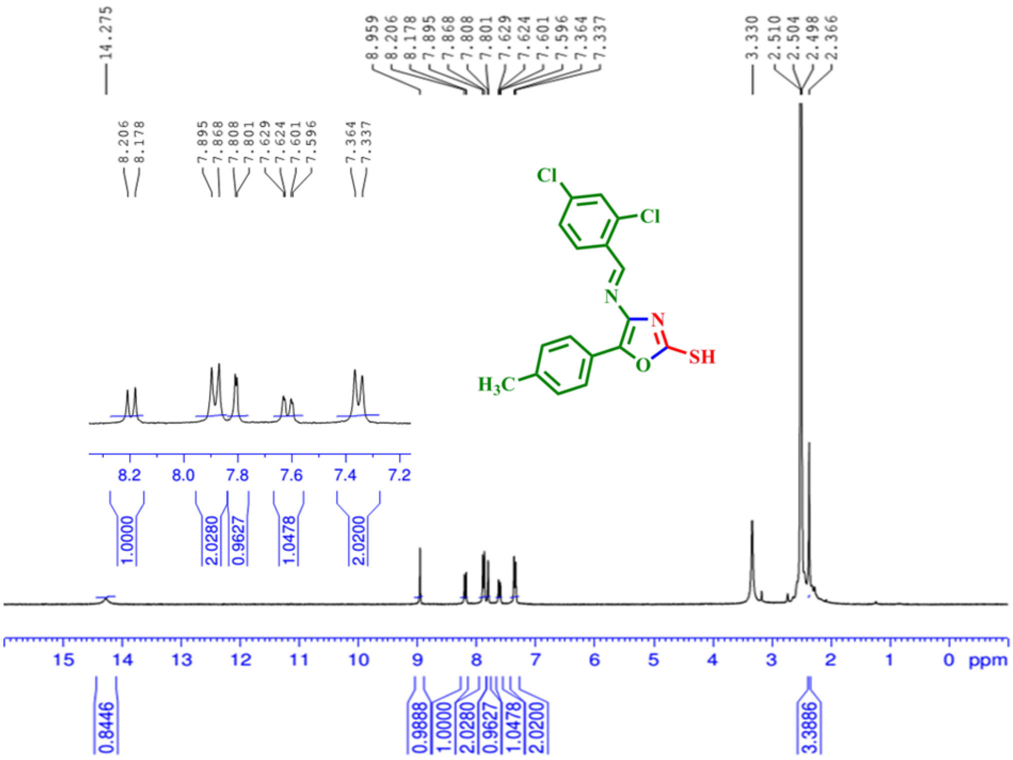

Figure S27. ^{1}H NMR spectrum of **3l**.

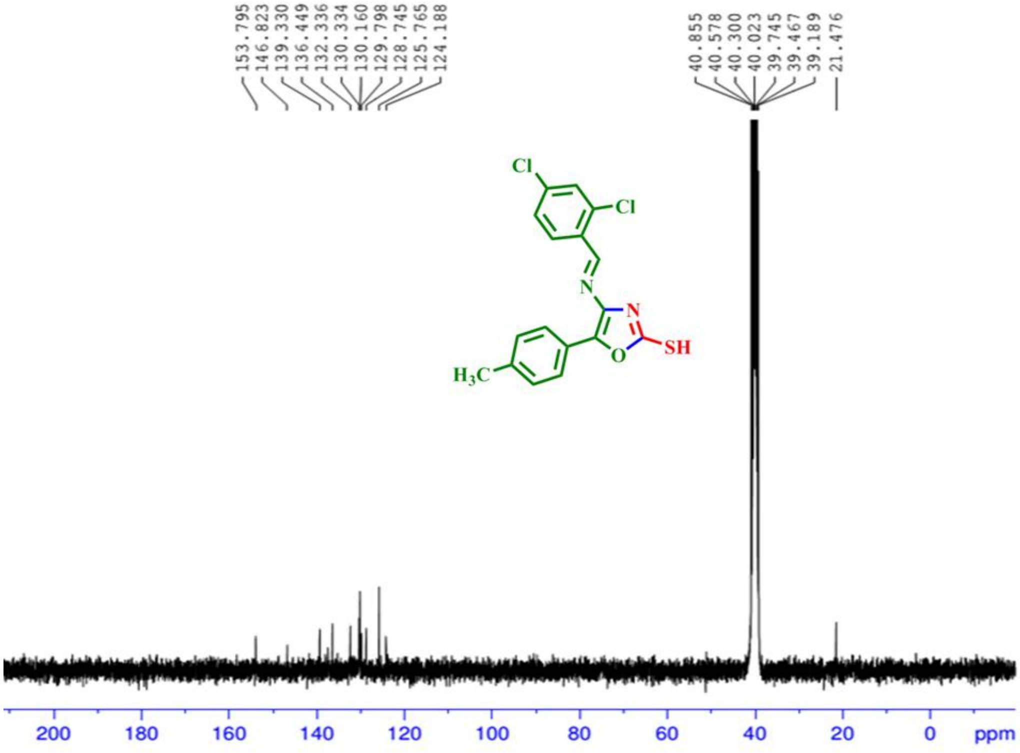

Figure S28. ^{13}C NMR spectrum of **3l**.

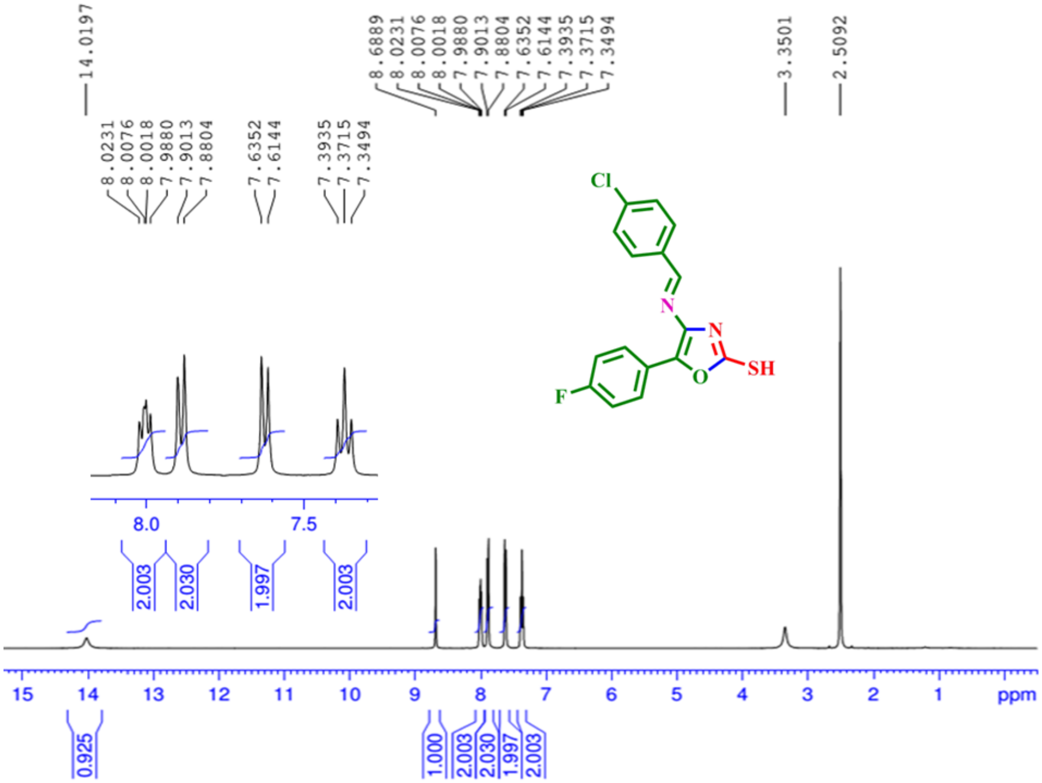

Figure S29. ^{1}H NMR spectrum of **3m**.

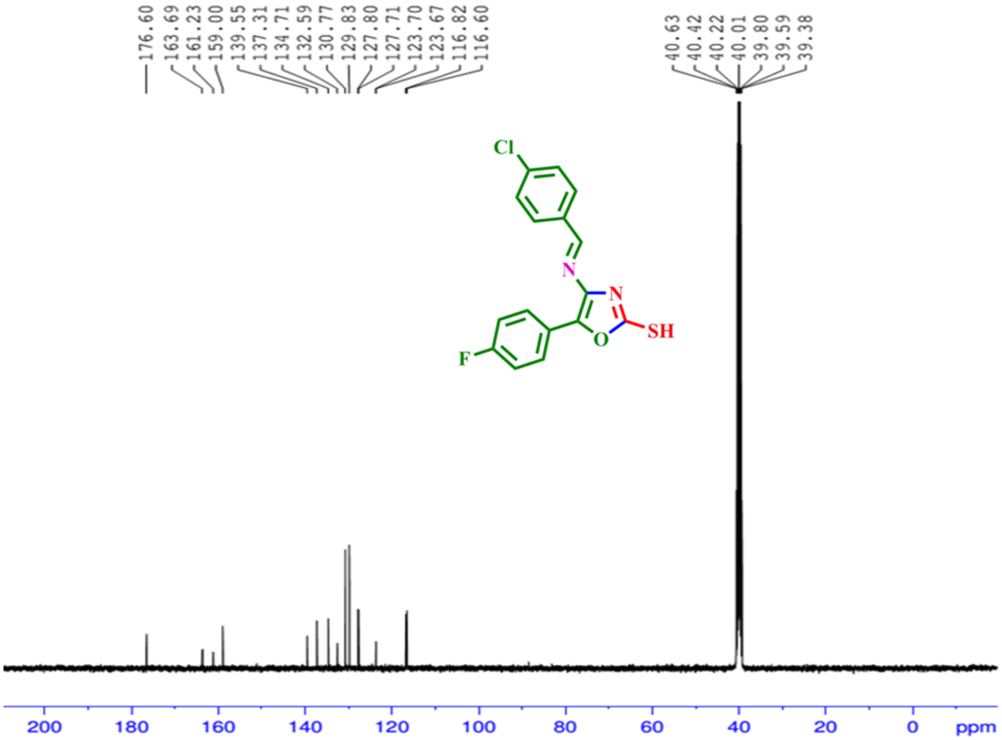

Figure S30. ^{13}C NMR spectrum of **3m**.

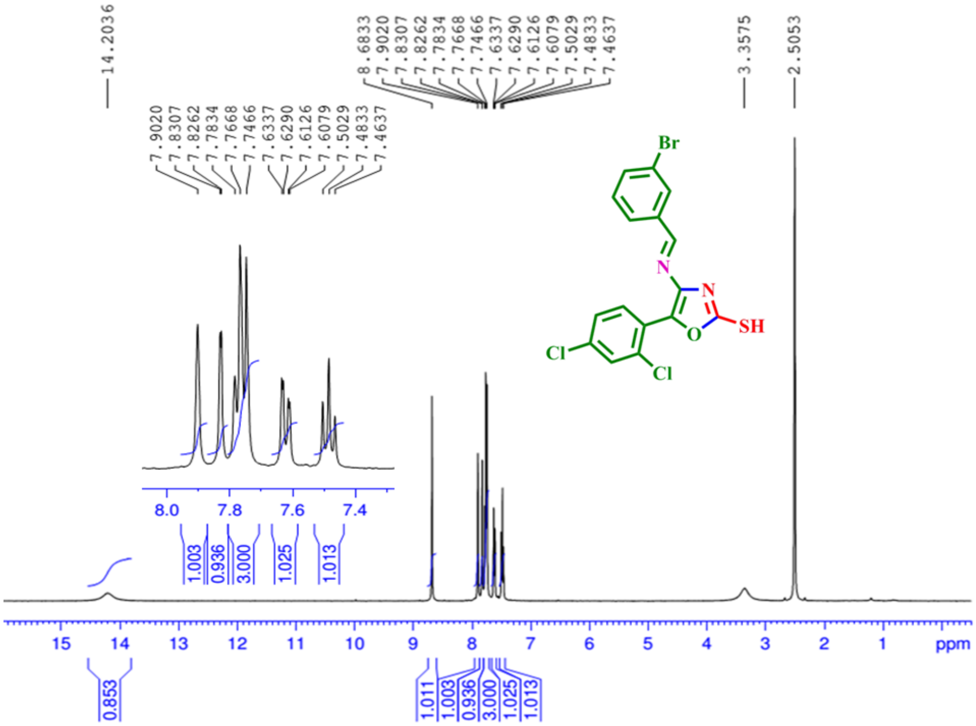

Figure S31. ^{1}H NMR spectrum of **3n**.

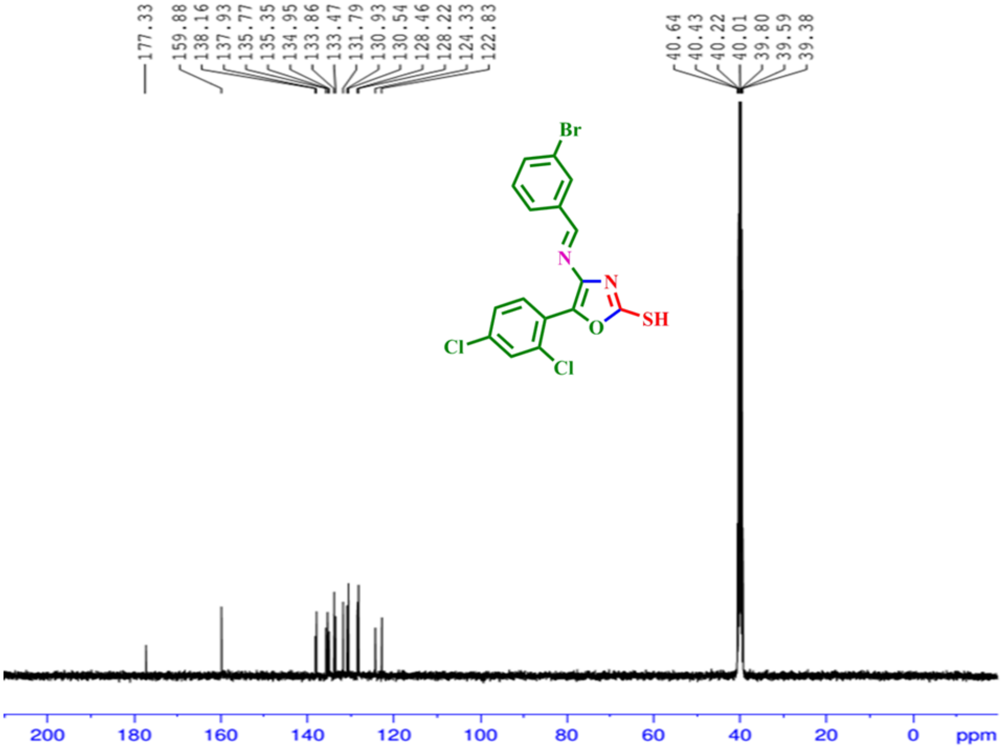

Figure S32. ^{13}C NMR spectrum of **3n**.

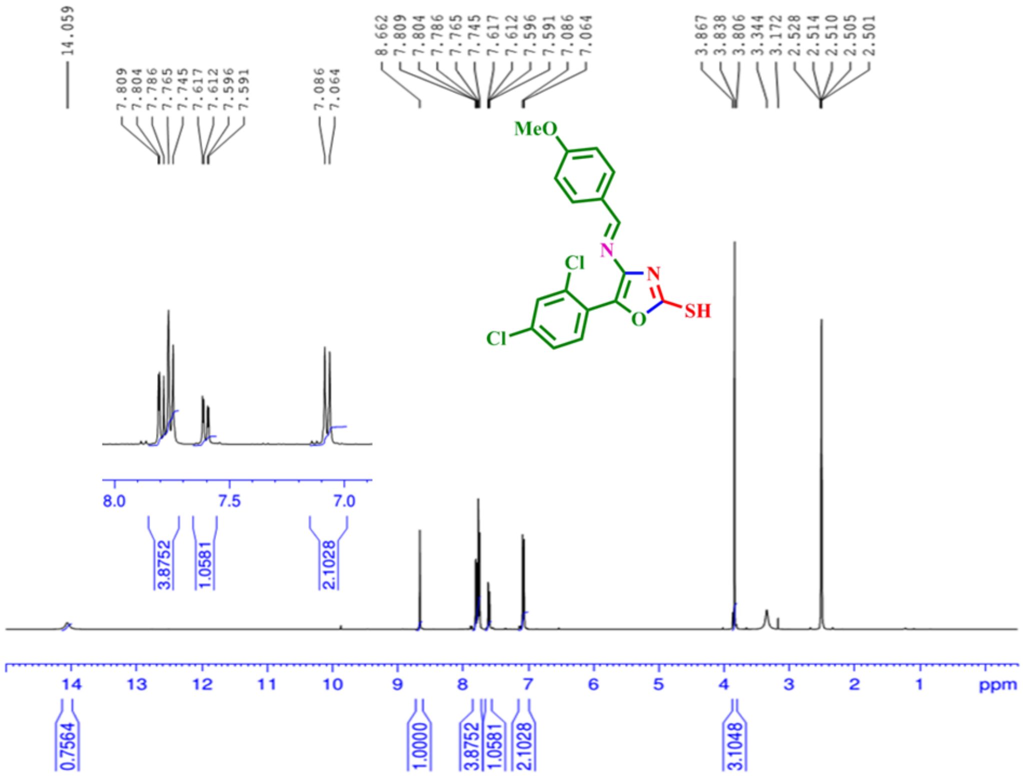

Figure S33. ^{1}H NMR spectrum of **3o**.

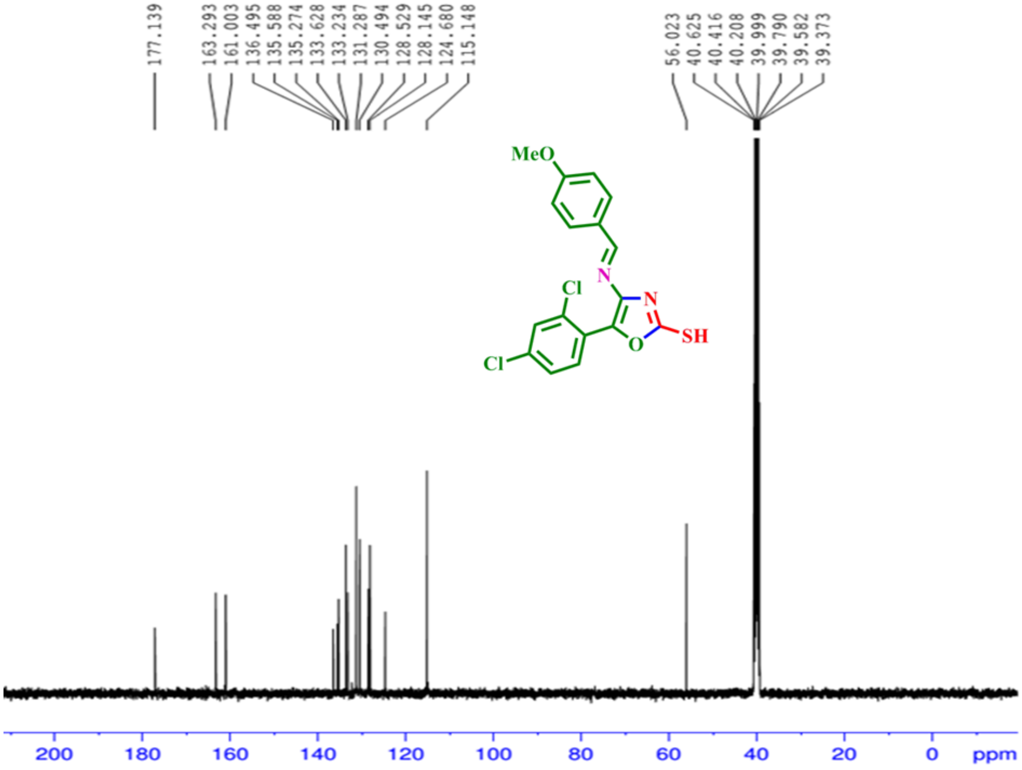

Figure S34. ^{13}C NMR spectrum of **3o**.

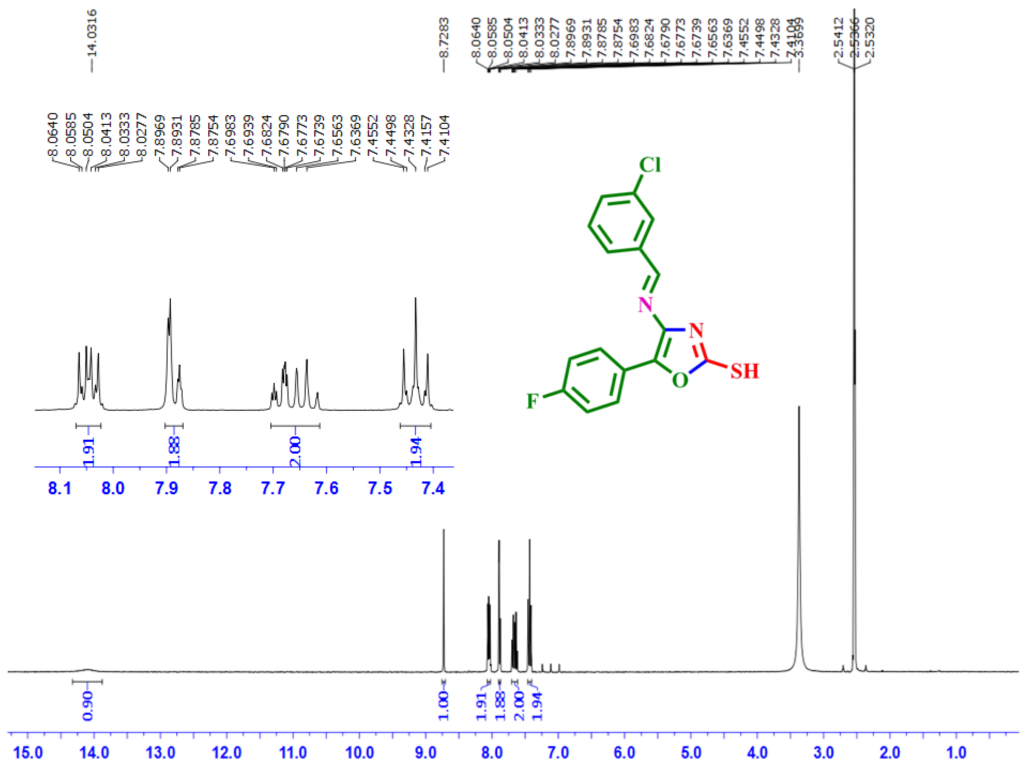

Figure S35. ^{1}H NMR spectrum of **3p**.

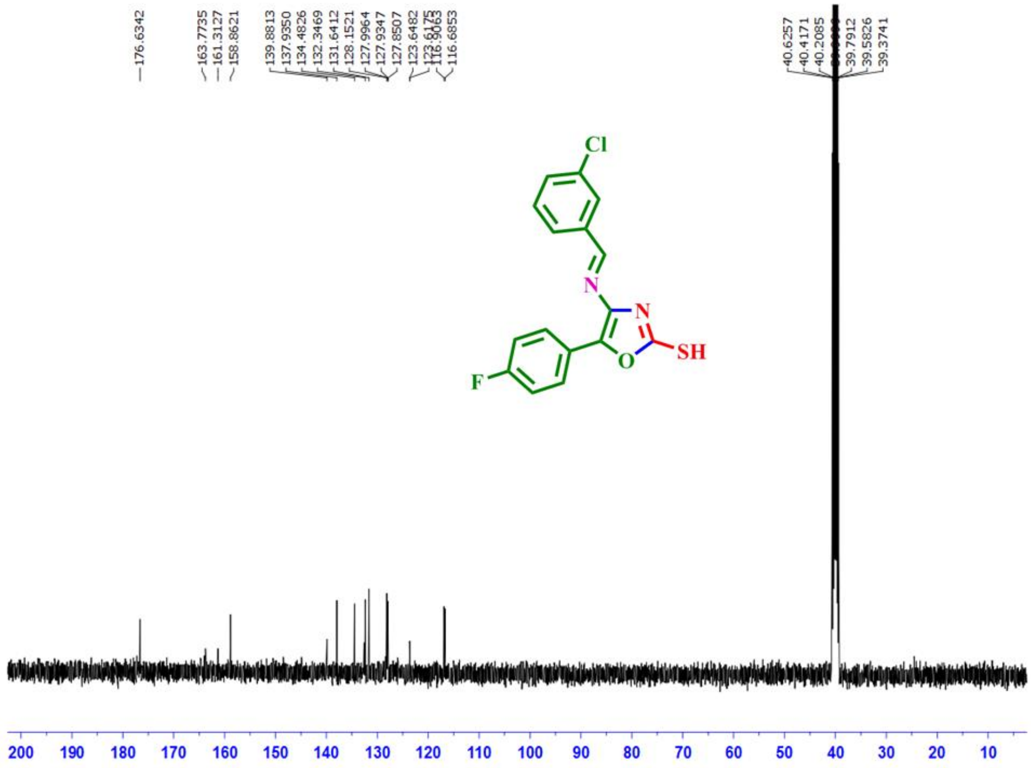

Figure S36. ^{13}C NMR spectrum of **3p**.

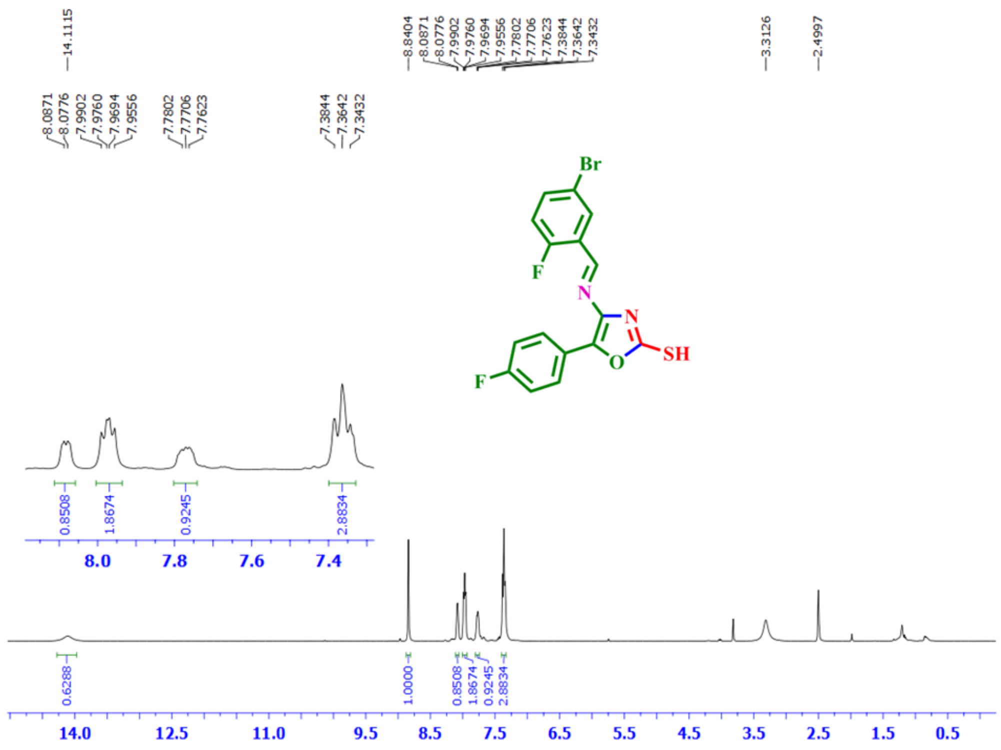

Figure S37. ^{1}H NMR spectrum of **3q**.

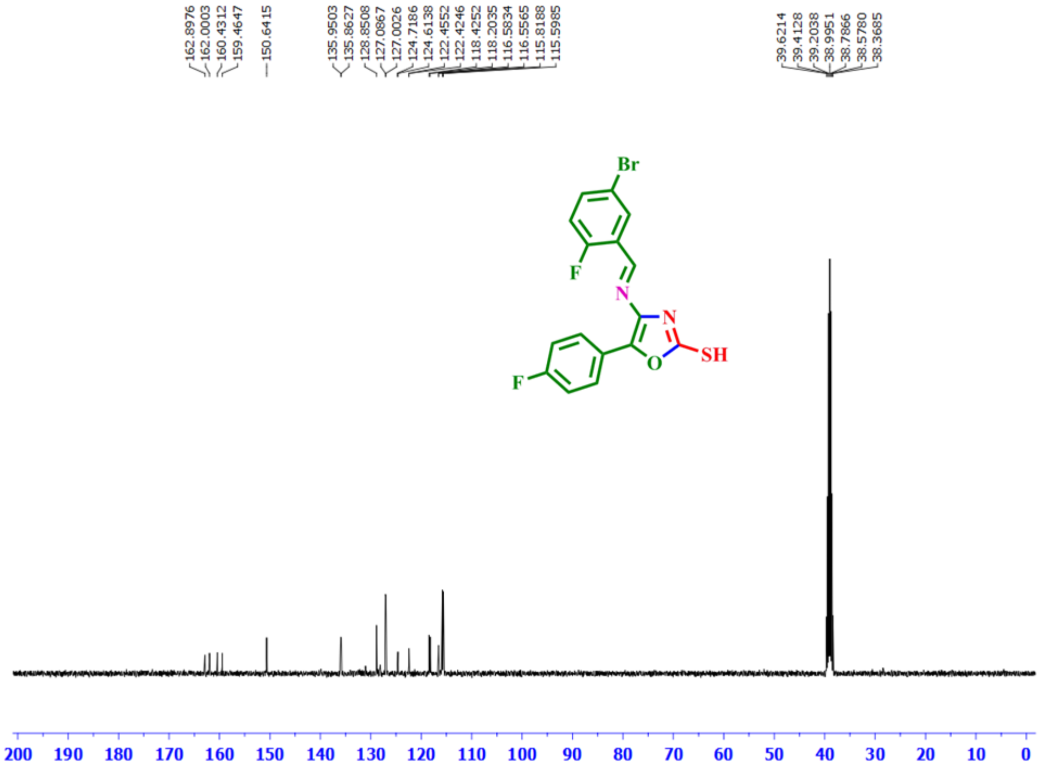

Figure S38. ^{13}C NMR spectrum of **3q**.

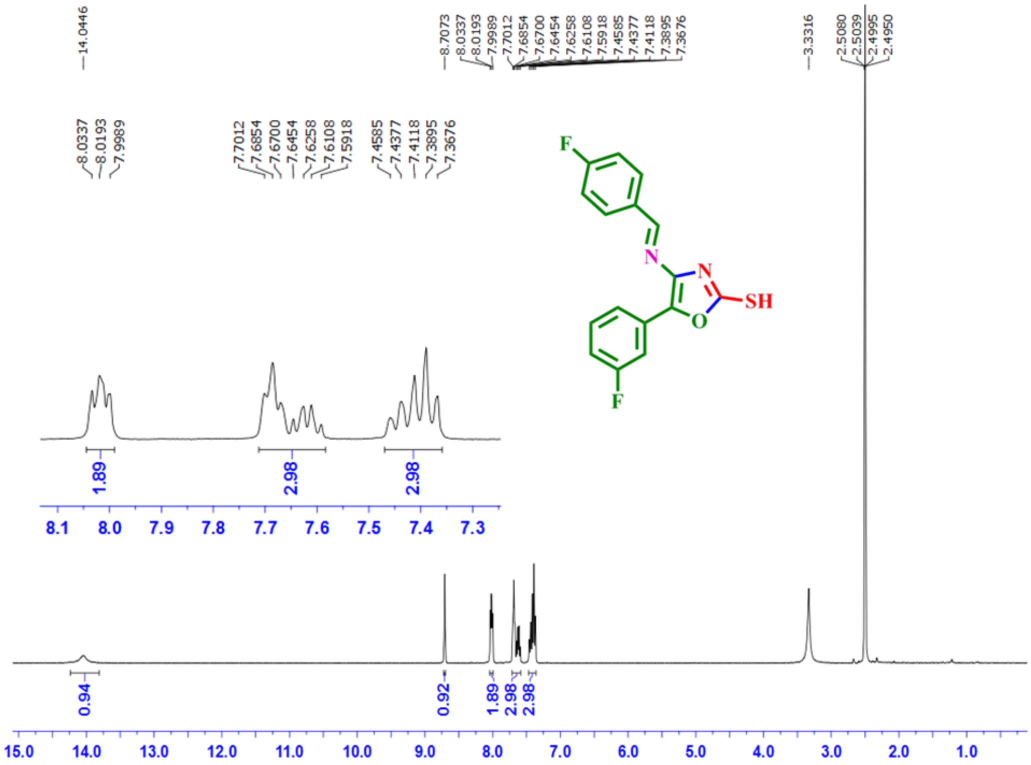

Figure S39. ^{1}H NMR spectrum of **3r**.

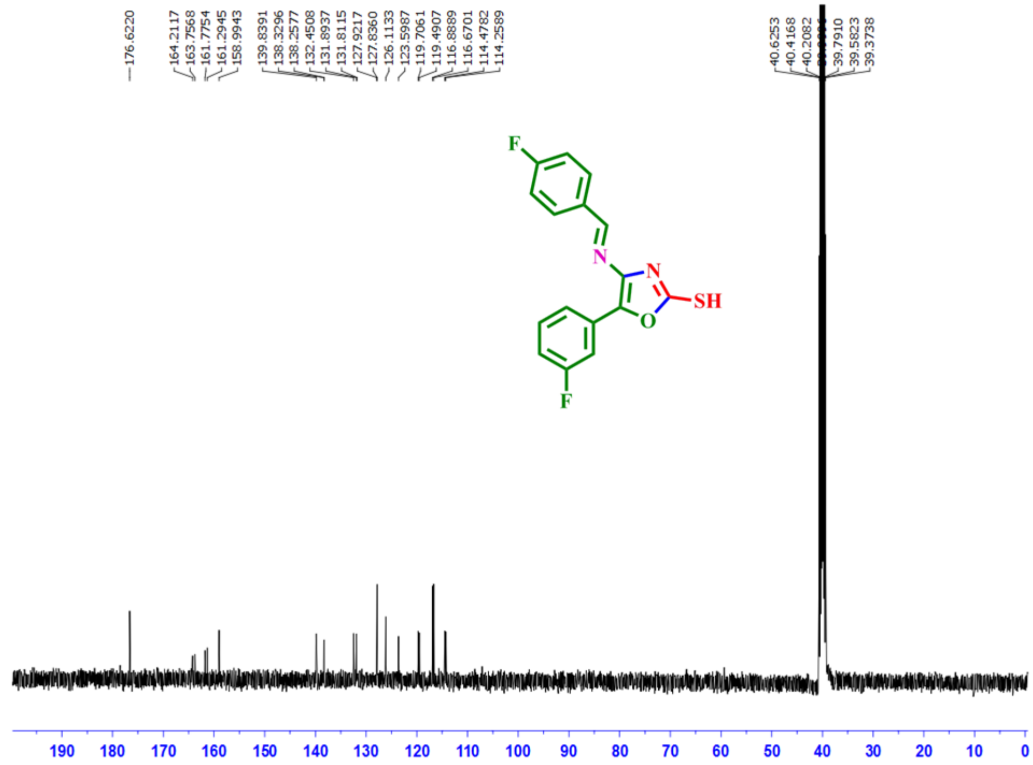

Figure S40. ^{13}C NMR spectrum of **3r**.

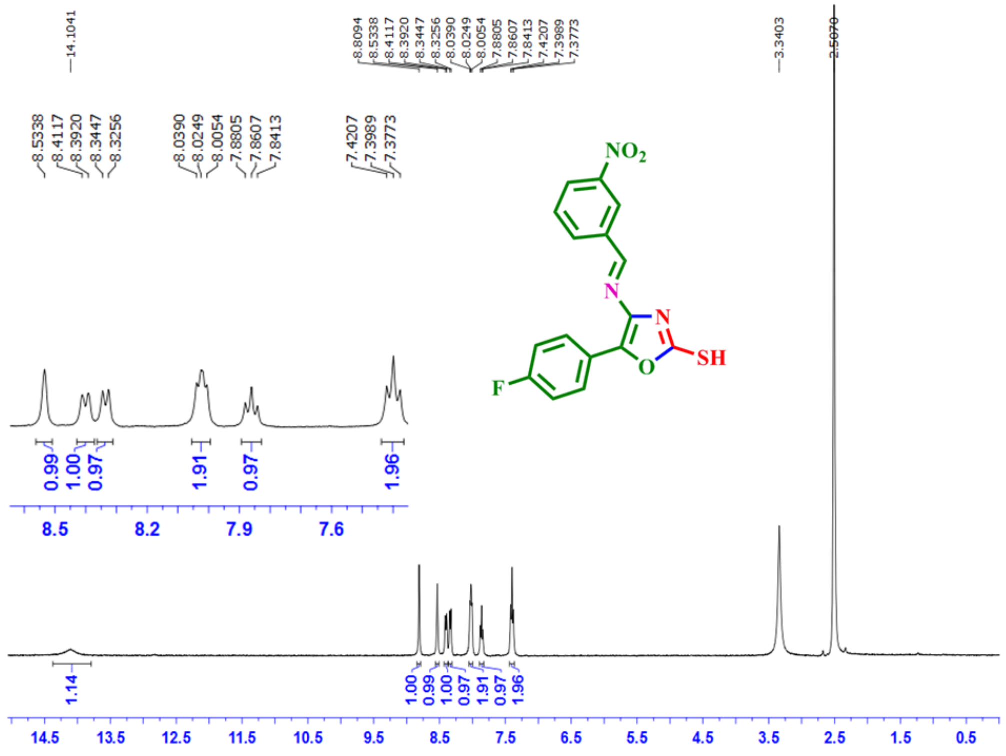

Figure S41. ^{1}H NMR spectrum of **3s**.

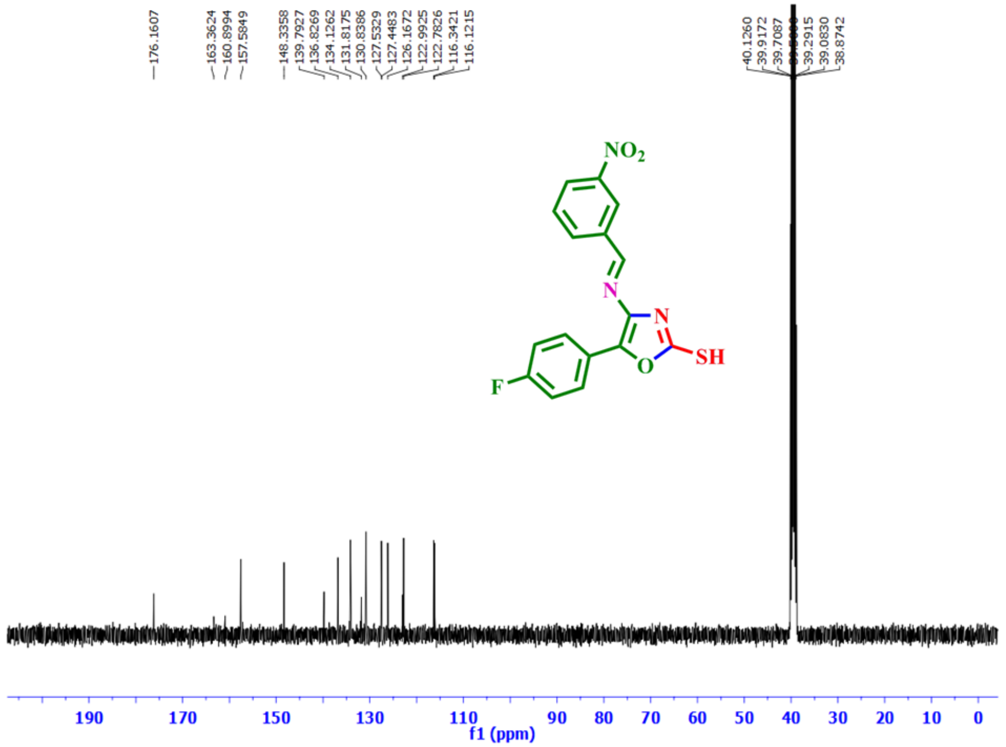

Figure S42. ^{13}C NMR spectrum of **3s**.

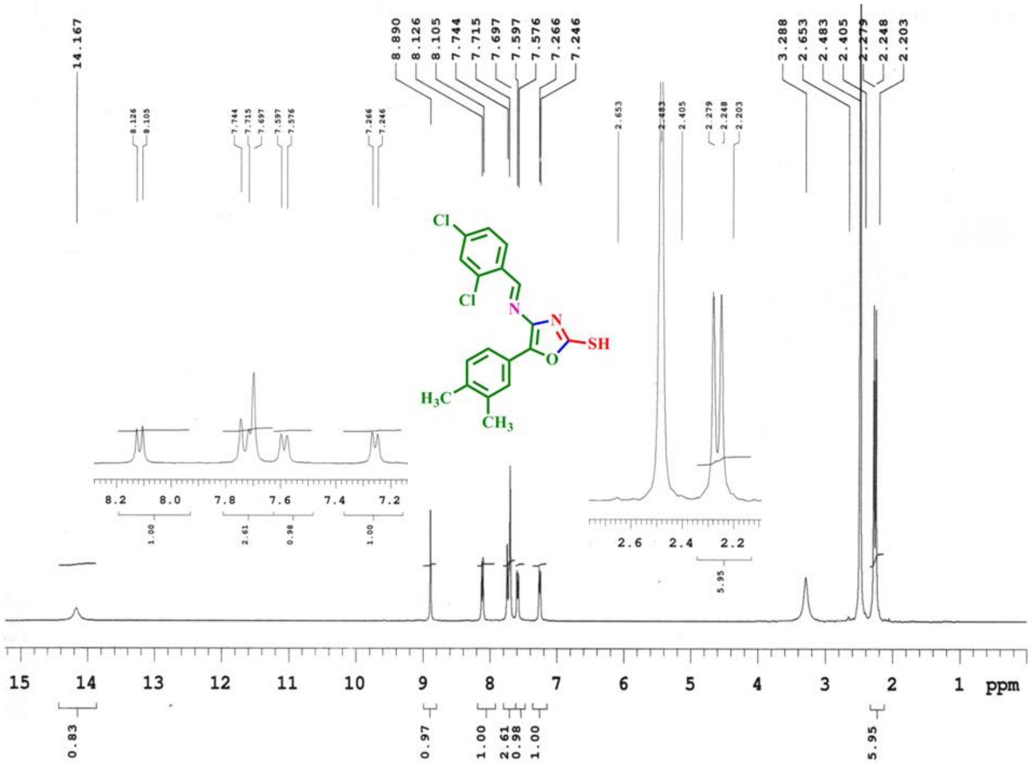

Figure S43. ^{1}H NMR spectrum of **3t**.

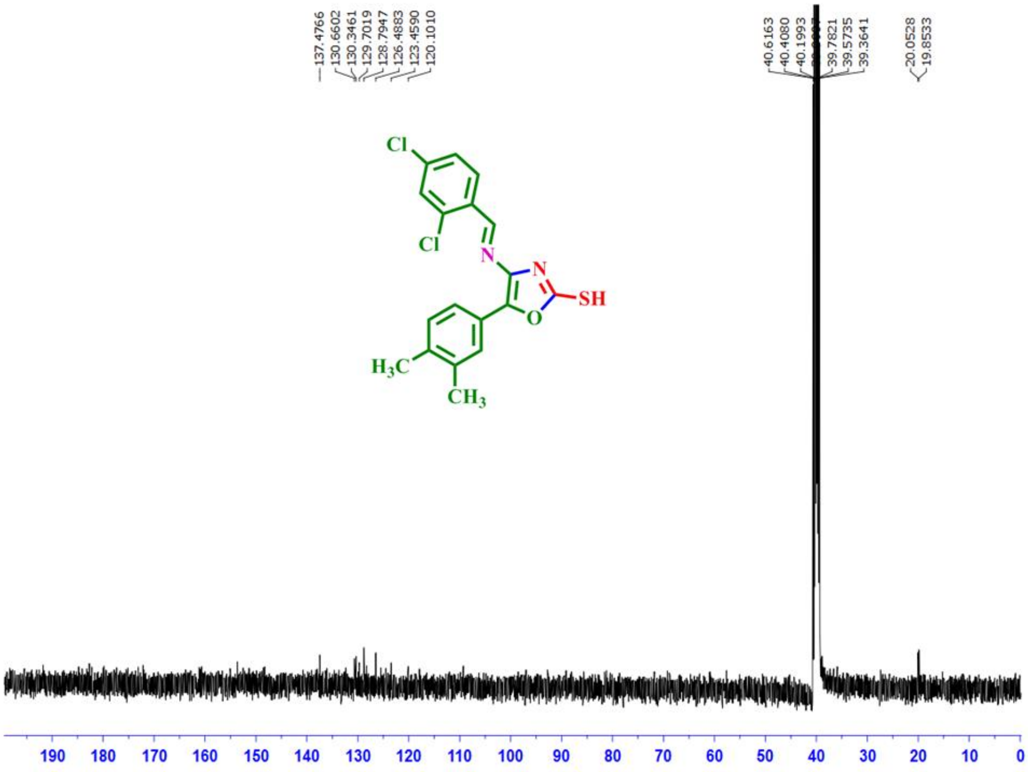

Figure S44. ^{13}C NMR spectrum of **3t**.

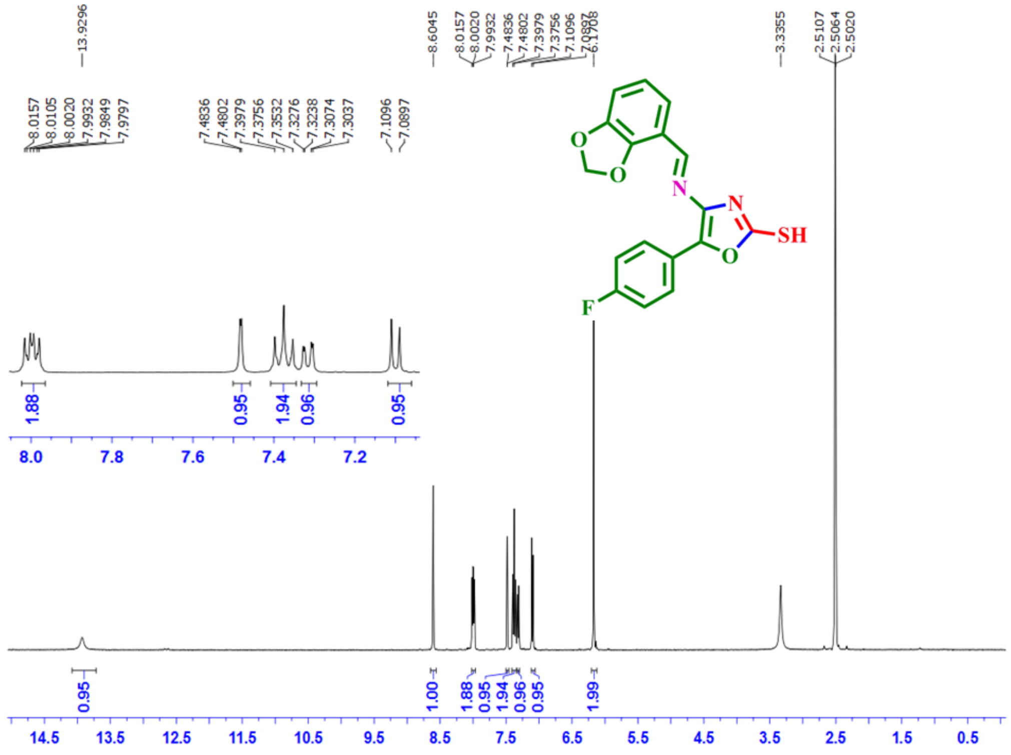

Figure S45. ^{1}H NMR spectrum of **3u**.

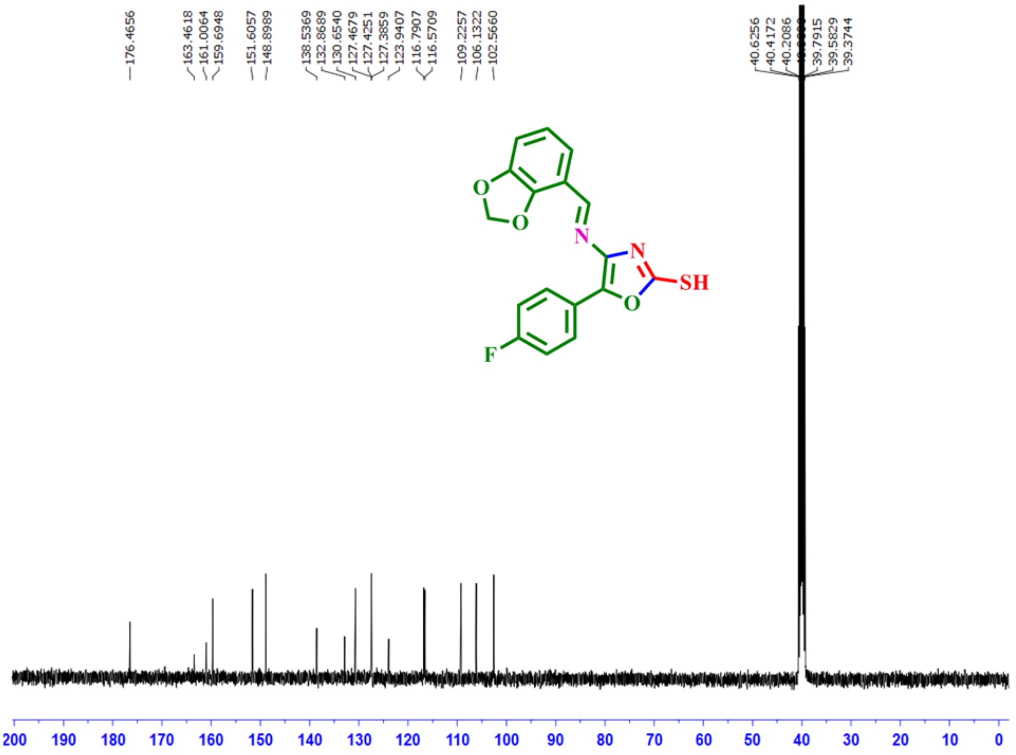

Figure S46. ^{13}C NMR spectrum of **3u**.

VI Copies of NMR spectra of 5,6 and 7

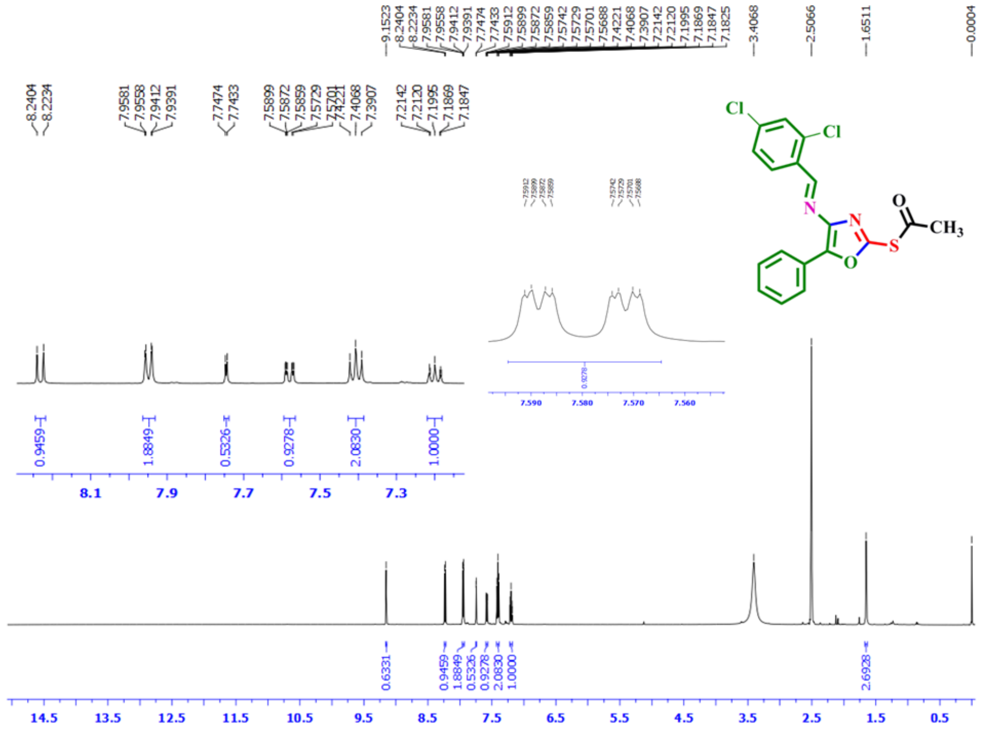

Figure S47. ^{1}H NMR spectrum of **5**.

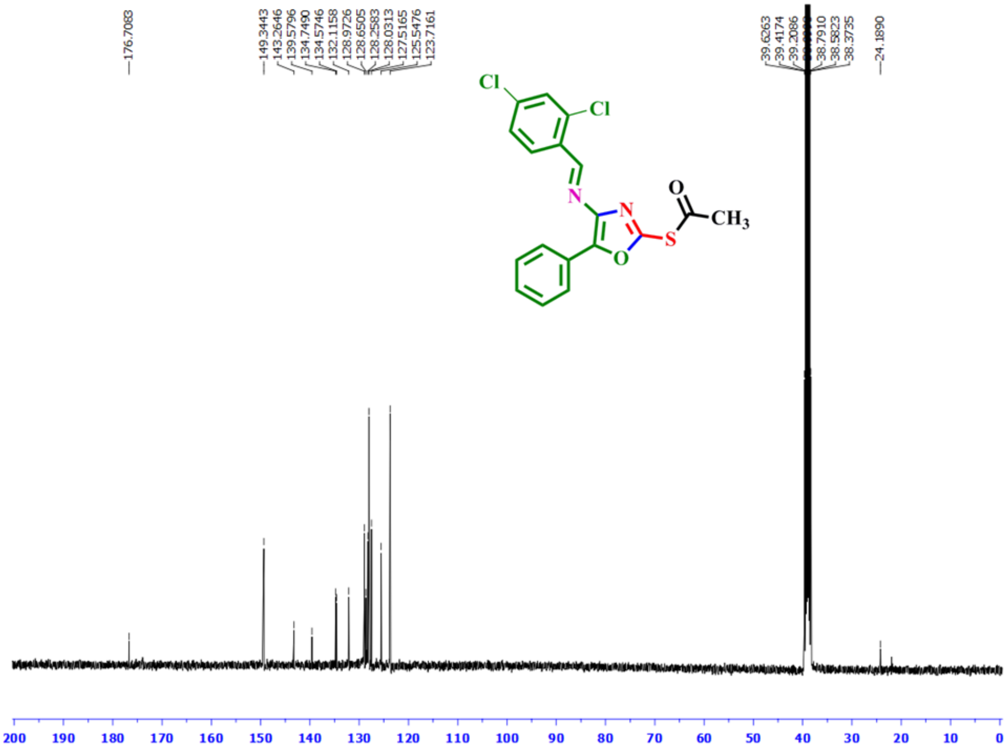

Figure S48. ^{13}CNMR spectrum of **5**.

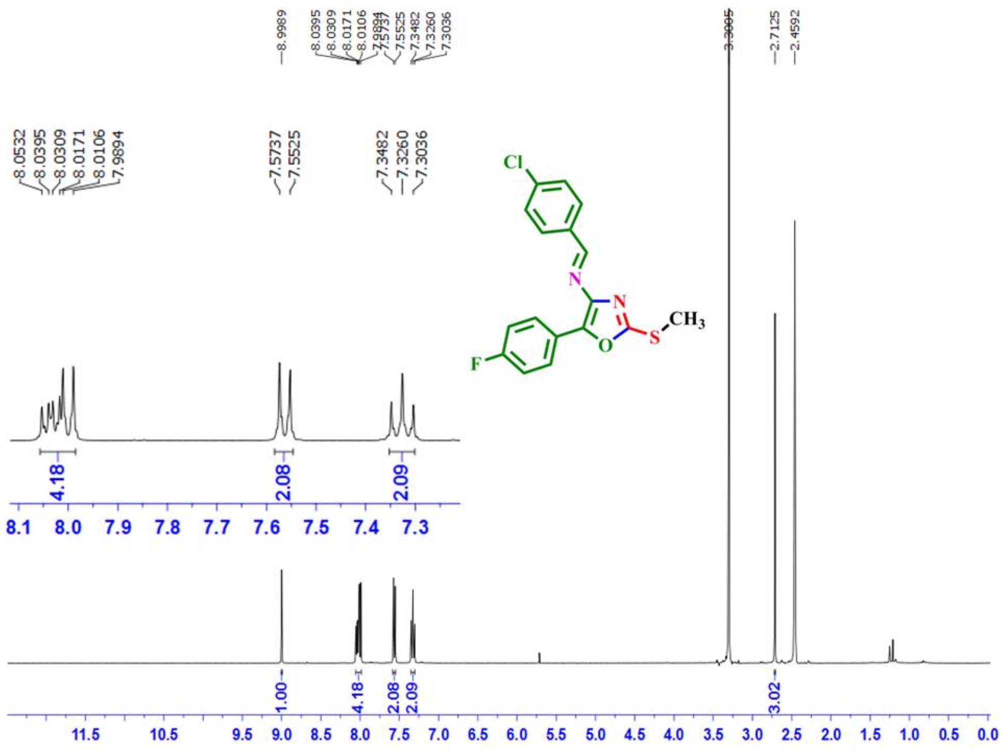

Figure S49. ^{1}H NMR spectrum of **6**.

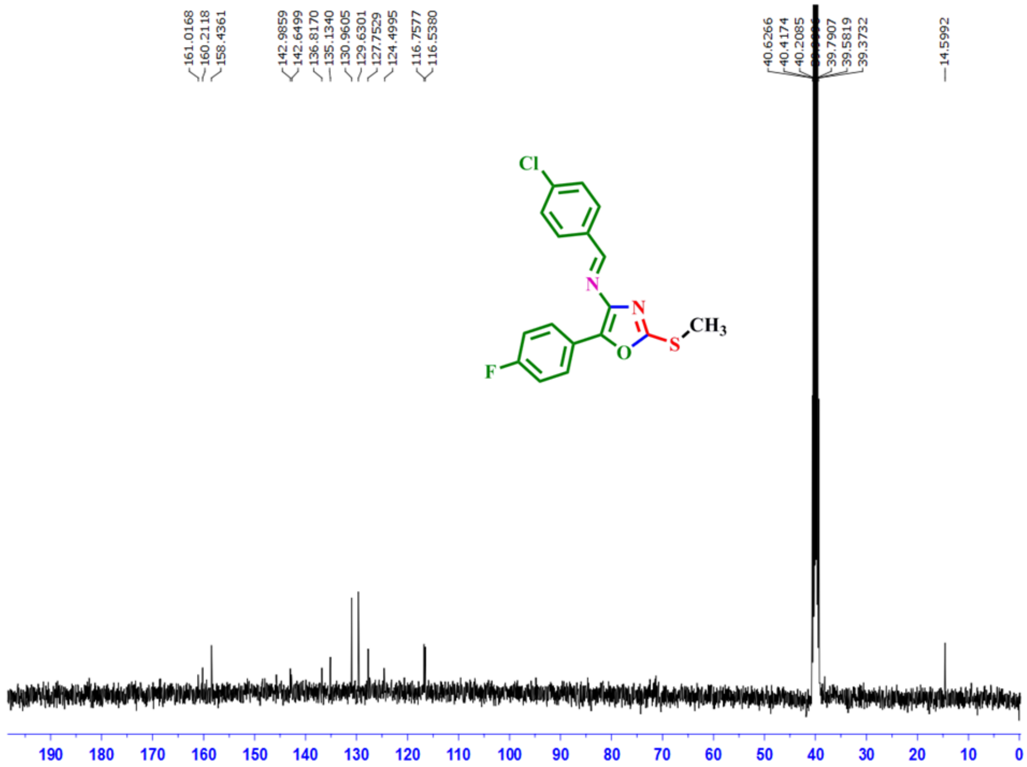

Figure S50. ^{13}C NMR spectrum of **6**.

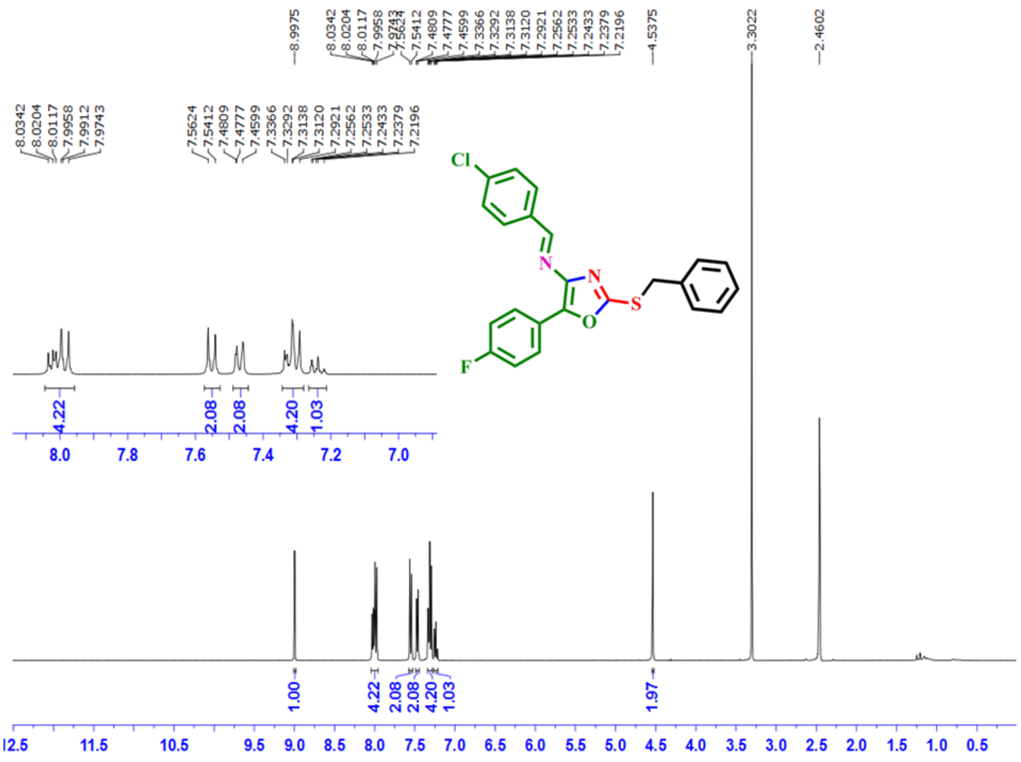

Figure S51. ^{1}H NMR spectrum of **7**.

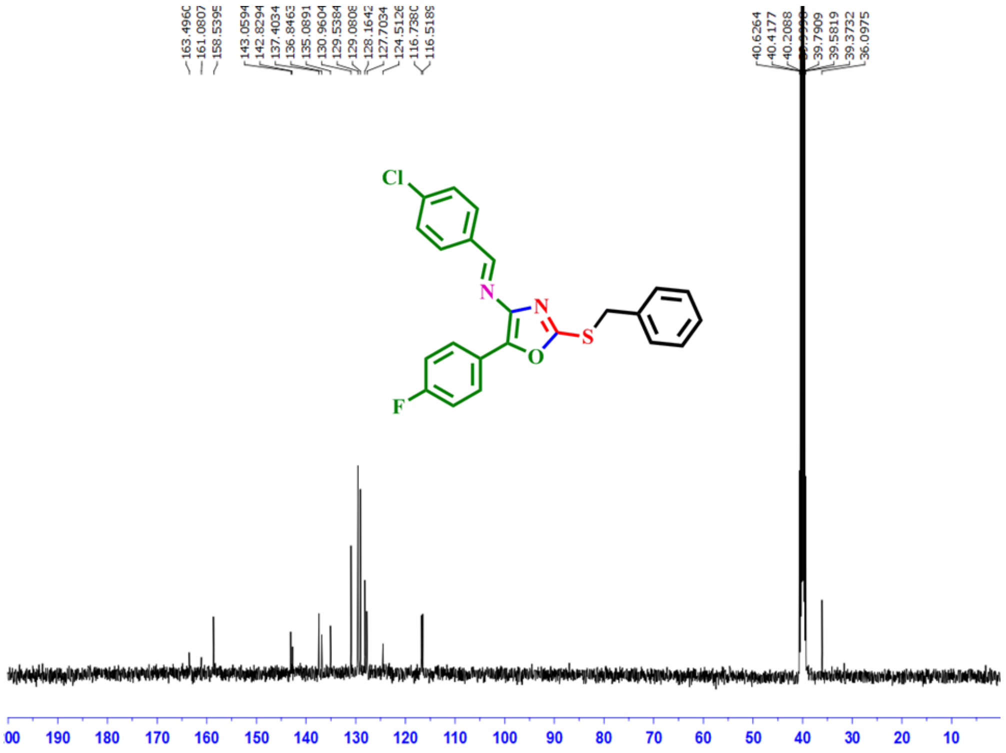

Figure S52. ^{13}C NMR spectrum of **7**.

VII Copies of NMR spectra of 4

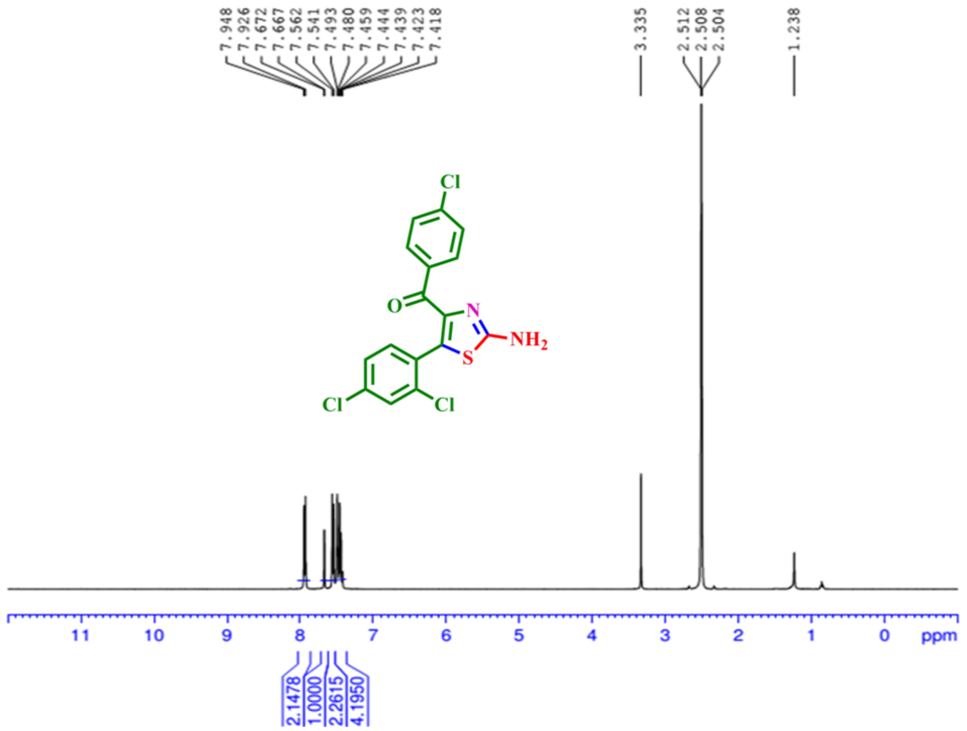

Figure S53. ^{1}H NMR spectrum of **4a**.

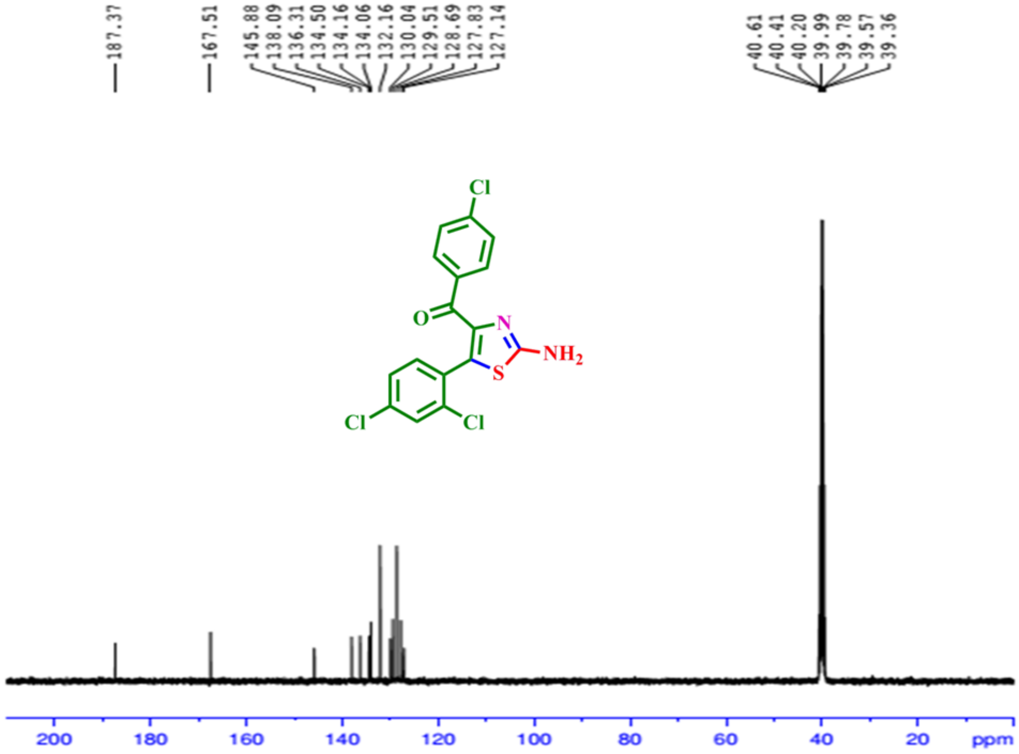

Figure S54. ^{13}C NMR spectrum of **4a**.

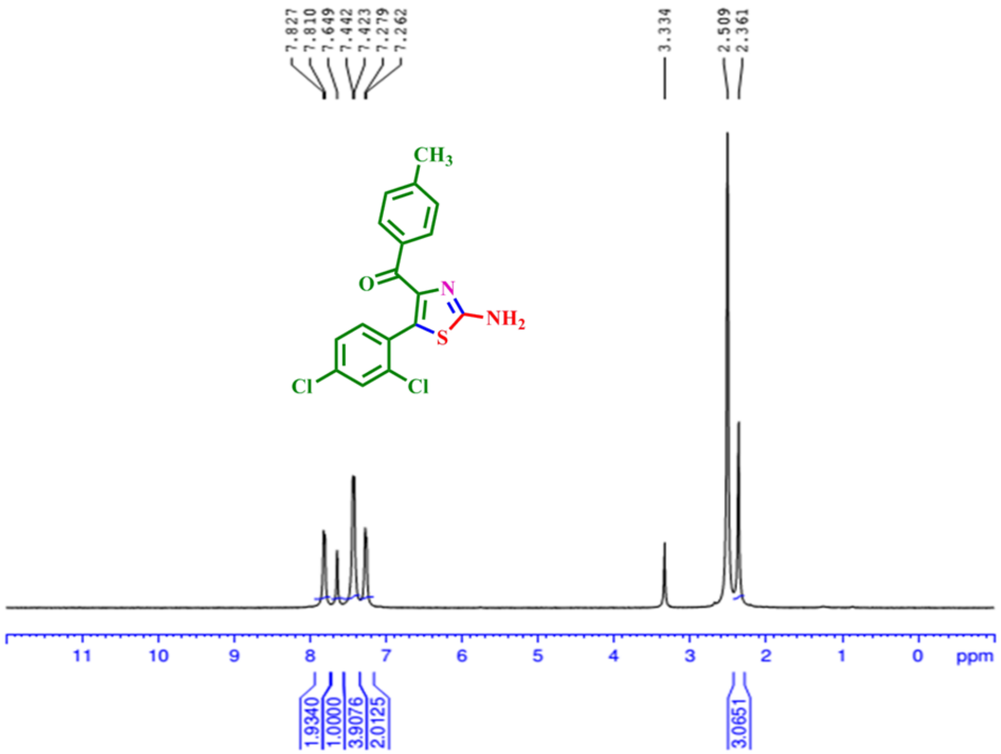

Figure S55. ^{1}H NMR spectrum of **4b**.

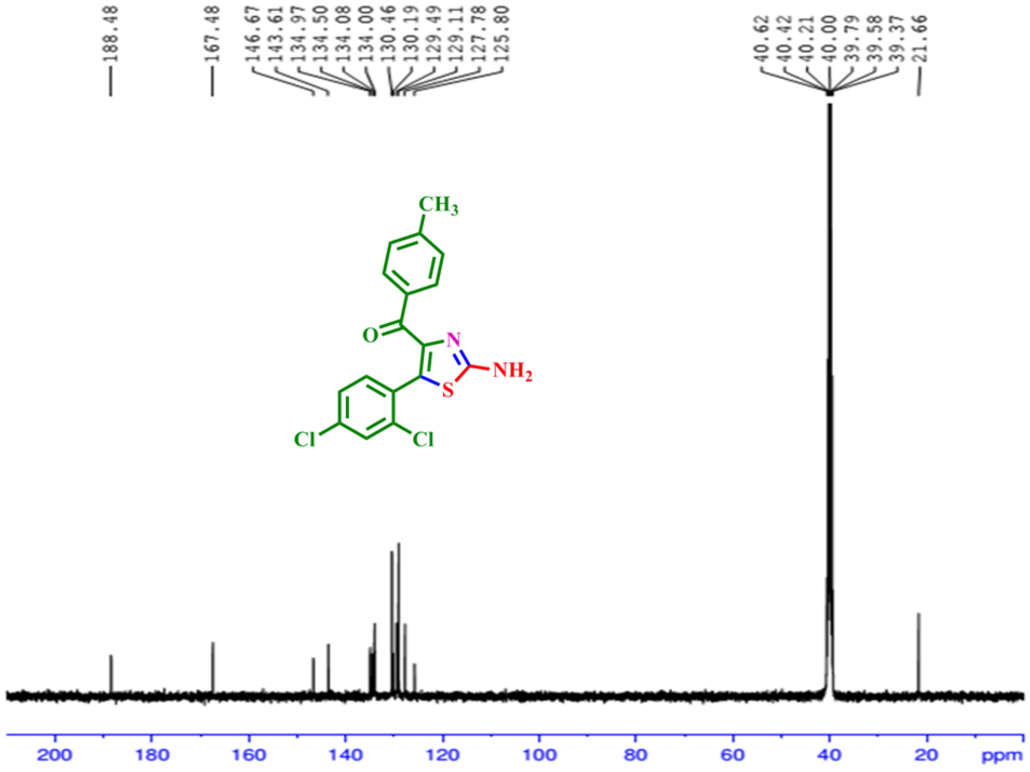

Figure S56. ^{13}C NMR spectrum of **4b**.

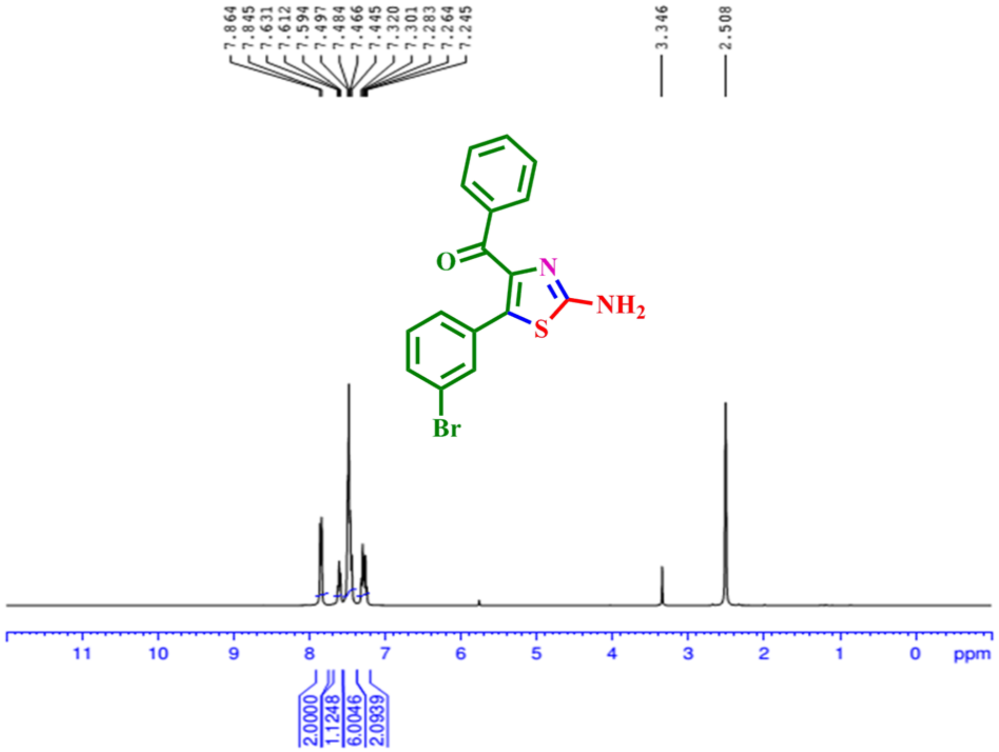

Figure S57. ^{1}H NMR spectrum of **4c**.

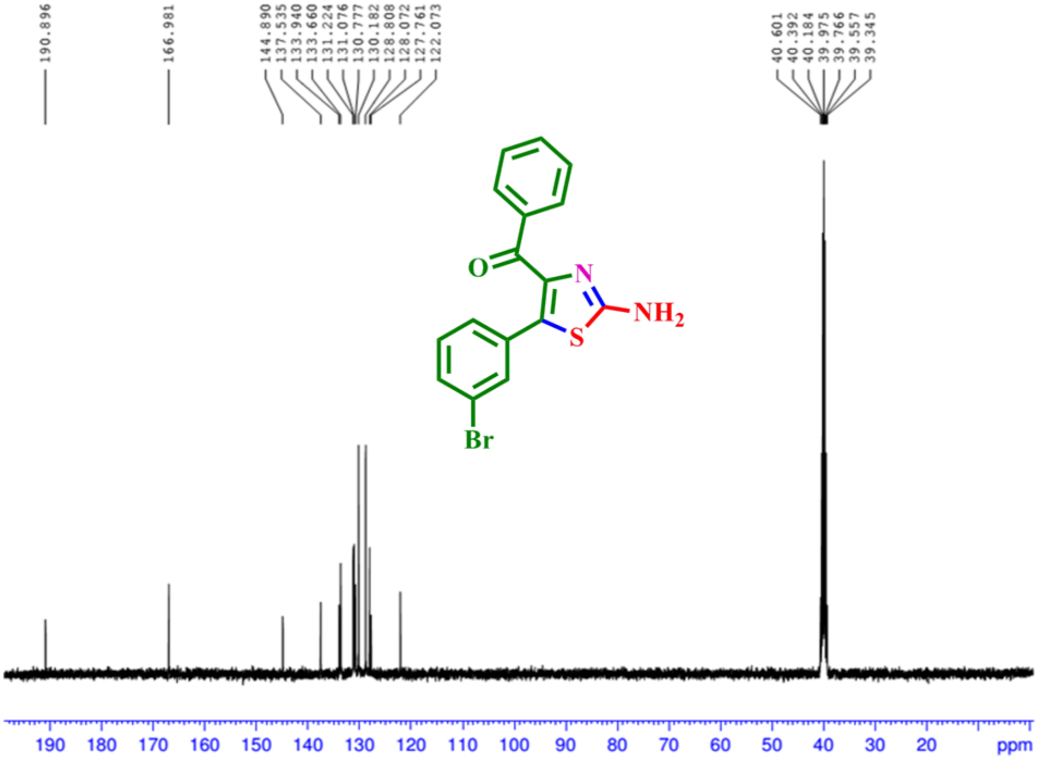

Figure S58. ^{13}C NMR spectrum of **4c**.

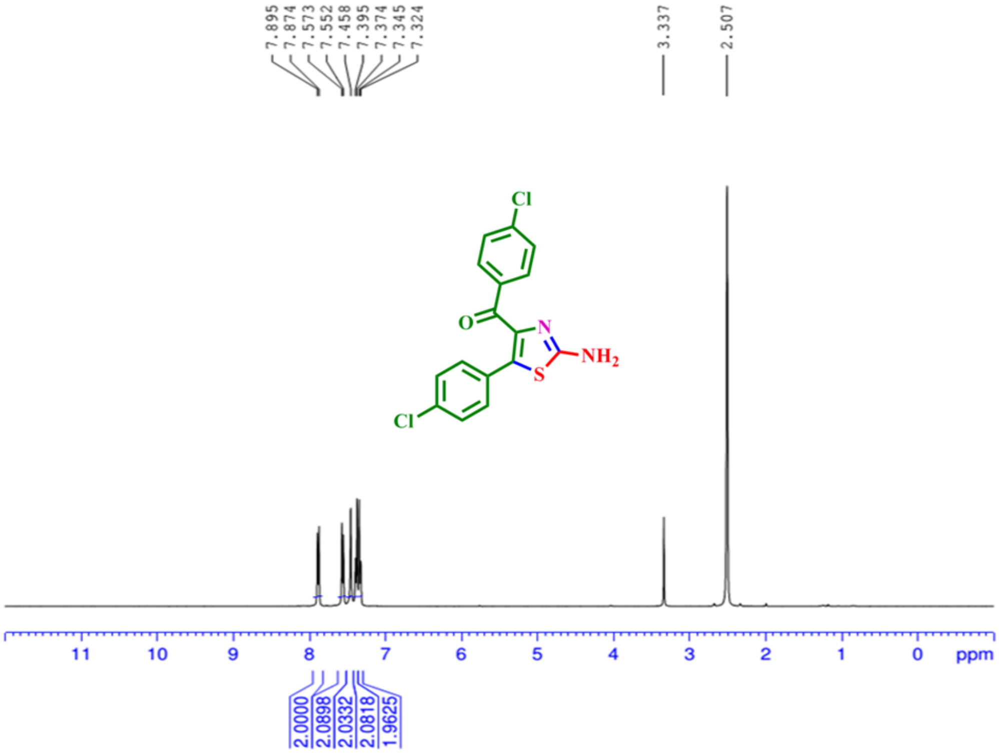

Figure S59. ^{1}H NMR spectrum of **4d**.

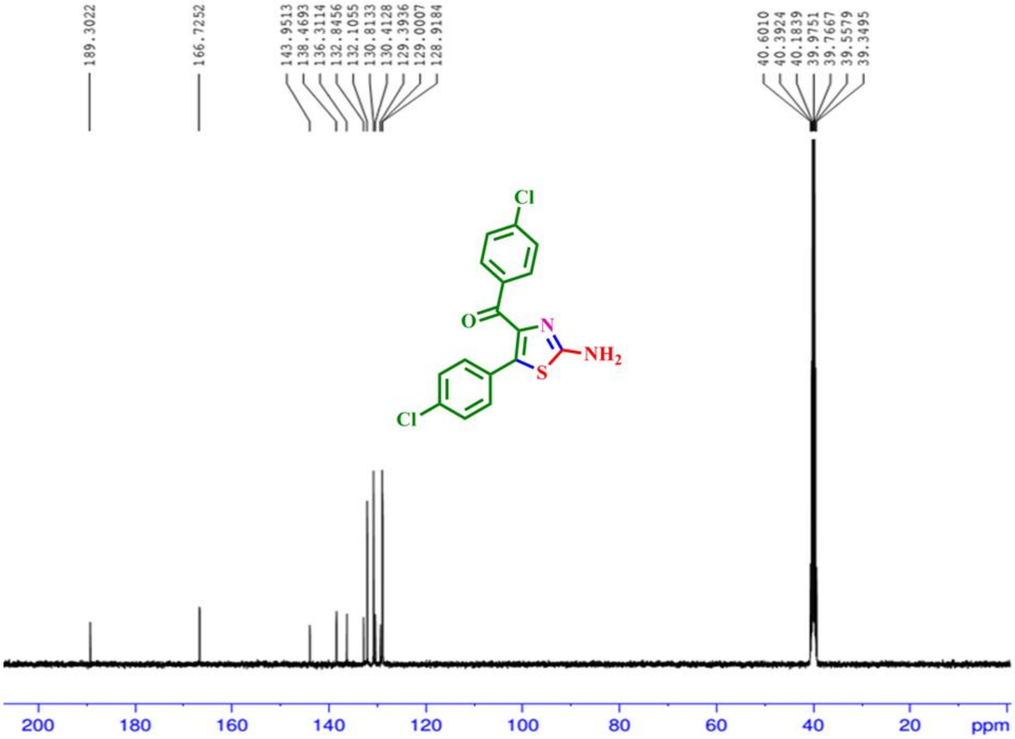

Figure S60. ^{13}C NMR spectrum of **4d**.

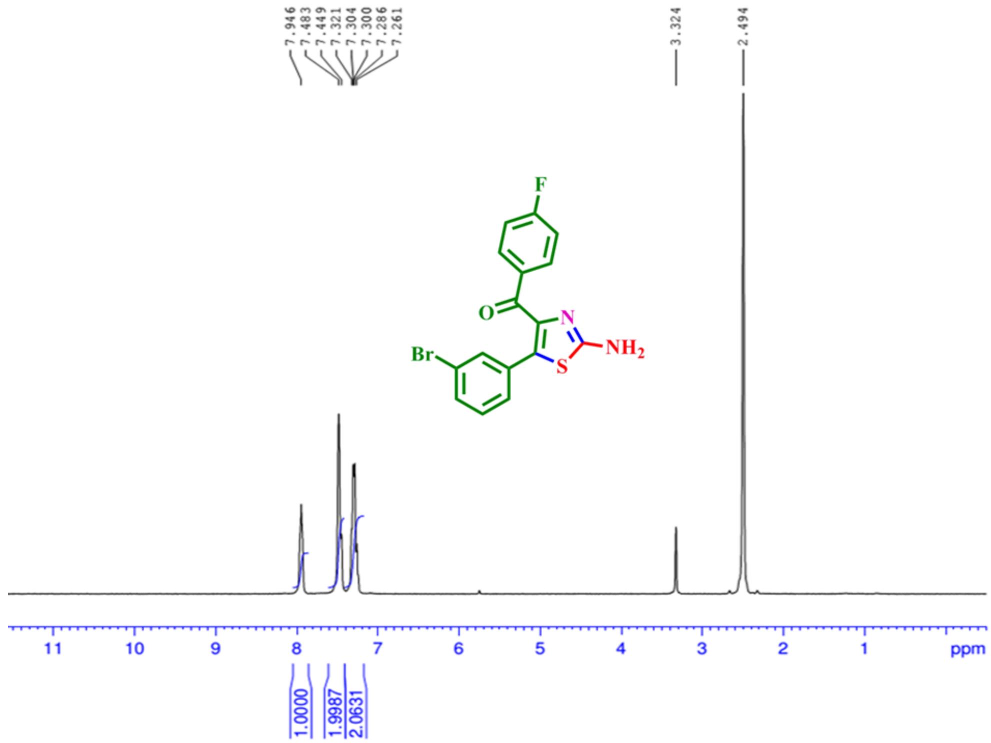

Figure S61. ^{1}H NMR spectrum of **4e**.

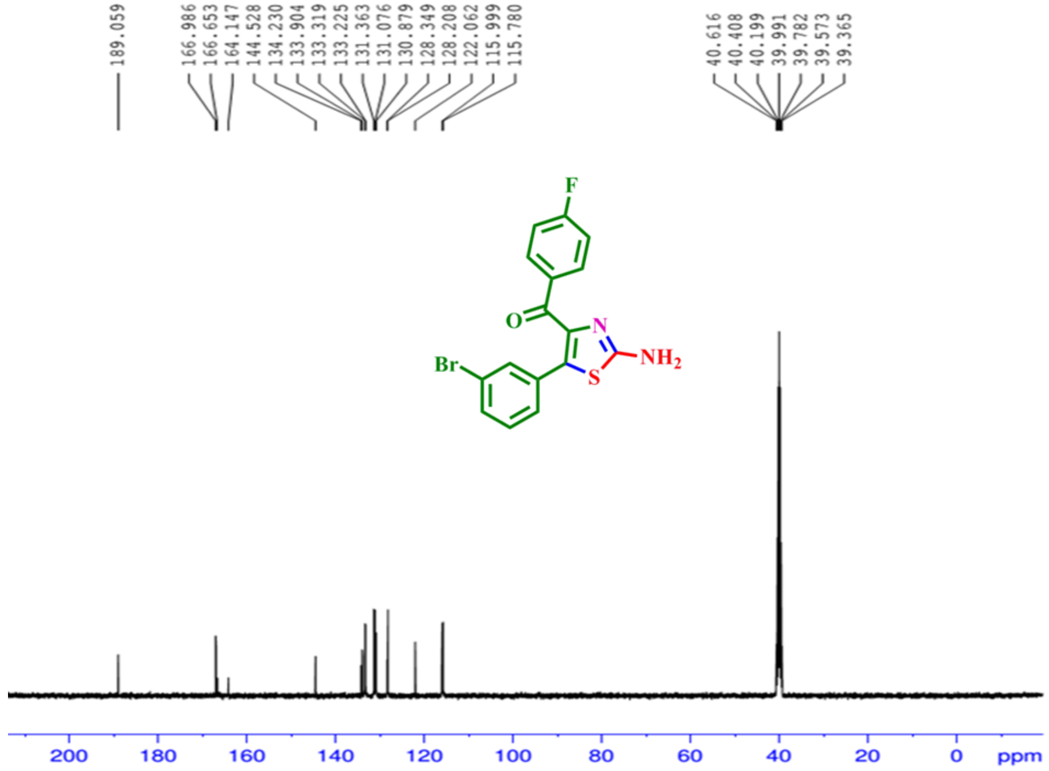

Figure S62. ^{13}C NMR spectrum of **4e**.

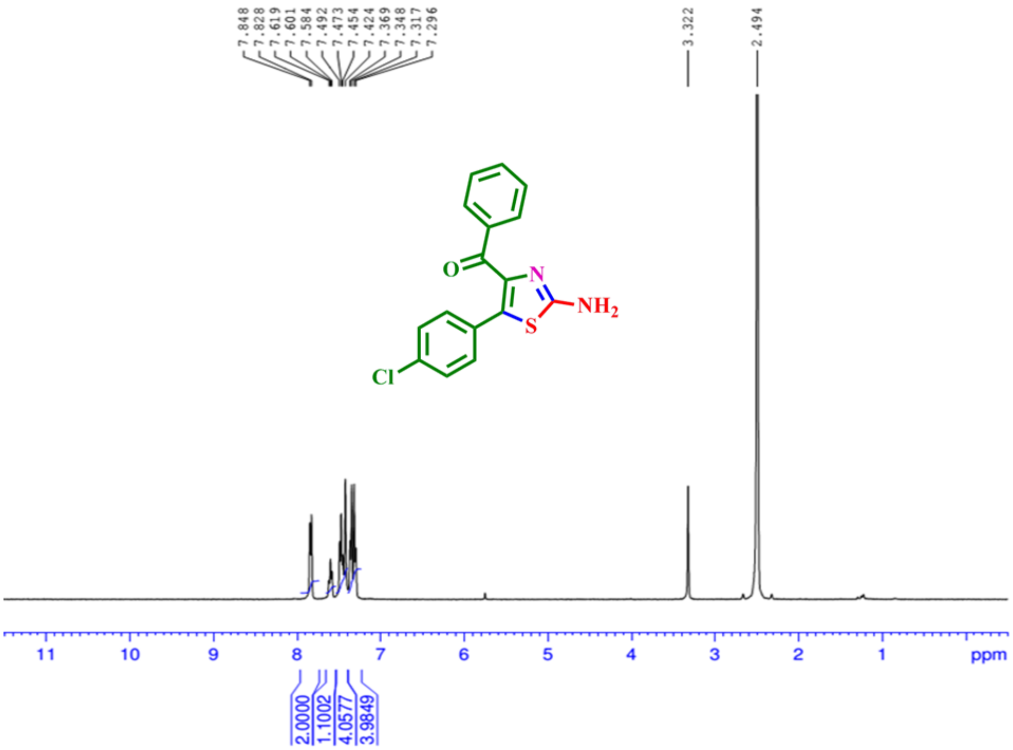

Figure S63. ^{1}H NMR spectrum of **4f**.

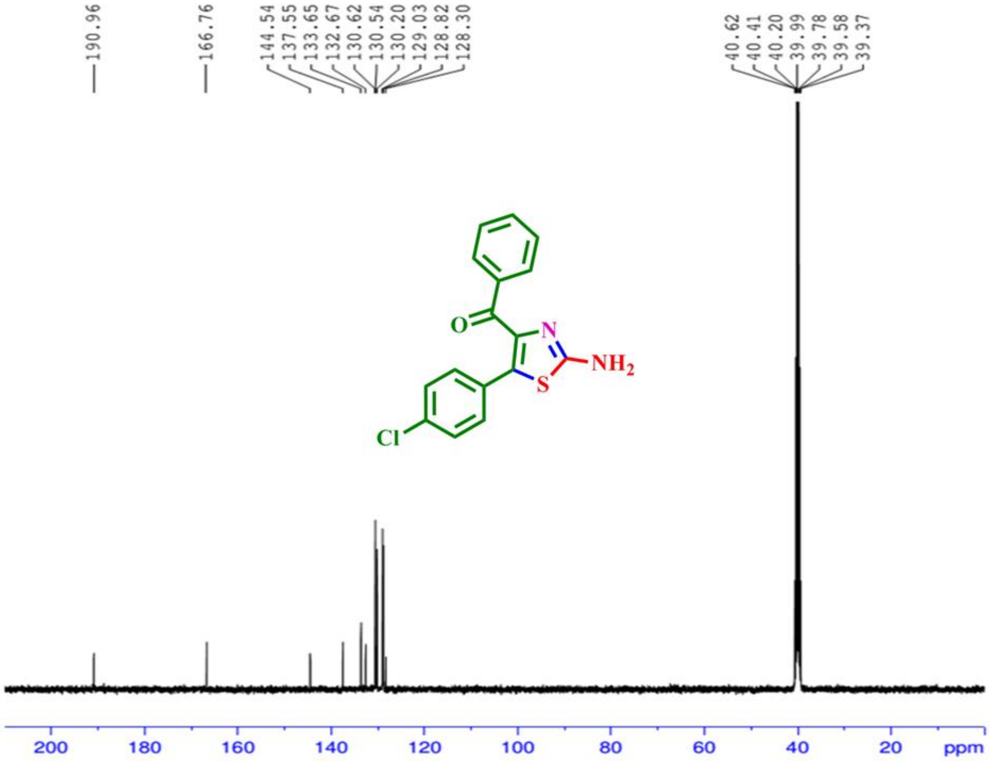

Figure S64. ^{13}C NMR spectrum of **4f**.

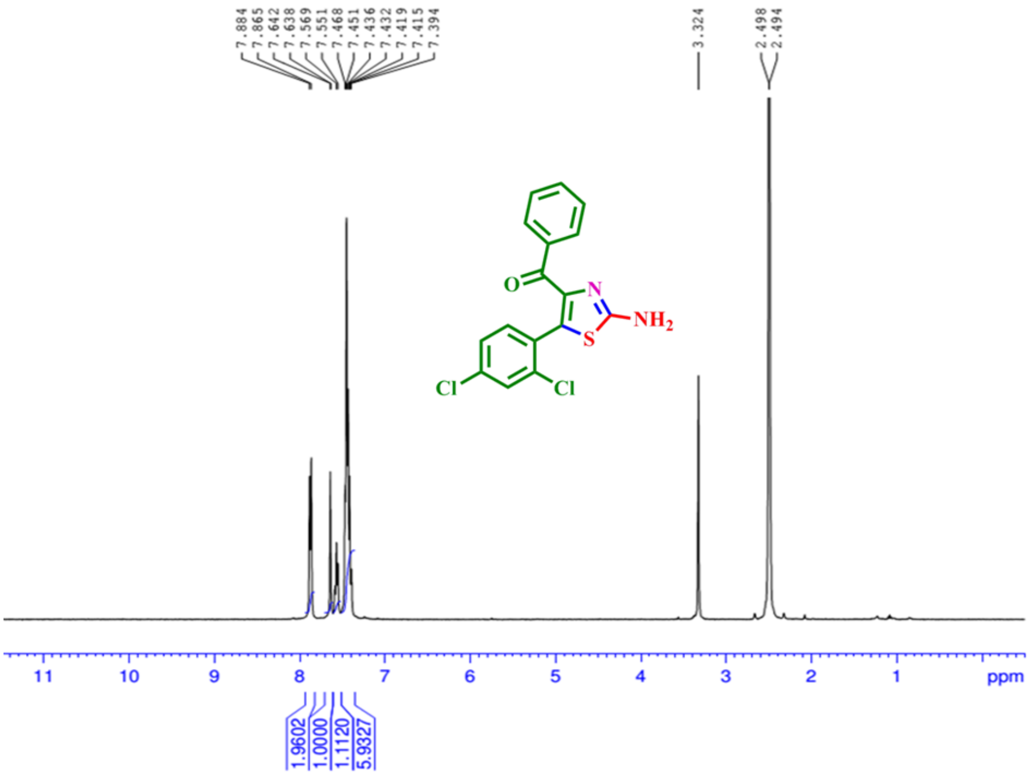

Figure S65. ^{1}H NMR spectrum of **4g**.

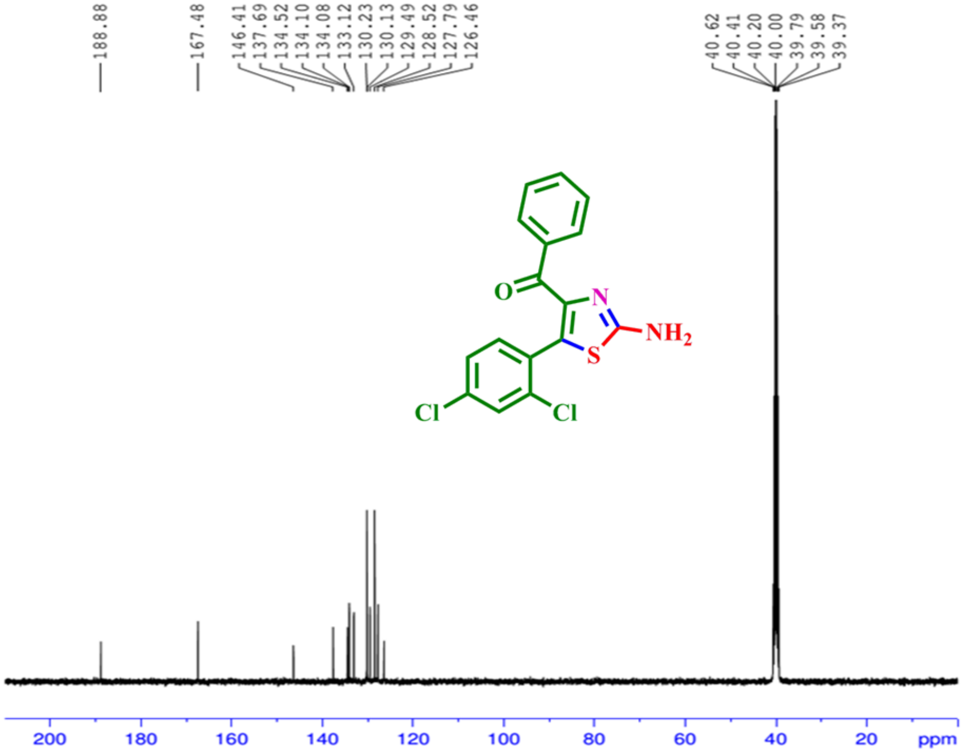

Figure S66. ^{13}C NMR spectrum of **4g**.

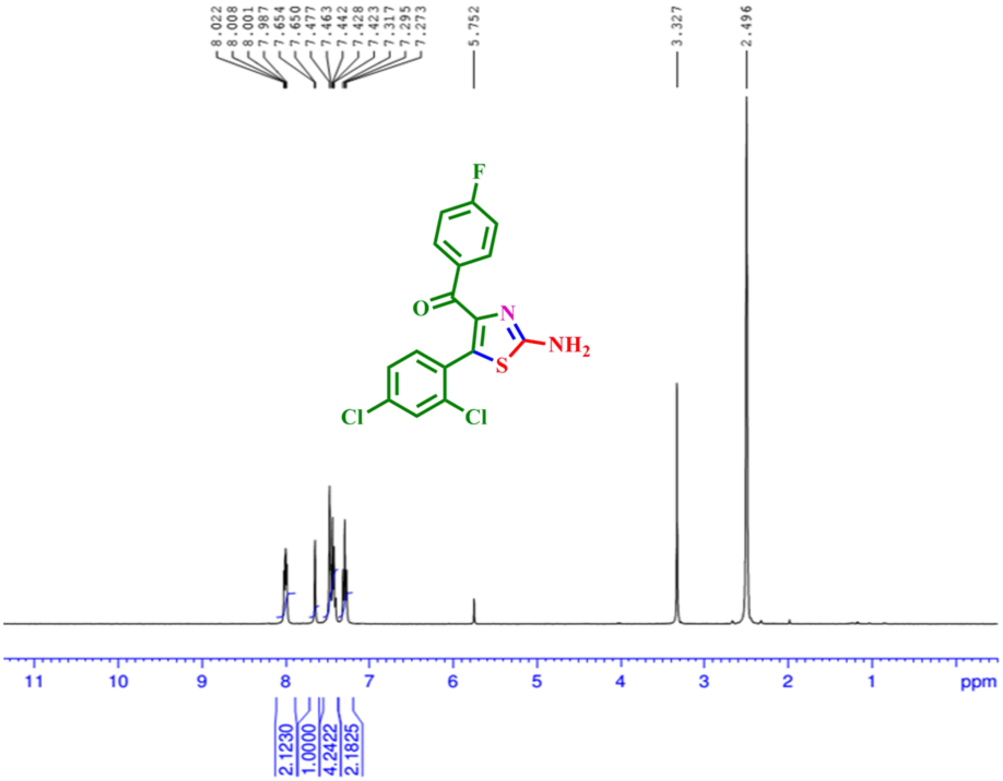

Figure S67. ^{1}H NMR spectrum of **4h**.

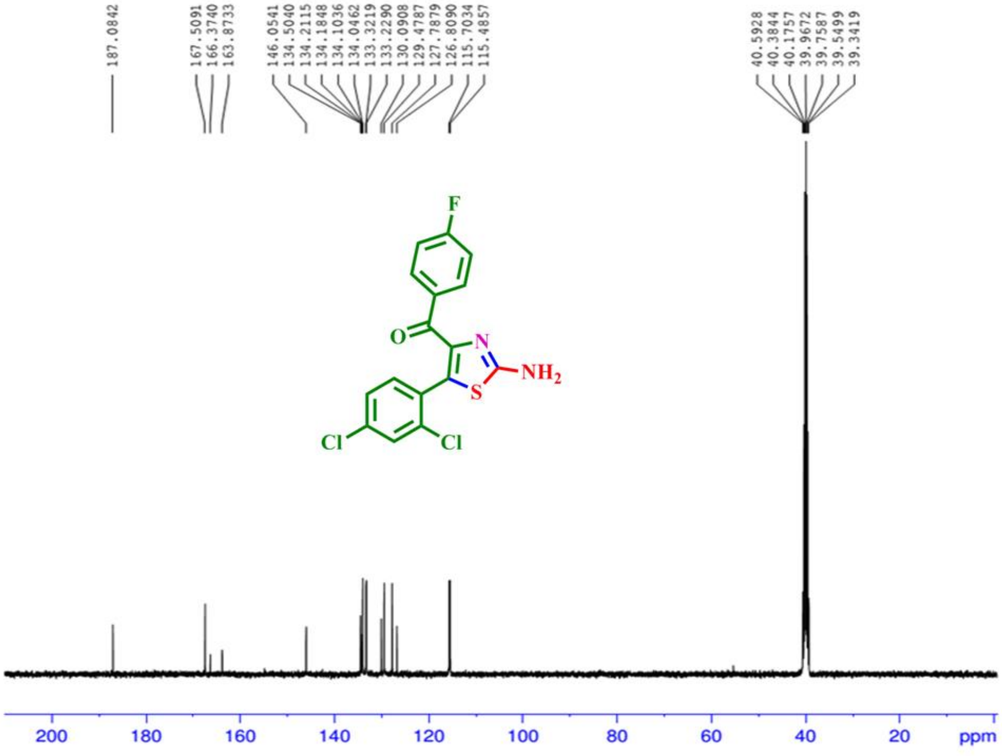

Figure S68. ^{13}C NMR spectrum of **4h**.

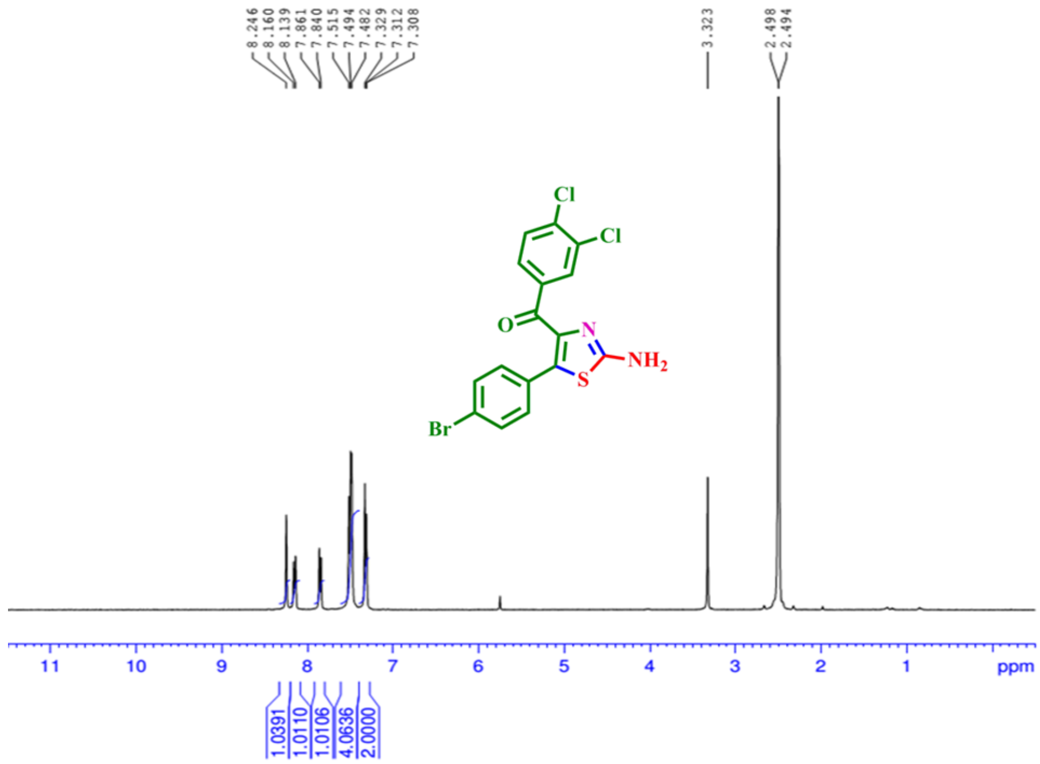

Figure S69. ^{1}H NMR spectrum of **4i**.

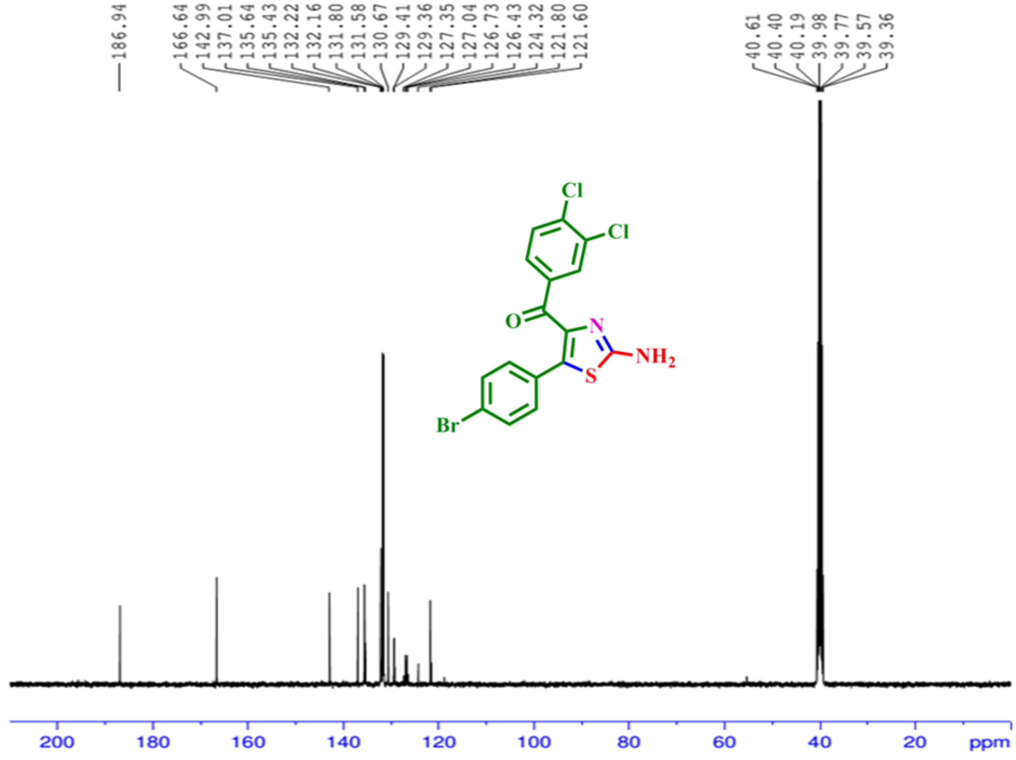

Figure S70. ^{13}C NMR spectrum of **4i**.

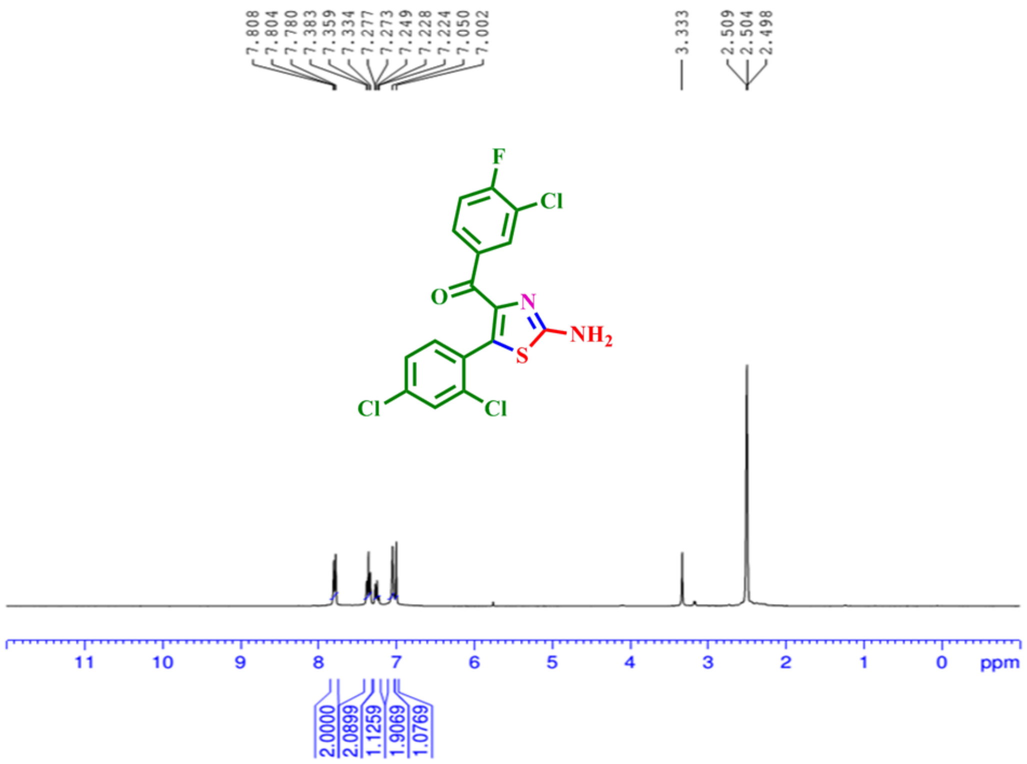

Figure S71. ^{1}H NMR spectrum of **4j**.

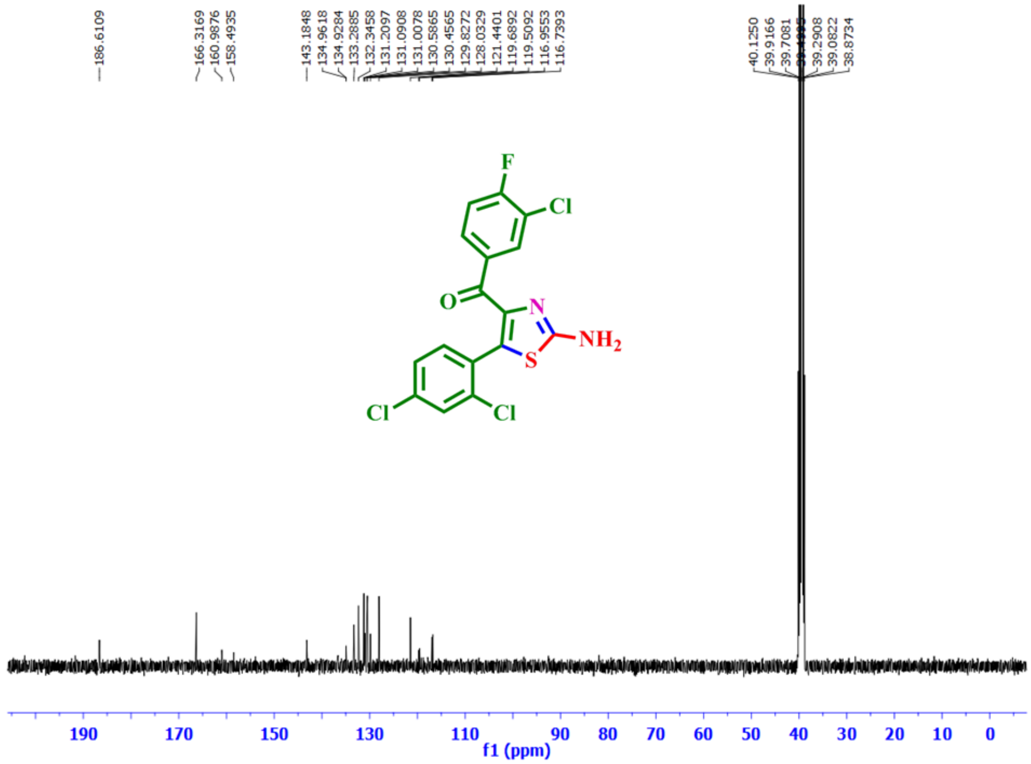

Figure S72. ^{13}C NMR spectrum of **4j**.

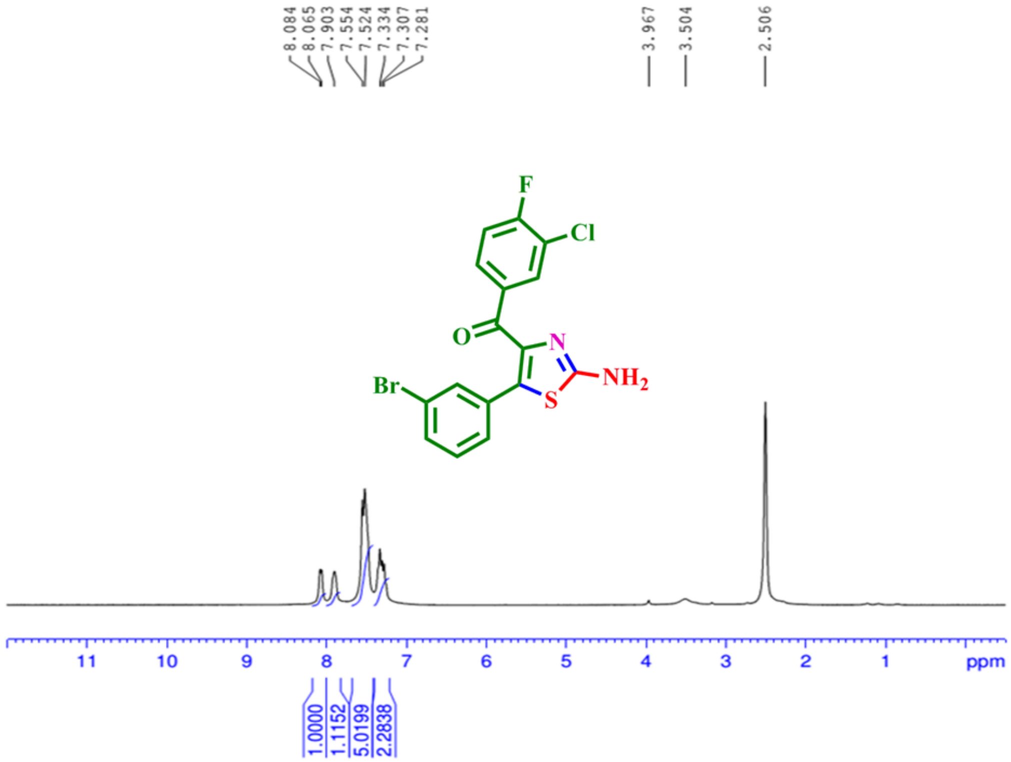

Figure S73. ^{1}H NMR spectrum of **4k**.

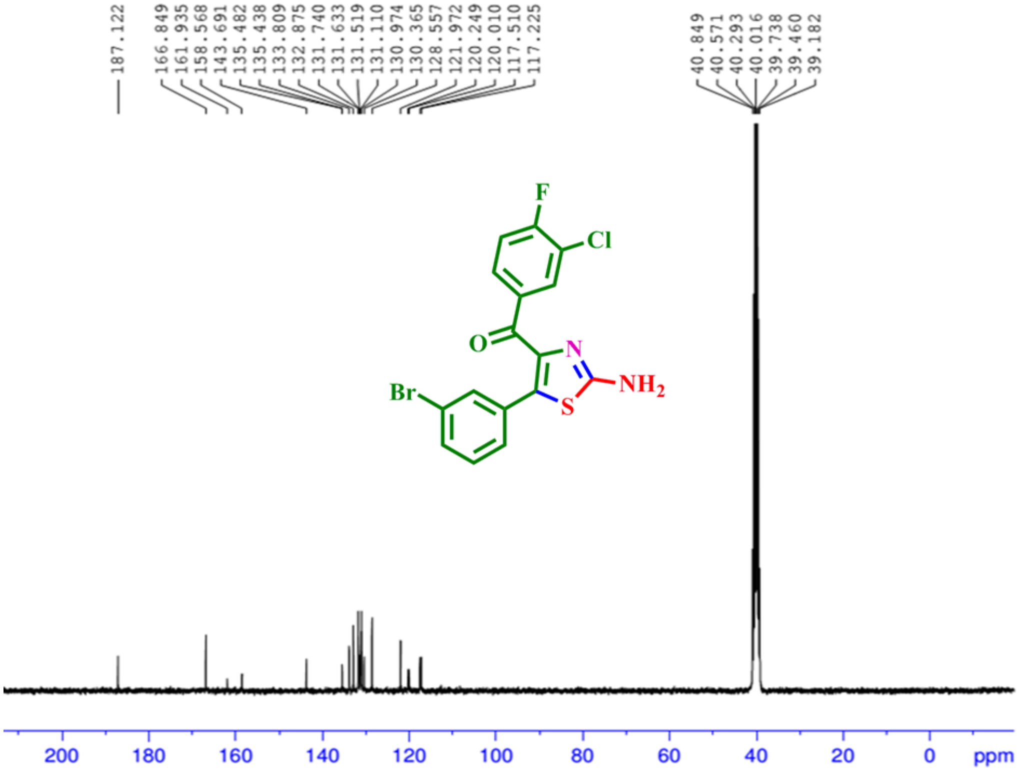

Figure S74. ^{13}C NMR spectrum of **4k**.

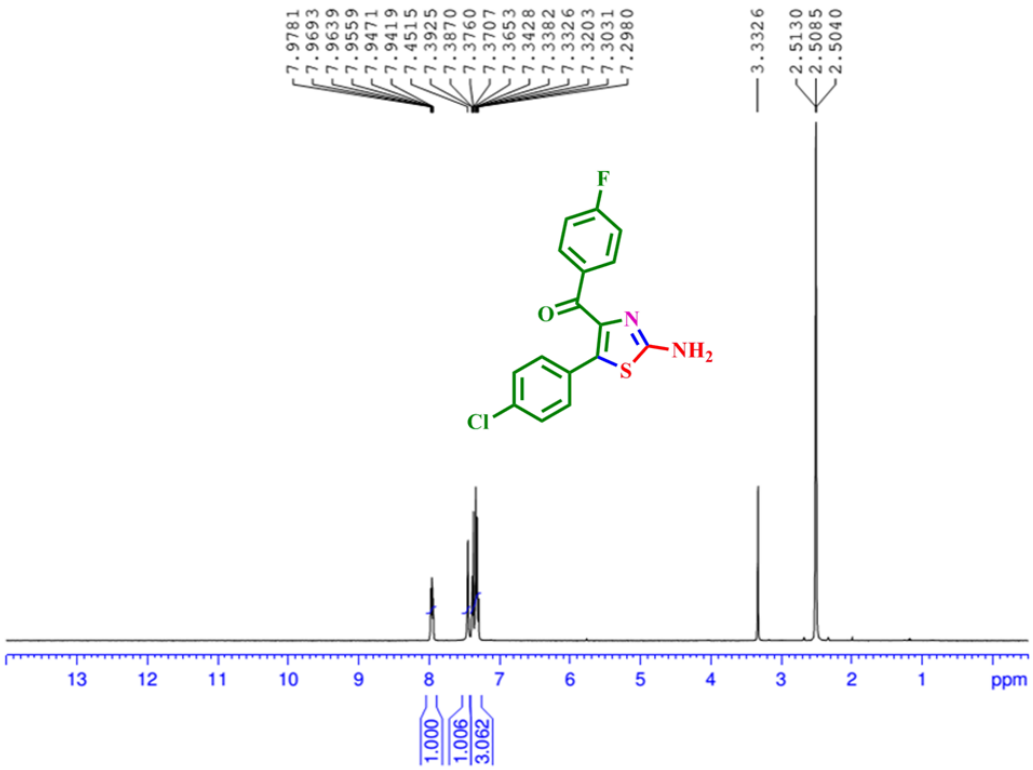

Figure S75. ^{1}H NMR spectrum of **4l**.

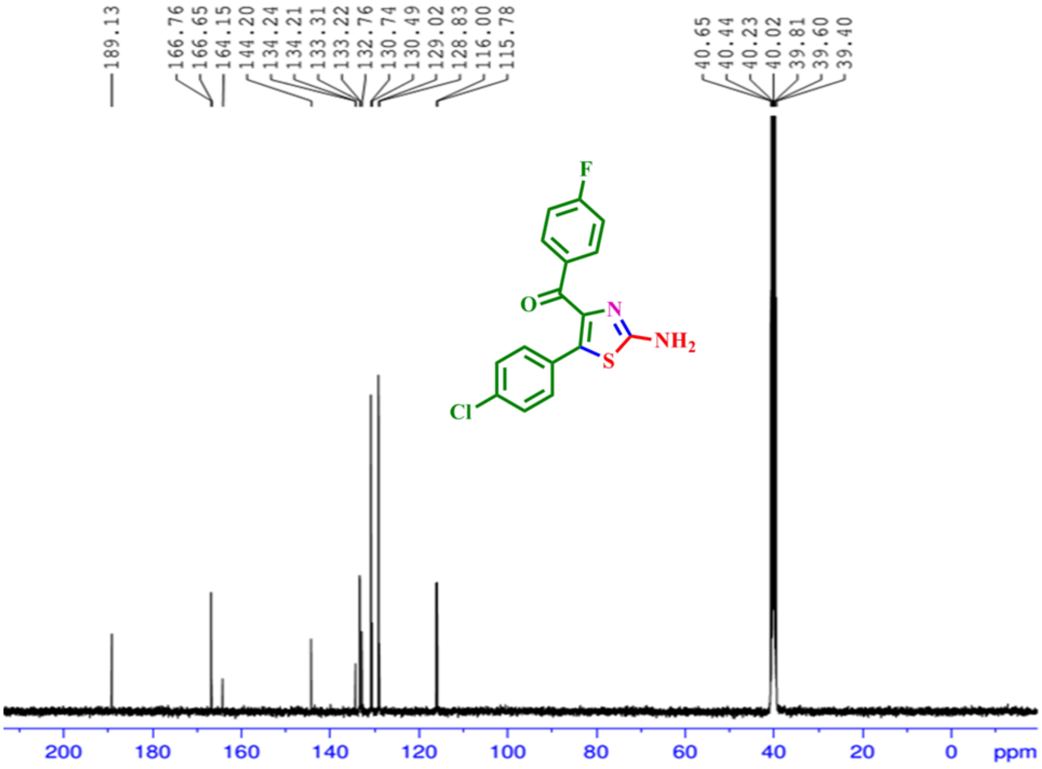

Figure S76. ^{13}C NMR spectrum of **4l**.

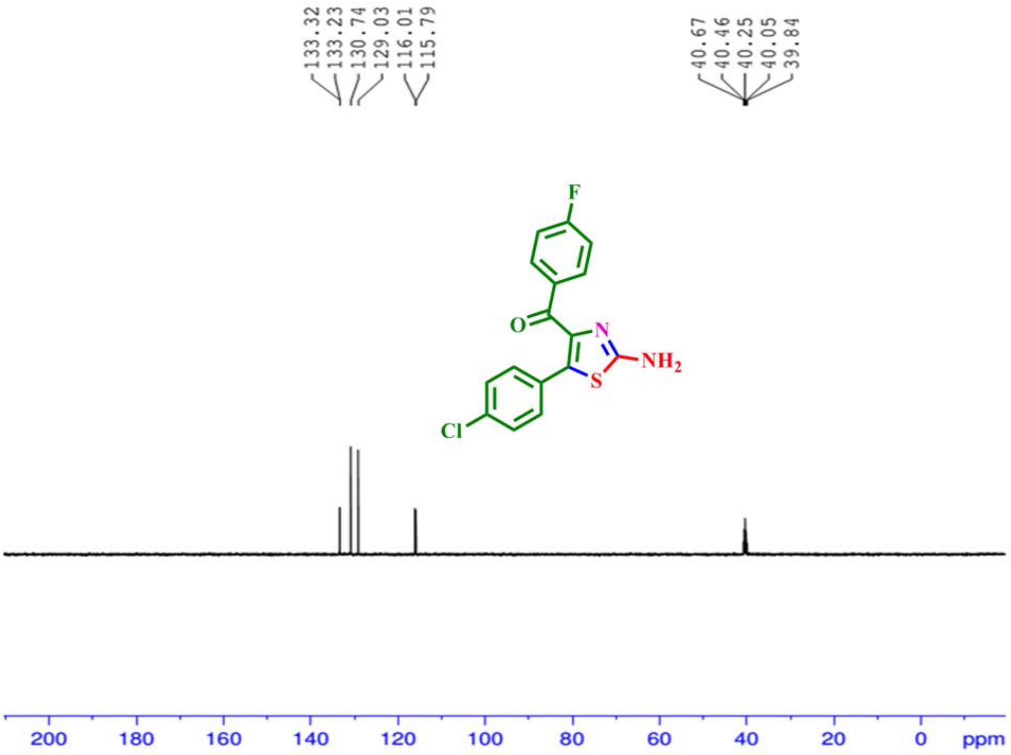

Figure S77. DEPT (135) spectrum of **4l**.

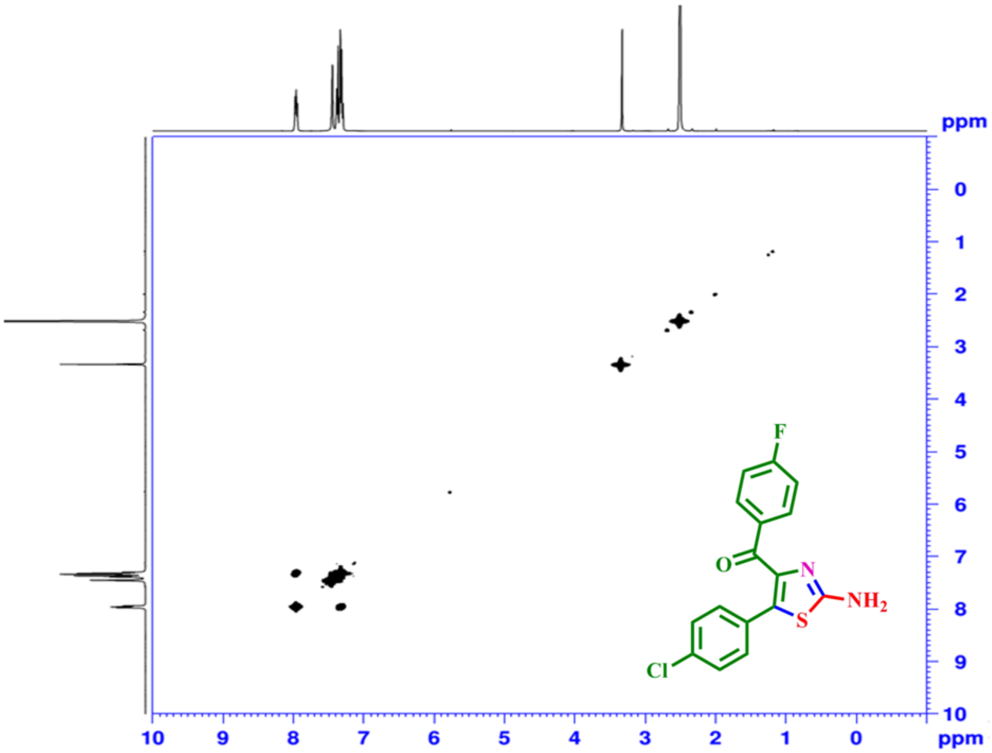

Figure S78. H-H COSY spectrum of **4l**.

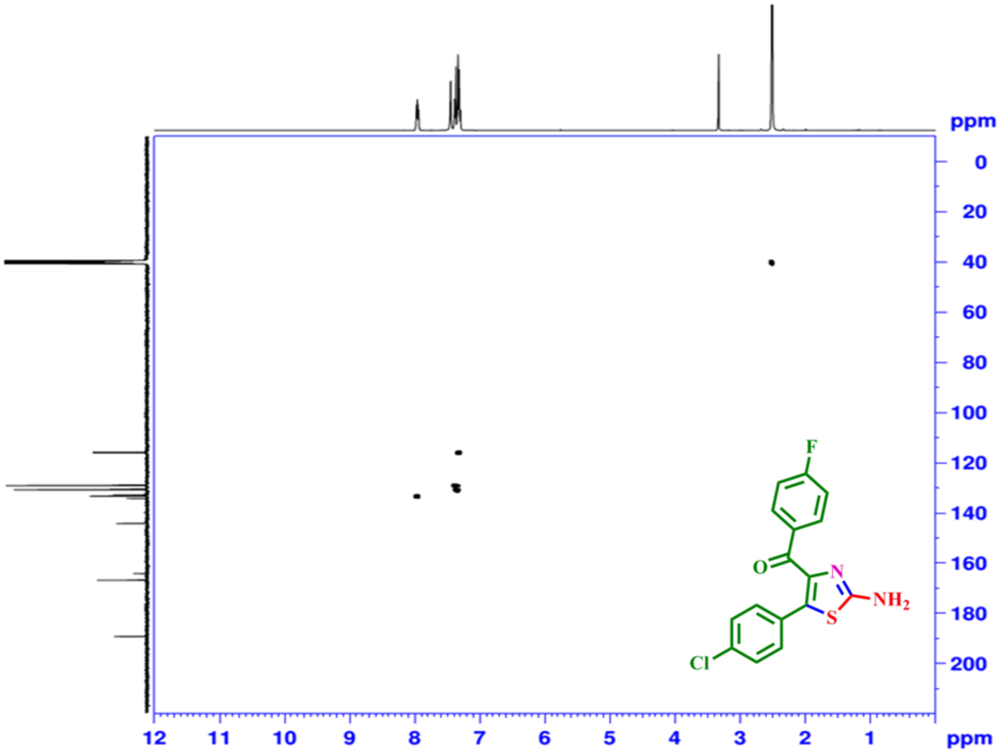

Figure S79. C-H COSY spectrum of **4l**.

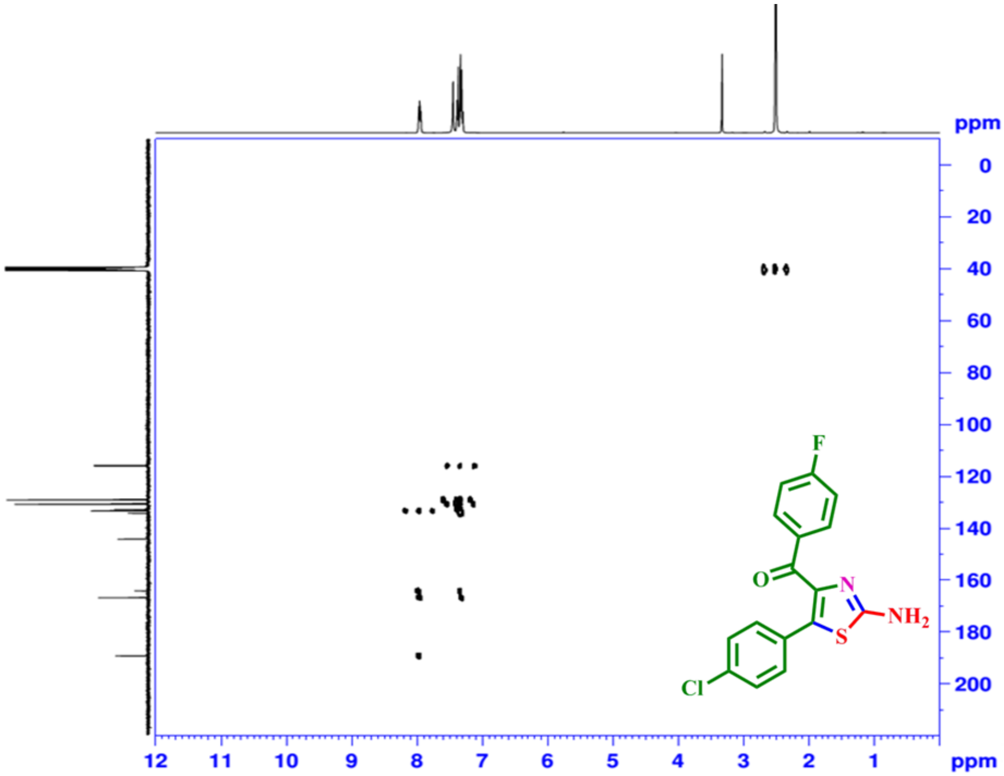

Figure S80. HMBC spectrum of **4l**.

VIII Reference

1. Zhang, G.; Chen, B.; Guo, X.; Guo, S.; Yu, Y. *Adv. Synth. Catal*. **2015**, *357*, 1065-1069.

.
